# Body composition in anorexia nervosa: Meta‐analysis and meta‐regression of cross‐sectional and longitudinal studies

**DOI:** 10.1002/eat.23158

**Published:** 2019-09-12

**Authors:** Christopher Hübel, Zeynep Yilmaz, Katherine E. Schaumberg, Lauren Breithaupt, Avina Hunjan, Eleanor Horne, Judit García‐González, Paul F. O'Reilly, Cynthia M. Bulik, Gerome Breen

**Affiliations:** ^1^ Social, Genetic & Developmental Psychiatry Centre Institute of Psychiatry, Psychology and Neuroscience, King's College London London UK; ^2^ UK National Institute for Health Research (NIHR) Biomedical Research Centre South London and Maudsley Hospital London UK; ^3^ Department of Medical Epidemiology and Biostatistics Karolinska Institutet Stockholm Sweden; ^4^ Department of Psychiatry University of North Carolina at Chapel Hill Chapel Hill North Carolina; ^5^ Department of Genetics University of North Carolina at Chapel Hill Chapel Hill North Carolina; ^6^ Department of Psychiatry University of Wisconsin—Madison Madison Wisconsin; ^7^ Eating Disorders Clinical and Research Program Massachusetts General Hospital Boston Massachusetts; ^8^ Department of Psychiatry Harvard Medical School Boston Massachusetts; ^9^ Department of Genetics, Faculty of Life Sciences and Medicine King's College London London UK; ^10^ School of Biological and Chemical Sciences Queen Mary, University of London London UK; ^11^ Department of Nutrition University of North Carolina at Chapel Hill Chapel Hill North Carolina

**Keywords:** BIA, binge‐eating/purging, bioelectrical impedance analysis, body fat percentage, bone, dual‐energy X‐ray absorptiometry, DXA, estradiol, fat‐free mass, insulin, lean mass, long‐term follow‐up, restricting, thyroid, weight restoration

## Abstract

**Objective:**

Clinically, anorexia nervosa (AN) presents with altered body composition. We quantified these alterations and evaluated their relationships with metabolites and hormones in patients with AN longitudinally.

**Method:**

In accordance with PRISMA guidelines, we conducted 94 meta‐analyses on 62 samples published during 1996–2019, comparing up to 2,319 pretreatment, posttreatment, and weight‐recovered female patients with AN with up to 1,879 controls. Primary outcomes were fat mass, fat‐free mass, body fat percentage, and their regional distribution. Secondary outcomes were bone mineral density, metabolites, and hormones. Meta‐regressions examined relationships among those measures and moderators.

**Results:**

Pretreatment female patients with AN evidenced 50% lower fat mass (mean difference [MD]: −8.80 kg, 95% CI: −9.81, −7.79, *Q* = 1.01 × 10^−63^) and 4.98 kg (95% CI: −5.85, −4.12, *Q* = 1.99 × 10^−28^) lower fat‐free mass, with fat mass preferentially stored in the trunk region during early weight restoration (4.2%, 95% CI: −2.1, −6.2, *Q* = 2.30 × 10^−4^). While the majority of traits returned to levels seen in healthy controls after weight restoration, fat‐free mass (MD: −1.27 kg, 95% CI: −1.79, −0.75, *Q* = 5.49 × 10^−6^) and bone mineral density (MD: −0.10 kg, 95% CI: −0.18, −0.03, *Q* = 0.01) remained significantly altered.

**Discussion:**

Body composition is markedly altered in AN, warranting research into these phenotypes as clinical risk or relapse predictors. Notably, the long‐term altered levels of fat‐free mass and bone mineral density suggest that these parameters should be investigated as potential AN trait markers.

**ResumenObjetivo:**

Clínicamente, la anorexia nervosa (AN) se presenta con alteraciones en la composición corporal. Cuantificamos estas alteraciones y evaluamos longitudinalmente su relación con metabolitos y hormonas en pacientes con AN.

**Método:**

De acuerdo con las pautas PRISMA, realizamos 94 meta‐análisis en 62 muestras publicadas entre 1996–2019, comparando hasta 2,319 pacientes mujeres en pre‐tratamiento, post‐tratamiento, y recuperadas en base al peso con hasta 1,879 controles. Las principales medidas fueron masa grasa, masa libre de grasa, porcentaje de grasa corporal y su distribución regional. Las medidas secundarias fueron densidad mineral ósea, metabolitos y hormonas. Las meta‐regresiones examinaron las relaciones entre esas medidas y moderadores.

**Resultados:**

Las pacientes femeninas con AN pre‐tratamiento mostraron un 50% menos de masa grasa (MD: −8.80 kg, CI 95%: −9.81, −7.79, *Q* = 1.01 × 10^–^^63^) y 4.98 kg (CI 95%: −5.85, −4.12, *Q* = 1.99 × 10^–^^28^) menos de masa libre de grasa, con masa grasa preferentemente almacenada en la región del tronco durante la recuperación temprana del peso (4.2%, CI 95%: −2.1, −6.2, *Q* = 2.30 × 10^–^^4^). Aunque la mayoría de los rasgos regresaron a los niveles vistos en los controles sanos después de la restauración del peso, la masa libre de grasa (MD: −1.27 kg, CI 95%: −1.79, −0.75, *Q* = 5.49 × 10^–^^6^) y la densidad mineral ósea (MD: −0.10 kg, CI 95%: −0.18, −0.03, *Q* = 0.01) permanecieron significativamente alteradas.

**Discusión:**

La composición corporal es marcadamente alterada en la AN, lo que garantiza la investigación en estos fenotipos como predictores de riesgo clínico o de recaída. Notablemente, la alteración a largo plazo de los niveles de masa libre de grasa y densidad mineral ósea sugieren que estos parámetros debe ser investigados como potenciales rasgos indicadores de AN.

**Abbreviations**
AN: anorexia nervosa
BIA: bioelectrical impedance analysis
DSM: Diagnostic and Statistical Manual of Mental Disorders
DXA: dual‐energy X‐ray absorptiometry
MD: mean difference
MRI: magnetic resonance imaging
NOS: Newcastle–Ottawa Scale

## 1 INTRODUCTION

Anorexia nervosa (AN) has one of the highest mortality rates of all psychiatric disorders (Chesney, Goodwin, & Fazel, 2014). Clinical observations show altered body composition (El Ghoch, Calugi, Lamburghini, & Dalle Grave, 2014; Solmi et al., 2016) accompanied by elevated cholesterol (Hussain et al., 2019) and greater insulin sensitivity (Ilyas et al., 2018). However, conclusions are limited by small sample sizes and consequent mixed findings.

Molecular genetic studies have revealed that individuals with AN carry genetic variants that increase their liability to AN and concurrently predispose them to lower body fat percentage, lower fasting insulin, and higher high‐density lipoprotein cholesterol concentrations, suggesting that metabolic factors may play an etiological role (Duncan et al., 2017; Watson et al., 2019). Additionally, longitudinal investigations of a British birth cohort showed that girls who develop AN later in life are already underweight at the age of 4 years when compared to healthy children (Yilmaz, Gottfredson, Zerwas, Bulik, & Micali, 2019), adding evidence for a developmental component.

A systematic review showed that adolescents and adults differently lose fat tissue when affected by AN, with adolescents losing more central fat tissue and adults more peripheral fat tissue. During weight recovery, individuals with AN show emergent central adiposity which typically attenuates over time (El Ghoch, Calugi, et al., 2014). These clinical and genetic findings encourage the meta‐analytic reassessment of the role of body composition traits, such as fat mass and fat‐free mass, their regional distribution, and their changes associated with weight restoration and long‐term weight recovery in AN.

Meta‐analyses have four major advantages compared to systematic reviews. Increasing statistical power through pooling results from independent samples leads to more precise estimates of the underlying effect. Meta‐analyses estimate the heterogeneity (i.e., inconsistency) among effect sizes from the individual studies included, which are crucial for the interpretation of the pooled estimates. Meta‐regressions are used to investigate potential moderators of the pooled effect sizes and the relationships between the outcomes of interest, while extensions of meta‐analytical models can estimate potential publication bias (Nakagawa, Noble, Senior, & Lagisz, 2017).

The goals of these meta‐analyses were to (a) replicate findings from the systematic review on fat mass; (b) extend the observations by quantifying them; (c) include fat‐free mass; (d) include bone mineral content and density; (e) investigate their associations with each other; and (f) if possible, relate findings to secondary outcomes, such as metabolic and hormonal parameters. This analytical approach is aimed at understanding the potential associations between these factors that are known to be physiologically interrelated. A thorough and rigorous examination of body composition and related laboratory parameters in individuals with AN could elucidate some of the physiological changes associated with this serious disorder, which could lead to more effective medical management, monitoring, and treatment approaches.

## 2 METHOD

### 2.1 Search strategy, selection criteria, and data extraction

**Figure 1:**
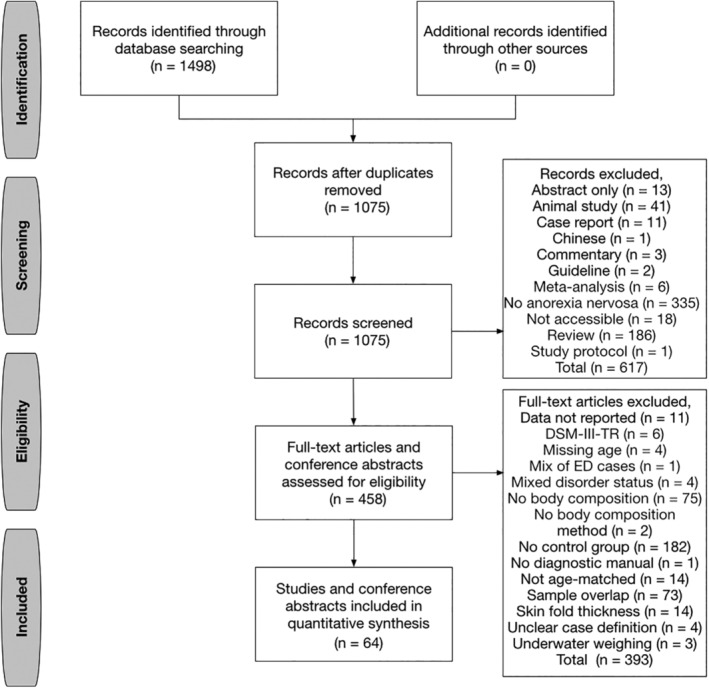
PRISMA (Preferred Reporting Items for Systematic Reviews and Meta‐Analyses) flow diagram of study selection

Our meta‐analysis was conducted according to PRISMA guidelines (Moher, Liberati, Tetzlaff, Altman, & PRISMA Group, 2009) and pre‐registered (PROSPERO 2018 CRD42018105338) with no changes to the protocol. We conducted a literature search from June 15, 2018, until July 15, 2019, using the electronic databases PubMed and Web of Science with a time limitation starting with articles published after January 1, 1994—marking the introduction of the Diagnostic and Statistical Manual of Mental Disorders, 4th Edition (DSM‐IV; American Psychiatric Association, 2013). We used key search terms including “anorexia nervosa” AND (“body composition” OR “body fat” OR “fat mass” OR “body fat percentage” OR DXA OR BIA OR “fat free mass” OR “lean mass”). The search was repeated by coauthors to avoid selection bias. Furthermore, we screened the references of published articles and reviews. Our search results, including the selection process, are presented in Figure 1 according to PRISMA guidelines. Our selection criteria are presented in Table 1. In case of multiple publications deriving from the same study population, we selected the articles reporting either the largest or the most recent data set. In case of conflict between these two criteria, large sample size was prioritized. We extracted the information presented in Table 1 from every identified study using a standardized data extraction sheet.

**Table 1:** Selection criteria and extracted data from the original publications

**Selection criteria** Studies investigating humans only Any age group No sample overlap Observational cross‐sectional or longitudinal studies or randomized‐controlled trials Clinical diagnoses of AN according to the DSM IV–5, or their revisions (American Psychiatric Association, 2013), or ICD‐10 (World Health Organization, 1992) Investigation of body composition by dual‐energy X‐ray absorptiometry (Bredella et al., 2010, 2013), bioelectrical impedance analysis (BIA) (Bonaccorsi et al., 2012; Mattar et al., 2011), dual photon absorptiometry, or magnetic resonance imaging (Mayer et al., 2005). Published or collected after January 1, 1994 (the year that DSM‐IV was introduced) The study includes a control group or comparison group Publications in any language which could be translated by the research team: English, German, Swedish, Danish, Spanish
**Extracted data** Author, publication year Country Sample sizes including gender and age Setting: Inpatient or outpatient Original longitudinal or cross‐sectional design Follow‐up period if longitudinal Diagnostic criteria: DSM‐IV, DSM‐IV‐TR, DSM‐5, or ICD‐10 Participant screening and exclusion criteria Number of cases: AN pretreatment, posttreatment (ANpost), recovered from AN (ANrec) Subtype of AN: Restricting (R), binge eating/purging Number of controls Primary outcome variables of body composition: Fat mass, fat‐free mass, body fat percentage, and their regional distribution Secondary outcome variables, which were reported by at least three studies additional to primary outcomes: Bone mineral density, glucose, insulin, ghrelin, adiponectin, leptin, insulin‐like growth factor, estradiol, testosterone, cortisol, thyroid‐stimulating hormone, free triiodothyronine, free thyroxine Covariates used in original analysis Fasting and fasting duration Blood sample: Serum, plasma, or unspecified Medication and contraceptives Psychological and additional treatments Outcome was a secondary or primary outcome in the original study Duration of illness Age at diagnosis/onset Age at menarche Percentage of AN cases with amenorrhea and duration of amenorrhea

Abbreviations: AN, anorexia nervosa; ICD‐10, International Classification of Diseases version 10; DSM, Diagnostic and Statistical Manual of Mental Disorders.

The data extraction sheet was based on two previous meta‐analyses (Hussain et al., 2019; Ilyas et al., 2018) and included variables that were hypothesized to be associated with body composition, hormonal, or metabolic measures, including fasting status and period, medications, stage of the menstrual cycle, or treatments for longitudinal studies. If enough studies reported these variables, we performed meta‐regressions to investigate their associations with our primary and secondary outcomes.

### 2.2 Quality of study assessment (Newcastle–Ottawa scale)

We used the Newcastle–Ottawa Scale (NOS) to assess the quality of nonrandomized studies (Wells et al., 2009). Each study is judged on three broad perspectives: (a) the selection of the study groups; (b) the comparability of the groups; and (c) the ascertainment of the outcome of interest for case–control studies. The NOS evaluates these three quality parameters divided across eight specific items. Each item on the scale is scored from one point, except for comparability, which can be adapted to the specific topic of interest to score up to two points. It has been designed to be used in meta‐analyses and systematic reviews. For the observational studies, low quality was defined as NOS score ≤8.0 and high quality as score >8.0 (maximum score 9).

### 2.3 Meta‐analysis

Inverse variance‐weighted meta‐analyses for females and males separately were conducted using the statistical package “meta” and “metafor” in the open‐source software R v3.5.1 (http://r-project.org). We used additional formulas to calculate missing values (Hozo, Djulbegovic, & Hozo, 2005; Luo, Wan, Liu, & Tong, 2018; Wan, Wang, Liu, & Tong, 2014). As effect sizes, we estimated mean differences (MDs) between individuals with AN and controls. We chose a random‐effects model, which assumes that the heterogeneity in the differences between clinical and control groups is due to both within‐study and between‐study variation, as we anticipated differences in procedures and study populations between studies. We quantified the heterogeneity through a restricted maximum‐likelihood (REML) approach. For the analysis of subtypes, posttreatment, and weight‐recovered patients with AN, the control groups from the acutely‐ill/pretreatment analysis were reused because (a) control groups were not measured repeatedly and (b) none of the studies had separate control groups for each subtype analysis. Although some studies included covariates in their statistical analysis (Bratland‐Sanda et al., 2010; Bredella et al., 2008; Dellava, Policastro, & Hoffman, 2009; DiVasta et al., 2007; Fernández‐Soto, González‐Jiménez, Chamorro‐Fernández, & Leyva‐Martínez, 2013; Haas et al., 2005; Karlsson, Weigall, Duan, & Seeman, 2000; Kosmiski, Schmiege, Mascolo, Gaudiani, & Mehler, 2014; Maïmoun et al., 2018; Nakahara et al., 2007; Schneider et al., 1998), we only used raw values without including study‐specific covariates to increase comparability across individual studies. Weight recovery was defined in accordance with DSM‐IV and DSM‐5 criteria with BMI >18.5 kg/m^2^ or >90% ideal body weight. To correct our primary analysis for multiple testing, false discovery rate–adjusted *Q values* were calculated (Benjamini & Hochberg, 1995).

### 2.4 Detection and adjustment for publication bias

The results of meta‐analyses can be influenced by publication bias (i.e., small study effects). This describes the phenomenon when certain studies have been selected for publication, while others—mostly due to negative findings—have not been published (Nieminen, Rucker, Miettunen, Carpenter, & Schumacher, 2007). Through graphical diagnosis of asymmetry in funnel plots (Egger, Smith, Schneider, & Minder, 1997) and performing Thompson and Sharp tests (i.e., weighted linear regressions) that take variation between studies into account (Thompson & Sharp, 1999), we investigated potential small study effects or publication bias. If the test resulted in a *p* value below .05, we adjusted the pooled effect estimates using a Copas selection model calculated with the R package “metasens.” The model has two components: the first component estimates the pooled effect, while the second estimates a publication probability for each study. A large correlation between these two components suggests that studies with more extreme effects were more likely to be published (Copas, 1999; Copas & Shi, 2000, 2001). The models were iteratively optimized using two tuning parameters γ_0_ and γ_1_. We present four diagnostic graphics including (a) a funnel plot, (b) a contour plot, (c) a treatment effect plot, and (d) a *p* value plot.

### 2.5 Investigation of potential moderators through meta‐regression and stratification

To examine the large between‐study heterogeneity per meta‐analysis (Table 1), we performed meta‐regressions using mixed effects models included in the R package “meta” that take the heterogeneity within and between individual studies into account. The models were optimized via a REML approach. Through meta‐regression, we investigated whether relevant participant or study characteristics may be associated with the pooled estimates, such as mean age, the time period of follow‐up for longitudinal studies, age at diagnosis, age at menarche, age at amenorrhea, duration of illness, percentage of amenorrhea in patients with AN, percentage of medicated patients with AN, percentage of individuals taking contraceptives, body composition measurement method, blood sample type, body composition parameters, and their differences between cases and controls.

A second approach to test for potential moderators is stratification of the sample into meaningful subgroups and estimation of statistical differences between the pooled estimates per subgroup. We used this approach and stratified by AN subtype.

## 3 RESULTS

### 3.1 Results of the search and selection of studies

A total of 1,498 papers published between 1996 and 2019 were identified by our search terms, and 1,434 (96%) of them were excluded. No paper published during 1994–1996 fulfilled the inclusion criteria, and the most common reasons for exclusion apart from not investigating AN or being a review were (a) no control group (*n* = 182, 12%); (b) no main outcome reported (i.e., body composition; *n* = 75, 5%); and (c) sample overlap (*n* = 73, 5%). Detailed exclusion process is presented in Figure 1. Sixty‐four published articles (4%) were included in our analysis, and we became aware of no additional unpublished samples after contacting study authors for additional or missing data (Table S1). The majority of studies focused on female cases and controls that were sampled consecutively in only 22 of 62 samples (35%, Table S2) and aged between 13.8 and 31.3 years (Figure S1). As such, four studies (6%) investigating male AN cases were investigated in a separate quantitative synthesis and are discussed briefly (El Ghoch, Calugi, Milanese, Bazzani, & Dalle Grave, 2017; Marra et al., 2019; Misra et al., 2013; Schorr et al., 2019). Three studies (5%) originated from Australasia, 38 (61%) from Europe, 15 (24%) from North America, and 6 (10%) from Asia. Only 13 studies (21%) used the same method of ascertainment for cases and controls (Table S2). Twenty‐nine studies (47%) investigated inpatients, 8 (13%) outpatients, 2 (3%) a mixture of both, and 23 studies (37%) did not specify the recruitment or patient‐setting. Twenty‐seven studies (44%) comprised collection of blood samples after a fasting period, whereas only six studies (10%) specified the fasting period (Bredella et al., 2012; DiVasta et al., 2011; Dostálová, Sedlácková, Papezová, Nedvídková, & Haluzík, 2009; Estour et al., 2017; Kaválková et al., 2012; Prioletta et al., 2011). One study (3%) did not specify whether analyses were performed using plasma or serum blood (Weinbrenner et al., 2004). Seventeen studies (27%) sampled regular menstruating participants during the follicular phase of their cycle (de Alvaro et al., 2007; Dostálová et al., 2009; Estour et al., 2017; Galusca et al., 2015; Germain et al., 2010, 2007, 2016; Grinspoon et al., 2001; Kaválková et al., 2012; Kirchengast & Huber, 2004; Mayer et al., 2005, 2009; Nakai, Hamagaki, Takagi, Taniguchi, & Kurimoto, 1999; Prioletta et al., 2011; Scalfi et. al, 2002; Weinbrenner et al., 2004), whereas 14 studies (23%) did not provide details about the cycle phase (Bachmann et al., 2014; Bredella et al., 2012; Delporte, Brichard, Hermans, Beguin, & Lambert, 2003; DiVasta et al., 2011; Fazeli et al., 2010; Fernández‐Soto et al., 2013; Germain et al., 2010; Gniuli, Liverani, Capristo, Greco, & Mingrone, 2001; Grinspoon et al., 1996; Guo, Jiang, Liao, Liu, & He, 2013; Haas et al., 2005; Karczewska‐Kupczewska et al., 2010; Maïmoun et al., 2018; Mörkl et al., 2017; Nakahara et al., 2007; Rigaud, Boulier, Tallonneau, Brindisi, & Rozen, 2010; Tagami et al., 2004; Tanaka et al., 2003). However, studies were retained to achieve the largest possible sample size, and––depending on data availability––meta‐regressions were fitted to investigate study characteristics as possible moderators. Originally, 41 studies (66%) were cross‐sectional and 21 were longitudinal (34%, Table S1). However, four of the longitudinal studies (19%) were analyzed cross‐sectional in our meta‐analysis due to missing data. No control group was repeatedly measured in any of the longitudinal studies.

### 3.2 Characteristics of the included studies

We performed four sets of meta‐analyses (a) comparing 2,319 pretreatment/acutely ill AN patients with 1,879 healthy controls; (b) comparing 722 post‐treatment AN patients with 809 controls; (c) estimating the change in AN patients (*n* = 722) from pretreatment to posttreatment; and (d) comparing 398 weight‐recovered individuals with AN with 660 healthy controls including samples with a long‐term follow‐up. The pretreatment AN group comprised 229 individuals suffering from the binge‐eating/purging (8% of cases) and 701 from the restricting subtype (26% of cases). The shortest follow‐up period was 5.14 weeks, and the longest was 2 years (Table S1). Twenty studies (32%) used bioelectrical impedance analysis (BIA) to assess body composition, 39 (63%) used dual‐energy X‐ray absorptiometry (DXA), and only 3 (5%) utilized magnetic resonance imaging (MRI)––considered to be the benchmark. Thirty of the 62 studies (48%) investigated body composition as a primary outcome, whereas it was a secondary outcome in the remaining studies. The percentage of AN patients with amenorrhea ranged from 0 to 100%, with 11 studies (18%) not providing information on menstrual status (Agüera et al., 2015; Bachmann et al., 2014; Bredella et al., 2012; de Mateo Silleras et al., 2013; El Ghoch et al., 2012; Gniuli et al., 2001; Iacopino et al., 2003; Kirchengast & Huber, 2004; Schneider et al., 1998; Tagami et al., 2004; Tanaka et al., 2003). Thirty‐five of 62 studies (56%) did not provide information on the medication status of AN patients, and 32 (52%) did not indicate whether oral contraceptives were used. In AN cases, the duration of illness was on average 52.2 months (*SD* = 29.4), the duration of amenorrhea 23.0 months (*SD* = 18.3), and the age at diagnosis 17.5 years (*SD* = 3.0). Cases and controls were well matched for age (Figure S1) and, notably, we did not observe a difference in age at menarche (Figure S2) or height (Figure S5) between AN cases and controls.

### 3.3 Data and analyses results of meta‐analyses and meta‐regressions

Our results from the 94 meta‐analyses show that a wide range of alterations in several key body composition and biochemical measures exist in AN cases compared with healthy controls (Figure 2 and Figure S3). For 95% confidence intervals and *Q* values, heterogeneity estimates (*τ* ^2^ and *I* ^2^), and adjusted estimates due to estimated publication bias, see Table 2. Detailed forest plots showing each of the 94 meta‐analyses are presented as Figures S4–S89 for females and Figures S90–S95 for males. No differences between restricting and binge‐eating/purging subtype of AN were detected in our meta‐analysis prior to treatment except for total body water (Table S3). Between‐study heterogeneity (*I* ^2^) was observed in 62 meta‐analyses (70%) and ranged from 52 to 99%, confirming our choice of a random‐effects model. To investigate moderators implicated in heterogeneity, we performed 411 meta‐regressions (Tables S4–S7). Six meta‐analyses showed funnel plot asymmetry, indicating small study effects. Therefore, we fitted Copas models to adjust for those effects and estimate the probable number of unpublished studies (Table 1 and Figures S96–S101).

**Figure 2:**
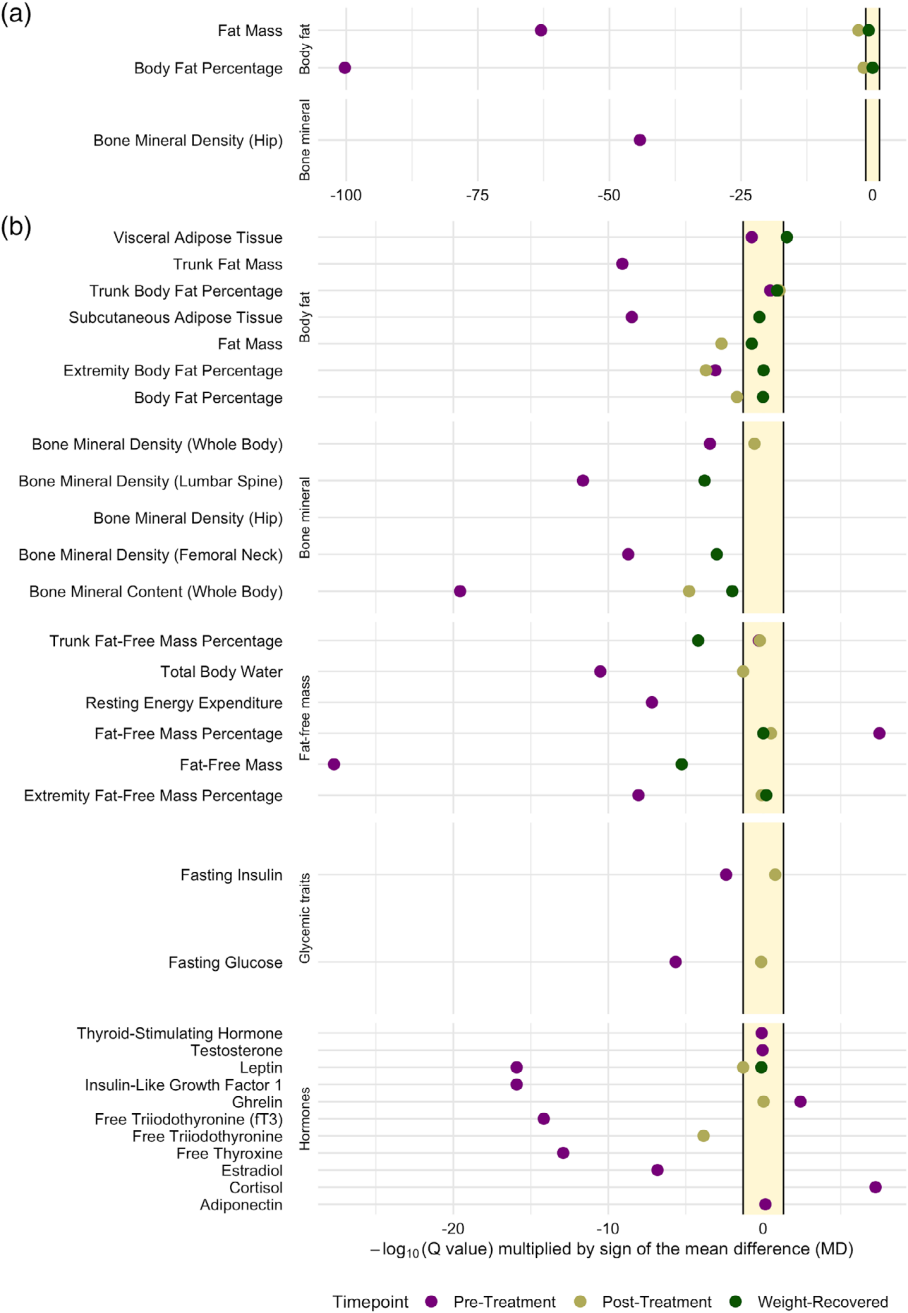
Summary plot of all 88 meta‐analyses comparing female AN cases with healthy controls. The plot shows the *Q* values (i.e., the false discovery rate‐corrected *p* values) of each inverse variance‐weighted random‐effects meta‐analyses comparing AN cases pretreatment (purple, *n* = up to 2,294), posttreatment (light green, *n* = up to 722), and after weight recovery (dark green, *n* = up to 398) with healthy controls (*n* = up to 2,251). Restricted maximum‐likelihood estimator was used to estimate heterogeneity. *Q* values are transformed on the ‐log_10_ scale, multiplied by the sign of the mean difference (MD) and presented on the *x*‐axis. Points lying in the yellow area indicate no statistically significant mean difference between AN cases and healthy controls after correction for multiple testing (i.e., *Q* > 0.05). Points to the left of the yellow area indicate a lower mean value in AN cases than in controls, whereas points on the right of the yellow area indicate a higher mean value in AN cases than in controls. A dark green point outside the yellow area indicates a significant difference between AN cases and controls after weight recovery (a) The outcomes with the largest differences between cases and controls. (b) The less extreme mean differences. The *x*‐axis was capped at ‐log_10_(*Q* value) × sign(MD) = −0.25. The full figure is presented as Figure S3

**Table 2:** Overview table over all 94 fitted inverse‐variance weighted random‐effects meta‐analyses comparing female or male anorexia nervosa (AN) patients and healthy controls (CO) pretreatment, posttreatment, and after weight recovery and additional meta‐analyses estimating the change in female AN patients before and after treatment

Female		Number of participants				Meta‐analysis					Heterogeneity				Small study effects					
Pretreatment outcome	*k*	AN	CO	Min	Max	MD	Unit	95% CI	*p*	*Q*	*τ* ^2^	*I* ^2^	95% CI	*p*	T&S	Copas	95% CI	*p*	*N* unpub	Reference values
Weight	36	1,444	1,536	−26.60	−4.00	−15.64	kg	−16.98, −14.30	1.27 × 10^−115^	5.59 × 10^−114^	13.41	84.6%	79.6%, 88.4%	4.12 × 10^−30^	0.76					
Height	26	1,499	1,255	−0.05	0.03	−0.01	m	−0.02, 0.00	.01	0.02	0.0001	66.7%	49.7%, 77.9%	6.86 × 10^−7^	0.12					
Body mass index	56	2,742	2,302	−9.90	−2.10	−5.81	kg/m^2^	−6.25, −5.38	3.22 × 10^−154^	2.83 × 10^−152^	2.43	93.4%	92.2%, 94.5%	2.27 × 10^−140^	0.10					18.5–24.9 kg/m^2^
Fat mass	40	2,193	1,720	−20.50	−1.54	−8.80	kg	−9.81, −7.79	4.58 × 10^−65^	1.01 × 10^−63^	9.64	96.0%	95.3%, 96.7%	2.97 × 10^−181^	0.59					
Body fat percentage	44	2,179	1803	−24.60	−5.50	−13.84	%	−15.10, −12.58	1.87 × 10^−102^	5.49 × 10^−101^	15.84	92.8%	91.2%, 94.1%	1.40 × 10^−98^	0.31					20%–25%
Visceral adipose tissue	2	44	115	−1.02	−0.21	−0.62	kg	−1.41, 0.18	.13	0.18	0.32	99.0%	97.9%, 99.5%	1.67 × 10^−22^						
Subcutaneous adipose tissue	2	44	115	−10.71	−7.70	−9.26	kg	−12.21, −6.31	7.46 × 10^−10^	3.28 × 10^−9^	4.24	93.5%	78.9%, 98.0%	8.63 × 10^−5^						
Trunk fat mass	4	72	88	−4.50	−2.40	−3.51	kg	−4.58, −2.43	1.65 × 10^−10^	8.07 × 10^−10^	0.88	73.8%	26.7%, 90.7%	.009	0.83					
Trunk body fat percentage	7	199	245	−5.65	6.40	1.77	%	−1.46, 5.01	.28	0.36	16.72	91.1%	84.2%, 95.0%	1.36 × 10^−12^	0.96					
Extremity body fat percentage	5	129	124	−9.00	0.53	−5.40	%	−8.38, −2.43	3.74 × 10^−4^	8.03 × 10^−4^	8.47	71.5%	27.8%, 88.7%	7.25 × 10^−3^	0.37					
Fat‐free mass	37	2,319	1879	−12.16	0.20	−4.98	kg	−5.85, −4.12	1.36 × 10^−29^	1.99 × 10^−28^	5.92	90.5%	87.9%, 92.5%	1.22 × 10^−58^	0.51					
Fat‐free mass percentage	9	562	528	−0.10	20.47	12.29	%	8.12, 16.47	8.03 × 10^−9^	3.21 × 10^−8^	39.60	99.6%	99.6%, 99.7%	.00	0.21					
Trunk fat‐free mass percentage	3	124	133	−2.41	0.20	−0.19	%	−0.66, 0.27	.42	0.52	0.00	55.8%	0.0%, 87.4%	.10						
Extremity fat‐free mass percentage	3	124	133	−1.84	−1.00	−1.53	%	−2.03, −1.03	2.11 × 10^−9^	8.84 × 10^−9^	0.00	0.0%	0.0%, 81.7%	.57						
Bone mineral content (whole body)	6	585	358	−0.70	−0.11	−0.16	kg	−0.19, −0.12	3.10 × 10^−21^	2.73 × 10^−20^	0.00	58.2%	0.0%, 83.1%	.04	0.08					
Bone mineral density (whole body)	15	1,000	593	−0.41	0.04	−0.07	g/cm^2^	−0.11, −0.04	1.64 × 10^−4^	3.61 × 10^−4^	0.005	80.3%	68.3%, 87.7%	1.30 × 10^−9^	0.002	−0.03	−0.06, −0.01	0.02	9	
Bone mineral density (lumbar spine)	12	871	682	−0.27	0.01	−0.14	g/cm^2^	−0.18, −0.10	4.22 × 10^−13^	2.32 × 10^−12^	0.004	83.2%	72.0%, 89.9%	9.28 × 10^−10^	0.87					
Bone mineral density (femoral neck)	11	1,109	723	−0.28	0.03	−0.14	g/cm^2^	−0.18, −0.09	4.16 × 10^−10^	1.93 × 10^−9^	0.004	85.9%	76.5%, 91.5%	2.98 × 10^−11^	0.81					
Bone mineral density (hip)	7	945	406	−0.15	0.03	−0.13	g/cm^2^	−0.15, −0.11	3.52 × 10^−46^	6.20 × 10^−45^	0.0002	52.0%	0.0%, 79.6%	.05	0.22					
Total body water	6	342	254	−6.82	−2.60	−4.77	L	−6.13, −3.41	5.92 × 10^−12^	3.06 × 10^−11^	1.95	78.3%	52.1%, 90.1%	3.36 × 10^−4^	0.72					3.3–3.6 L
Resting energy expenditure	3	99	128	−536.00	−296.37	−393.95	kcal/day	−531.04, −256.86	1.78 × 10^−8^	6.53 × 10^−8^	12,736.55	85.9%	59.0%, 95.2%	8.30 × 10^−4^						
Fasting glucose	7	111	107	−26.49	−7.21	−11.44	mg/dl	−15.95, −6.93	6.71 × 10^−7^	2.21 × 10^−6^	28.76	73.9%	44.2%, 87.8%	8.04 × 10^−4^	0.03	−7.01	−9.61, −4.40	<0.0001	11	<140 mg/dl
Fasting insulin	9	222	221	−42.01	7.83	−19.23	pmol/L	−31.68, −6.77	.002	0.004	282.08	91.2%	85.5%, 94.6%	3.43 × 10^−16^	0.40					<173.6 pmol/L
Ghrelin	4	123	83	15.00	217.50	149.20	pmol/L	54.59, 243.81	.002	0.004	7,872.55	88.3%	72.4%, 95.0%	1.19 × 10^−5^	0.61					114.4–154 pmol/L
Adiponectin	4	104	88	−7.30	7.28	1.37	μg/ml	−4.36, 7.11	.64	0.73	30.55	86.6%	67.4%, 94.4%	5.62 × 10^−5^	0.52					4–37 μg/ml
Leptin	19	771	544	−14.10	−0.46	−7.90	ng/ml	−9.72, −6.08	1.55 × 10^−17^	1.22 × 10^−16^	14.25	94.1%	92.0%, 95.6%	6.47 × 10^−54^	0.04	−7.20	−8.44, −5.96	<0.0001	5	3.3–18.3 ng/ml
Testosterone	4	99	98	−18.23	18.30	−1.35	ng/dl	−16.03, 13.33	.86	0.90	144.92	69.4%	11.9%, 89.4%	.02	0.91					23–75 ng/dl
Thyroid‐stimulating hormone	5	188	196	−0.80	0.30	−0.06	μIU/ml	−0.40, 0.27	.72	0.79	0.08	55.5%	0.0%, 83.6%	.06	0.30					0.4–4.8 μIU/L
Free triiodothyronine	8	251	215	−2.14	−0.73	−1.32	pmol/L	−1.64, −1.00	1.09 × 10^−15^	6.85 × 10^−15^	0.17	85.7%	73.7%, 92.2%	2.41 × 10^−8^	0.14					3.5–9.5 pmol/L
Free thyroxine	4	173	177	−3.40	−2.19	−2.60	pmol/L	−3.26, −1.93	2.09 × 10^−14^	1.23 × 10^−13^	0.19	36.8%	0.0%, 78.2%	.19	0.88					13–27 pmol/L
Insulin‐like growth factor 1	9	233	186	−140.30	−41.00	−95.86	ng/ml	−117.93, −73.8	1.67 × 10^−17^	1.22 × 10^−16^	747.86	72.2%	45.3%, 85.8%	3.50 × 10^−4^	0.91					130–450 ng/ml
Cortisol	7	209	167	50.00	232.00	131.92	nmol/L	86.26, 177.58	1.49 × 10^−8^	5.70 × 10^−8^	2,649.65	72.6%	40.9%, 87.3%	.001	0.85					170–635 nmol/L (8:00 a.m.)
Estradiol	11	278	231	−72.41	−1.86	−40.83	pg/ml	−55.43, −26.23	4.22 × 10^−8^	1.49 × 10^−7^	526.46	99.1%	98.9%, 99.3%	9.60 × 10^−237^	0.87					20–50 pg/ml

Male		Number of participants				Meta‐analysis					Heterogeneity				Small study effects					
Pretreatment	*k*	AN	CO	Min	Max	MD	Unit	95% CI	*p*	*Q*	*τ* ^2^	*I* ^2^	95% CI	*p*	T&S	Copas	95% CI	*p*	*N* unpub	Reference values
Weight	2	32	34	−18.50	−11.33	−15.48	kg	−22.42, −8.54	1.22 × 10^−5^	1.80 × 10^−05^	17.94	69.8%	0.0%, 93.2%	.07						
Height	3	42	44	−0.03	0.00	−0.02	m	−0.04, 0.01	.13	0.13	0.00	0.0%	0.0%, 82.7%	.55						
Body mass index	4	68	92	−9.00	−3.70	−5.48	kg/m^2^	−7.87, −3.09	6.92 × 10^−06^	1.80 × 10^−05^	5.58	94.2%	88.2%, 97.1%	3.65 × 10^−11^	0.89					18.5–24.9 kg/m^2^
Fat mass	3	42	44	−8.70	−3.03	−5.87	kg	−8.98, −2.75	2.22 × 10^−04^	2.70 × 10^−04^	6.77	87.2%	63.5%, 95.5%	4.17 × 10^−04^						
Body fat percentage	4	68	92	−9.70	−2.40	−7.49	%	−10.79, −4.19	8.76 × 10^−06^	1.80 × 10^−05^	9.84	85.7%	64.8%, 94.2%	1.07 × 10^−04^	0.70					12%–14%
Fat‐free mass	3	42	44	−10.20	−5.25	−9.37	kg	−12.47, −6.27	3.30 × 10^−09^	2.00 × 10^−08^	0.00	0.0%	0.0%, 66.3%	.73						

Female		Number of participants				Meta‐analysis					Heterogeneity				Small study effects					
Posttreatment	*k*	AN	CO	Min	Max	MD	Unit	95% CI	*p*	*Q*	*τ* ^2^	*I* ^2^	95% CI	*p*	T&S	Copas	95% CI	*p*	*N* unpub	Reference values
Weight	12	398	492	−16.60	0.60	−4.92	kg	−8.03, −1.81	1.92 × 10^−3^	0.004	27.82	92.6%	89%, 95.1%	1.82 × 10^−26^	0.15					
Height	2	146	141	−0.01	0.01	0.00	m	−0.02, 0.02	.79	0.84				.22						
Body mass index	18	722	809	−5.50	0.20	−2.35	kg/m^2^	−3.28, −1.42	6.79 × 10^−7^	2.21 × 10^−6^	3.81	97.6%	97.0%, 98.1%	3.96 × 10^−138^	0.04	−2.10	−2.53, −1.67	<.0001	5	18.5–24.9 kg/m^2^
Fat mass	15	589	617	−6.89	1.70	−2.37	kg	−3.75, −0.98	8.29 × 10^−4^	0.002	6.65	94.6%	92.5%, 96.1%	5.45 × 10^−47^	0.73					
Body fat percentage	14	530	617	−14.29	2.40	−3.15	%	−5.61, −0.70	.01	0.02	19.77	89.0%	83.3%, 92.7%	5.37 × 10^−19^	0.05	−2.50	−4.27, −0.73	.006	3	20%–25%
Trunk body fat percentage	6	179	224	−8.24	12.30	5.84	%	−0.14, 11.83	.06	0.09	53.72	94.4%	90.4%, 96.8%	7.59 × 10^−18^	0.03	11.99	9.54, 14.44	<.0001	52	
Extremity body fat percentage	5	129	124	−10.00	0.10	−6.37	%	−9.52, −3.23	7.24 × 10^−5^	1.82 × 10^−4^	10.48	78.1%	47.5%, 90.9%	.001	0.08					
Fat‐free mass	12	634	697	−4.80	0.20	−1.82	kg	−2.57, −1.08	1.72 × 10^−6^	5.41 × 10^−6^	0.98	60.2%	25.0%, 78.9%	.004	0.61					
Fat‐free mass percentage	6	239	299	−1.00	14.29	2.83	%	−1.89, 7.55	.24	0.32	33.35	93.5%	88.5%, 96.3%	3.89 × 10^−15^	0.08					
Trunk fat‐free mass percentage	3	124	133	−1.09	0.90	−0.40	%	−1.57, 0.77	.50	0.60	0.85	77.9%	28.8%, 93.2%	.01						
Extremity fat‐free mass percentage	3	124	133	−1.90	1.20	−0.30	%	−1.97, 1.37	.73	0.79	1.93	85.0%	55.7%, 94.9%	.001						
Bone mineral content (whole body)	2	174	194	−0.09	−0.09	−0.09	kg	−0.13, −0.05	5.75 × 10^−6^	1.63 × 10^−5^				1.00						
Bone mineral density (whole body)	2	74	67	−0.04	−0.01	−0.02	g/cm^2^	−0.04, 0.01	.20	0.27				.34						
Total body water	3	204	159	−6.66	−0.80	−3.71	L	−7.07, −0.36	.03	0.05	8.25	95.1%	88.9%, 97.8%	1.59 × 10^−9^						3.3–3.6 L
Fasting glucose	3	45	41	−8.83	5.41	−1.78	mg/dl	−8.98, 5.42	.63	0.73	28.75	66.9%	0.0%, 90.5%	.05						<140 mg/dl
Fasting insulin	3	46	74	−4.90	9.72	8.31	pmol/L	−2.26, 18.87	.12	0.17	0	0.0%	0.0%, 64.8%	.74						<173.6 pmol/L
Ghrelin	2	33	61	−89.00	105.00	7.34	pmol/L	−182.77, 197.45	.94	0.96	17,674.47	93.9%	80.6%, 98.1%	4.98 × 10^−5^						114.4–154 pmol/L
Leptin	5	72	113	−9.18	−0.40	−3.91	ng/ml	−7.37, −0.45	.03	0.05	12.07	81.3%	56.6%, 92%	2.59 × 10^−4^	0.17					3.3–18.3 ng/ml
Free triiodothyronine	2	33	63	−1.00	−0.90	−0.91	pmol/L	−1.36, −0.47	5.26 × 10^−5^	1.40 × 10^−4^				.88						3.5–9.5 pmol/L

Female		Number of participants				Meta‐analysis					Heterogeneity				Small study effects					
Pretreatment/posttreatment	*k*	AN	CO	Min	Max	MD	Unit	95% CI	*p*	*Q*	*τ* ^2^	*I* ^2^	95% CI	*p*	T&S	Copas	95% CI	*p*	*N* unpub	Reference values
Weight	12	398	436	4.30	16.00	9.93	kg	8.17, 11.68	1.44 × 10^−28^	2.11 × 10^−27^	7.54	80.0%	65.0%, 88.0%	1.35 × 10^−7^	0.32					
Height	2	146	146	0.00	0.01	0.00	m	−0.01, 0.02	.67	0.75				.53						
Body mass index	18	722	807	1.16	6.40	3.39	kg/m^2^	2.71, 4.08	4.19 × 10^−22^	4.10 × 10^−21^	1.99	95.0%	94.0%, 97.0%	8.54 × 10^−68^	0.37					18.5–24.9 kg/m^2^
Fat mass	15	589	636	−0.86	8.90	6.39	kg	5.13, 7.65	2.79 × 10^−23^	3.07 × 10^−22^	5.42	95.0%	93.0%, 96.0%	7.66 × 10^−49^	0.68					
Body fat percentage	14	530	530	3.85	15.70	10.41	%	7.96, 12.87	9.23 × 10^−17^	6.25 × 10^−16^	19.21	93.0%	90.0%, 95.0%	2.07 × 10^−32^	0.40					20%–25%
Trunk body fat percentage	6	179	179	−2.59	6.20	4.16	%	2.07, 6.25	9.67 × 10^−5^	2.30 × 10^−4^	4.08	59.0%	0.0%, 83.0%	3.25 × 10^−2^	0.08					
Extremity body fat percentage	5	129	129	−1.10	−0.43	−0.95	%	−2.61, 0.71	.26	0.34	0	0.0%	0.0%, 0.0%	1.00	0.83					
Fat‐free mass	12	634	719	0.00	6.90	2.98	kg	1.74, 4.22	2.35 × 10^−6^	6.89 × 10^−6^	3.87	93.0%	89.0%, 95.0%	4.57 × 10^−27^	0.66					
Fat‐free mass percentage	6	239	239	−13.92	0.10	−9.00	%	−13.63, −4.37	1.38 × 10^−4^	3.11 × 10^−4^	31.68	99.0%	99.0%, 99.0%	9.94 × 10^−112^	0.49					
Trunk fat‐free mass percentage	3	124	124	−0.73	1.32	0.17	%	−1.06, 1.40	.79	0.84	0.78	72.3%	6.5%, 91.8%	.03						
Extremity fat‐free mass percentage	3	124	124	−0.90	3.04	1.01	%	−1.08, 3.10	.34	0.43	2.83	84.6%	54.2%, 94.8%	.002						
Bone mineral content (whole body)	2	174	174	0.05	0.07	0.05	kg	0.02, 0.09	.006	0.01				.74						
Bone mineral density (whole body)	2	74	74	−0.01	0.01	0.01	g/cm^2^	−0.02, 0.03	.64	0.73				.53						
Total body water	3	204	251	0.16	1.80	0.58	L	−0.24, 1.40	.17	0.23	0.18	30.8%	0.0%, 92.8%	.24						3.3–3.6 L
Fasting glucose	3	45	45	5.23	16.22	9.51	mg/dl	2.68, 16.35	.006	0.01	23.25	66.3%	0.0%, 90.3%	.05						<140 mg/dl
Fasting insulin	3	46	84	9.03	38.19	15.92	pmol/L	1.89, 29.95	.03	0.05	42.79	24.2%	0.0%, 92.1%	.27						<173.6 pmol/L
Ghrelin	2	33	71	−112.50	−104.00	−107.76	pmol/L	−161.47, −54.05	8.41 × 10^−5^	2.06 × 10^−4^				.88						114.4–154 pmol/L
Leptin	5	72	110	1.10	5.50	2.83	ng/ml	1.22, 4.44	5.79 × 10^−4^	0.001	2.41	71.0%	26.0%, 89.0%	.008	0.42					3.3–18.3 ng/ml
Free triiodothyronine	2	33	71	0.80	0.80	0.80	pmol/L	0.39, 1.21	1.33 × 10^−4^	3.08 × 10^−4^				1.00						3.5–9.5 pmol/L

Female		Number of participants				Meta‐analysis					Heterogeneity				Small study effects					
Weight‐recovered	*k*	AN	CO	Min	Max	MD	Unit	95% CI	*p*	*Q*	*τ* ^2^	*I* ^2^	95% CI	*p*	T&S	Copas	95% CI	*p*	*N* unpub	Reference values
Weight	11	305	530	−8.70	1.65	−1.83	kg	−3.68, 0.02	.05	0.08	5.80	66.4%	36.4%, 82.2%	.001	0.95					
Height	6	174	341	−0.03	−0.01	−0.01	m	−0.03, 0.00	.02	0.03	0.00	0.0%	0.0%, 0.0%	.94	0.51					
Body mass index	12	398	660	−2.80	0.50	−0.68	kg/m^2^	−1.38, 0.02	.06	0.09	1.17	93.3%	90.2%, 95.5%	1.16 × 10^−29^	0.84					18.5–24.9 kg/m^2^
Fat mass	12	323	563	−6.30	1.70	−0.76	kg	−1.75, 0.22	.13	0.18	1.82	63.0%	31.0%, 80.1%	.002	0.47					
Body fat percentage	10	392	463	−2.32	2.40	−0.03	%	−1.09, 1.02	.95	0.96	1.06	39.0%	0.0%, 70.9%	.10	0.44					14%–25%
Visceral adipose tissue	2	37	18	0.10	0.24	0.19	kg	0.03, 0.35	.02	0.03				.42						
Subcutaneous adipose tissue	2	37	18	−0.52	−0.42	−0.50	kg	−1.81, 0.82	.46	0.56				.95						
Trunk body fat percentage	5	147	207	−3.30	12.30	5.86	%	−0.83, 12.54	.09	0.13	55.17	96.3%	93.7%, 97.8%	1.75 × 10^−22^	0.28					
Extremity body fat percentage	3	81	89	−10.00	20.09	0.53	%	−18.65, 19.71	.96	0.96	285.64	99.4%	99.1%, 99.6%	7.45 × 10^−72^						
Fat‐free mass	9	381	645	−2.10	−0.30	−1.27	kg	−1.79, −0.75	1.81 × 10^−6^	5.49 × 10^−6^	0.03	0.0%	0.0%, 39.7%	.79	0.54					
Fat‐free mass percentage	4	206	256	−1.00	0.00	0.00	%	−0.06, 0.06	.95	0.96	0.00	0.0%	0.0%, 70.9%	.66	0.35					
Trunk fat‐free mass percentage	2	105	113	−1.11	−0.88	−0.91	%	−1.33, −0.49	2.29 × 10^−5^	6.30 × 10^−5^				.72						
Extremity fat‐free mass percentage	2	105	113	−0.27	1.59	0.56	%	−1.25, 2.37	.54	0.64	1.49	86.2%	45.0%, 96.5%	.01						
Bone mineral content (whole body)	2	134	161	−0.23	−0.09	−0.10	kg	−0.18, −0.03	.008	0.01	0.001	12.9%	NA%, NA%	.28						
Bone mineral density (lumbar spine)	2	31	210	−0.09	−0.04	−0.08	g/cm^2^	−0.12, −0.04	6.28 × 10^−5^	1.63 × 10^−4^				.39						
Bone mineral density (femoral neck)	2	31	210	−0.11	−0.05	−0.10	g/cm^2^	−0.15, −0.04	5.31 × 10^−4^	0.001				.40						
Leptin	2	13	30	−1.30	−0.40	−0.43	ng/ml	−2.41, 1.55	.67	0.75				.87						3.3–18.3 ng/ml

To correct for multiple comparison, we calculated FDR‐adjusted *Q* values. To test for small study effects or publication bias, we performed a Thompson and Sharp (T&S) test. A *p* value below .05 indicated small study effects or publication bias. In this case, a Copas model was fitted to adjust the original meta‐analysis (Agüera et al., 2015; Bachmann et al., 2014; Benninghoven, Raykowski, Solzbacher, Kunzendorf, & Jantschek, 2007; Bratland‐Sanda et al., 2010; Bredella et al., 2012, 2008; Chudecka & Lubkowska, 2016; de Alvaro et al., 2007; Dellava et al., 2009; Delporte et al., 2003; de Mateo Silleras et al., 2013; Diamanti et al., 2007; DiVasta et al., 2007, 2011; Dostálová et al., 2009; El Ghoch et al., 2012, 2015; El Ghoch, Calugi, et al., 2017; El Ghoch, Milanese, et al., 2014; El Ghoch, Pourhassan, et al., 2017; Estour et al., 2017; Faje et al., 2014; Fazeli et al., 2010; Fernández‐Soto et al., 2013; Galusca et al., 2015; Germain et al., 2010, 2007, 2016; Gniuli et al., 2001; Grinspoon et al., 1996, 2001; Guo et al., 2013; Haas et al., 2018, 2005; Iacopino et al., 2003; Karczewska‐Kupczewska et al., 2010; Karlsson et al., 2000; Kaválková et al., 2012; Kerruish et al., 2002; Kirchengast & Huber, 2004; Konstantynowicz et al., 2011; Kosmiski et al., 2014; Maïmoun et al., 2018; Marra et al., 2019; Mayer et al., 2009, 2005; Mika, Herpertz‐Dahlmann, Heer, & Holtkamp, 2004; Misra et al., 2013; Moreno, Djeddi, & Jaffrin, 2008; Mörkl et al., 2017; Nakahara et al., 2007; Nakai et al., 1999; Prioletta et al., 2011; Rigaud et al., 2010; Scalfi, Marra, Caldara, Silvestri, & Contaldo, 1999; Scalfi et al., 2002; Schneider et al., 1998; Schorr et al., 2019; Singhal et al., 2018; Tagami et al., 2004; Tanaka et al., 2003; Tonhajzerova et al., 2019; Weinbrenner et al., 2004; Wu et al., 2019).

Abbreviations: 95% CI, 95% confidence interval; AN, anorexia nervosa; CO, controls; Copas, Copas model; *k*, number of studies; MD, mean difference; T&S, Thompson & Sharp; *N* unpub, number of potentially unpublished studies.

### 3.4 Primary outcomes: Body composition

#### 3.4.1 Anthropometrics

On average, pretreatment female AN cases had a 15.64 kg (95% CI: −16.98, −14.30, *Q* = 5.59 × 10^−114^) lower body weight and were 0.01 m (95% CI: −0.02, 0.00, *Q* = 0.02) shorter than healthy controls (Table S4). After treatment, female AN patients still weighed 4.92 kg (95% CI: −8.03, −1.81, *Q* = 1.92 × 10^−3^) less than healthy controls. Before treatment, male AN cases weighed 15.48 kg (95% CI: −22.42, −8.54, *Q* = 1.80 × 10^−5^) less than healthy controls and showed no differences in height compared with controls.

Correspondingly, the pretreatment BMI difference between female AN cases and controls was −5.81 kg/m^2^ (95% CI: −6.25, −5.38, *Q* = 2.83 × 10^−152^), which reduced to −2.10 kg/m^2^ (95% CI: −2.53, −1.67, *p*_*adjCopas*_ < .0001) after treatment as most patients gained on average 9.93 kg (95% CI: 8.17, 11.68, *Q* = 2.11 × 10^−27^) during treatment. Posttreatment BMI in females was primarily accounted for by gains in fat mass (*β*_*metareg*_ = 0.81, *p* = 7.03 × 10^−7^) but not through fat‐free mass (Table S5). After weight recovery, no statistically significant MD in BMI between female AN cases and controls was detected. The pretreatment BMI difference between male AN cases and controls was −5.48 kg/m^2^ (95% CI: −7.87, −3.09, *Q* = 1.80 × 10^−5^).

#### 3.4.2 Fat mass

**Figure 3:**
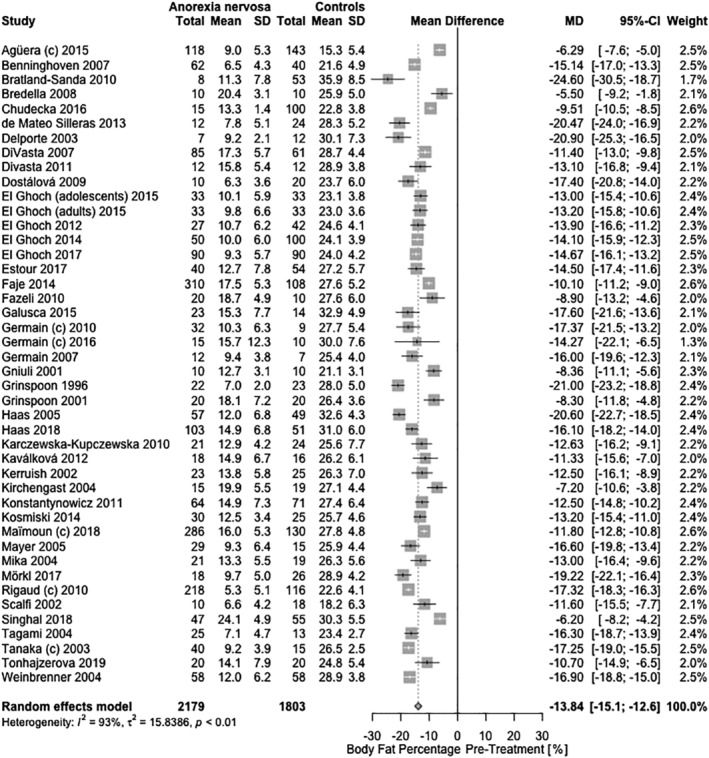
Cross‐sectional meta‐analysis of studies reporting body fat percentage in acutely‐ill/pretreatment female AN patients compared with healthy controls. Forty‐four samples had the appropriate data for the meta‐analysis with 2,179 AN cases and 1,803 controls. A random‐effects meta‐analysis revealed a pooled estimate of the mean difference (MD: −13.8%; 95% CI: −15.1, −12.6; *Q* = 5.49 × 10^−101^) with the mean differences ranging from −24.6% to −5.5%. Heterogeneity between studies was statistically significant (*τ* ^*2*^ = 15.84; *p* = 1.40 × 10^−98^; *I* ^2^ = 92.8%). C, subtype‐combined sample

The pretreatment body composition of individuals with AN was significantly altered. Compared with healthy controls, female AN cases had 8.80 kg (95% CI: −9.81, −7.79, *Q* = 1.01 × 10^−63^) lower fat mass, corresponding to a 13.9% (95% CI: −15.1, −12.6, *Q* = 5.49 × 10^−101^; Figure 3) lower total body mass. Male AN cases had 5.87 kg (95% CI: −8.98, −2.75, *Q* = 2.70 × 10^−4^) lower fat mass, corresponding to 7.5% (95% CI: −10.8, −4.2, *Q* = 1.8 × 10^−5^) lower total body mass. This suggests that body fat was on average 50% lower than in healthy controls. Body fat percentage (*β*_*metareg*_ = −134.53, *p* = 0.01) and absolute fat mass (*β*_*metareg*_ = −35.50, *p* = 2.03 × 10^−5^) were associated with whole‐body bone mineral density of female AN patients. Absolute fat mass was also associated with mean age at diagnosis (*β*_*metareg*_ = −1.21, *p* = 2.42 × 10^−4^) in females (Table S4).

After treatment, female AN patients had a 2.37 kg (95% CI: −3.75, −0.98, *Q* = 0.002) lower fat mass, which corresponded to 2.5% (95% CI: −4.3, −0.7, *p*_*adjCopas*_ = 0.006) less total body mass compared with healthy controls. Female AN patients gained 6.39 kg (95% CI: 5.13, 7.65, *Q* = 3.07 × 10^−22^) fat mass following treatment, which corresponded to 10.4% (95% CI: 7.96, 12.87, *Q* = 6.25 × 10^−16^) of total body mass. Posttreatment fat mass (*β*_*metareg*_ = −0.23, *p* = .01; Table S5) and gain in fat mass during treatment (*β*_*metareg*_ = −0.20, *p* = .02; Table S6) were negatively associated with the presence of amenorrhea. Following weight recovery, these values fully returned to levels seen in female healthy controls.

Specifically, compared with healthy controls, female AN patients had 3.51 kg (95% CI: −4.58, −2.43, *Q* = 8.07 × 10^−10^) less trunk fat mass prior to treatment. In relative terms, however, female AN patients had lower extremity body fat with 5.4% (95% CI: −8.4, −2.4, *Q* = 8.23 × 10^−4^) less total body mass. The presence of amenorrhea was significantly associated with lower extremity fat mass (*β*_*metareg*_ = 0.31, *p* = .04; Table S4).

After treatment, female AN patients showed a higher trunk body fat percentage than controls at 12.0% (95% CI: 9.5, 14.4, *p*_*adjCopas*_ < 1.00 × 10^−4^) of total body mass. However, this finding was strongly influenced by publication bias with an estimated 52 unpublished studies. These results on body composition were not influenced by height as female and male cases and controls showed no meaningful difference (i.e., 1 cm pretreatment) or by age as meta‐regressions were nonsignificant (Tables S4–S7).

#### 3.4.3 Fat‐free mass

**Figure 4:**
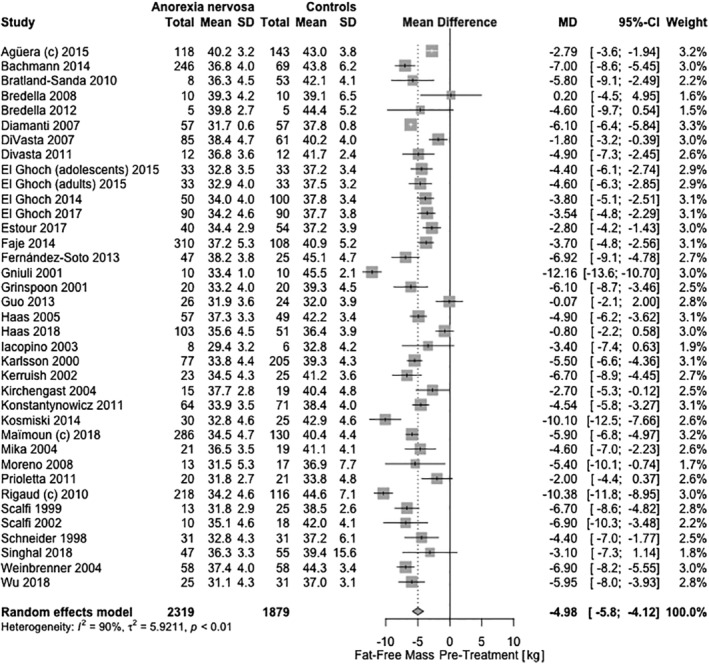
Cross‐sectional meta‐analysis of studies reporting fat‐free mass content in acutely‐ill/pretreatment female AN patients compared with healthy controls. Thirty‐seven samples had the appropriate data for the meta‐analysis with 2,319 AN cases and 1,879 controls. A random‐effects meta‐analysis revealed a pooled estimate of the mean difference (MD: −4.98 kg; 95% CI: −5.8, −4.1; *Q* = 1.99 × 10^−28^) with the mean differences ranging from −12.16 to 0.20 kg. Heterogeneity between studies was statistically significant (*τ* ^*2*^ = 5.92; *p* = 1.22 × 10^−58^; *I* ^2^ = 90.5%). C, subtype‐combined sample

Overall, the fat‐free mass in female AN patients was 4.98 kg (95% CI: −5.85, −4.12, *Q* = 1.99 × 10^–^^28^; Figure 4) lower before treatment than in controls, corresponding to 12.3% (95% CI: 8.1, 16.5, *Q* = 3.21 × 10^−8^) higher proportion of total body mass. In males, fat‐free mass in AN patients was −9.37 kg (95% CI: −12.47, −6.27, *Q* = 2.00 × 10^−8^) lower before treatment than in controls. During treatment, female AN patients gained 2.98 kg (95% CI: 1.74, 4.22, *Q* = 6.89 × 10^−6^) fat‐free mass, resulting in 1.82 kg (95% CI: −2.57, −1.08, *Q* = 5.41 × 10^−6^) lower fat‐free mass compared to controls. Yet, weight‐recovered female individuals with AN still showed 1.27 kg (95% CI: −1.79, −0.75, *Q* = 5.49 × 10^−6^) lower fat‐free mass than controls.

More specifically, pretreatment fat‐free mass of the extremities in females was 1.5% (95% CI: −2.0, −1.0, *Q* = 8.84 × 10^−9^) less of total body mass. After treatment, no marked regional differences in fat‐free mass were observed in female AN patients. However, weight‐recovered female individuals with AN had 0.9% (95% CI: −1.3, −0.5, *Q* = 6.30 × 10^−5^) lower trunk fat‐free mass of total body mass than controls.

Before treatment, we observed a 393.95 kcal/day (95% CI: −531.04, −256.86, *Q* = 6.53 × 10^−8^) lower resting energy expenditure and 4.77 L (95% CI: −6.13, −3.41, *Q* = 3.06 × 10^−11^) less total body water in female AN patients, which persisted with 3.71 L (95% CI: −7.07, −0.36, *Q* = 0.05) after treatment. Both were measured by BIA. However, resting energy expenditure was not corrected for fat‐free mass or body mass in the original studies, limiting its interpretability. Before treatment, total body water in females was associated with fat mass (*β*_*metareg*_ = 0.60, *p* = .01) and the difference in fat‐free mass between AN cases and controls (*β*_*metareg*_ = 0.48, *p* = .003).

Before treatment, only the amount of total body water was significantly different between female individuals (*p*_*subgroup*_ = 1.47 × 10^−4^) suffering from the restricting (−5.31 L, 95% CI: −8.15, −2.47, *p*_*R*_ = 2.47 × 10^−4^, *k* = 4) or the binge‐eating/purging subtype (−11.1 L, 95% CI: −12.04, −10.16, *p*_*BP*_ = 5.06 × 10^−119^, *k* = 1; Table S3). However, this finding was limited by only one study investigating the binge‐eating/purging subtype).

### 3.5 Secondary outcome: Bone mineral measures

#### 3.5.1 Bone mineral content and density

Compared with healthy controls, whole‐body bone mineral content in female individuals with AN was 0.16 kg (95% CI: −0.19, −0.12, *Q* = 2.73 × 10^−20^) lower before treatment and 0.09 kg (95% CI: −0.13, −0.05, *Q* = 1.63 × 10^−5^) lower after treatment. Weight‐restored female individuals with AN showed 0.10 kg (95% CI: −0.18, −0.03, *Q* = 0.01) lower whole‐body bone mineral content compared to controls as they gained on average 0.05 kg (95% CI: 0.02, 0.09, *Q* = 0.01) during treatment. The interpretability of these estimates is limited due to the insufficient follow‐up time after weight recovery, exceeding 6 months in only two studies (Dellava et al., 2009; Karlsson et al., 2000). The pretreatment whole‐body bone mineral content in females was associated with fat‐free mass (*β*_*metareg*_ = 0.02, *p* = .02) and fat mass (*β*_*metareg*_ = 0.05, *p* = .02), as well as the difference in fat mass between AN patients and controls (*β*_*metareg*_ = 0.04, *p* = .002; Table S4). Accordingly, pretreatment whole‐body bone mineral density was 0.03 g/cm^2^ (95% CI: −0.06, −0.01, *p*_*adjCopas*_ = .02) lower in females with AN, but our analysis showed a density comparable with healthy controls posttreatment. However, only two studies with 74 AN cases could be included in this analysis.

Before treatment, female AN patients exhibited lower bone mineral density in several regions, including hip, lumbar spine, and femoral neck, with a few being likely to persist after weight recovery. These findings were associated with duration of illness, the age of AN cases, and differences in fat mass between cases and controls (Supporting Information: Secondary Outcomes: Detailed Bone Mineral Measures and Table S4). Cases and controls in our meta‐analyses were age‐ and height‐matched (Figures S1 and S6); therefore, these variables are unlikely to be associated with these results.

#### 3.5.2 Secondary outcomes: Metabolites and hormones

Exploratory results showed that fasting insulin and glucose concentrations were lower in female AN patients compared with controls but not associated with fat or fat‐free mass, while lower leptin was associated with fat mass pretreatment. After treatment, these measures returned to concentrations seen in healthy controls. Before treatment, thyroid hormones, cortisol, and IGF‐1 were lower in female AN patients, and all three measures were associated with fat mass, whereas higher cortisol in AN patients was associated with fat‐free mass. For detailed results, see Supporting Information: Secondary Outcomes: Metabolites and Hormones.

#### 3.5.3 Methodological moderators

We observed strong between‐study heterogeneity (Table 1). To investigate how differences in study design, samples, and measurement methods may influence the primary and secondary outcomes, we performed an additional set of meta‐regressions (Tables S4–S7). The method of body composition measurement was associated with pretreatment body fat percentage (*β*_*DXA*_ = 3.05, *p* = .01), fat‐free mass (*β*_*Isotope Dilution*_ = −6.18, *p* = .008), and fat‐free mass percentage (*β*_*DXA*_ = −8.28, *p* = .01), and posttreatment body fat percentage (*β*_*DXA*_ = 6.39, *p* = .005) in females. Furthermore, femoral neck bone mineral density (*β*_*Outpatient*_ = −0.12, *p* = 7.65 × 10^−4^) significantly differed between female inpatients and outpatients.

## 4 DISCUSSION

Our primary meta‐analyses showed marked alterations in body composition traits in patients with AN before and after treatment. Before treatment, all three major body compartments—fat, fat‐free, and bone mass—showed significant reductions that were only partially restored after treatment. Our meta‐analysis estimated ~50% lower body fat in AN patients which was mirrored by leptin concentrations (Perry & Shulman, 2018), both of which recovered with treatment. In females, significant differences were observed in body fat distribution after treatment as body fat is primarily stored in the trunk. This distribution pattern may be due to increased insulin sensitivity observed in AN patients (Ilyas et al., 2018) potentially similar to observations in healthy individuals after short‐term overfeeding (McLaughlin et al., 2016). We did not detect meaningful or statistically significant differences in body fat distribution in weight‐restored patients, indicating potential redistribution occurring over longer term follow‐up.

A new finding from our meta‐analysis is that lower fat mass in females with AN was correlated with significantly low bone mineral content and density across the whole body. We speculate that the hormonal cross‐talk between fat and bone tissue may be influencing this association (El Ghoch et al., 2016; Hawkes & Mostoufi‐Moab, 2018), potentially mediated through greater bone marrow adipose tissue observed in AN (Fazeli & Klibanski, 2018; Suchacki & Cawthorn, 2018). Whole‐body bone mineral content remained low in weight‐recovered individuals with AN. However, as only two studies followed patients for longer than 6 months (Dellava et al., 2009; Karlsson et al., 2000), the duration of follow‐up was insufficient to draw firm conclusions because bone mineral mass is slow to normalize. Future studies should be designed to capture long‐term changes. In men with AN, fat mass and fat‐free mass were lower before treatment than in controls. However, long‐term follow‐up studies are missing. It has been reported that short‐term weight restoration may normalize body composition patterns but could also lead to central adiposity (El Ghoch, Calugi, et al., 2017), but sample sizes of reports of males are very small. Additionally, in our analysis alterations in bone mineral mass did not affect the height of individuals with AN.

Another new finding in our meta‐analysis is that we observed a 5 kg lower fat‐free mass in female AN patients, which remained lower even after treatment and in weight‐recovered AN patients, indicating that current treatment regimens may insufficiently target fat‐free mass. Future studies should also assess muscle mass to identify the components of fat‐free mass that are most associated with this reduction.

Our secondary outcomes—associations between detailed body composition and laboratory parameters in AN—were difficult to assess as only a few published studies reported both outcomes consistently. Most biochemical alterations were within the range of normal reference values. However, serious alterations can occur in certain individuals with AN that warrant vigilance by clinicians.

Pretreatment fasting insulin and glucose were reduced in AN patients independent of fat mass, but both concentrations normalized following treatment, suggesting that increased insulin sensitivity (Ilyas et al., 2018) may be a temporary state in AN. The relationship between AN and insulin sensitivity should be investigated by euglycemic hyperinsulinemic clamp that showed mixed findings in very small samples (Castillo, Scheen, Jandrain, & Lefèbvre, 1994; Castillo, Scheen, Lefebvre, & Luyckx, 1985; Dostálová et al., 2009; Karczewska‐Kupczewska et al., 2010; Pannacciulli et al., 2003; Prioletta et al., 2011; Zuniga‐Guajardo, Garfinkel, & Zinman, 1986). This approach is supported by epidemiological associations of AN with type 1 diabetes (Hedman et al., 2018) and its genetic overlap with fasting insulin (Duncan et al., 2017), type 2 diabetes (Watson et al., 2019), and insulin sensitivity (Hübel et al., 2018).

AN was associated with body fat percentage‐associated low T_3_‐ and T_4_‐syndrome pretreatment, whereas thyroid‐stimulating hormone concentrations were normal. Associations between fat mass and thyroid hormones have been described before (Kwon et al., 2018); however, sufficiently powered long‐term follow‐up studies in AN are absent.

Steroid hormone concentrations were altered showing high cortisol, low estradiol, and normal testosterone. Estradiol was negatively associated with fat‐free mass, whereas cortisol was positively associated with fat mass. These findings suggest that fat‐free mass may be a potential moderator for the return of menses in AN patients and should be further investigated as most research in recovery of menses primarily focused on BMI‐ or weight‐related cutoffs (Misra et al., 2006; Swenne, 2004). Potential reverse causation should also be taken into account where altered estradiol concentrations may precede changes in fat‐free mass.

Overall, the meta‐analyzed study sample was highly selected and biased as it comprised mostly European females aged between 14 and 31 years, emphasizing the urgent need for studies including diverse ancestries, such as Asia, South and Central America, and Africa. Females and males differ in body composition and metabolic characteristics (Karastergiou & Fried, 2017; Link & Reue, 2017), underscoring the need for more studies on males with AN. Our study selection was limited by the lack of control groups and underreported extensive sample overlap. Moreover, control groups were only measured at baseline in all longitudinal studies, failing to account for age‐ and growth‐related variation, potentially inflating estimates. Furthermore, no clear‐cut and consistent definition of recovery from AN was used across studies, contributing to heterogeneity (Murray, Loeb, & Le Grange, 2018). This underscores the necessity of developing standard definitions of remission and recovery in the eating disorders field (Bardone‐Cone, Hunt, & Watson, 2018).

Methodologically, we observed effects of either BIA or DXA on the measurement of body composition in AN, questioning the comparability of the two methods. Larger, longitudinal validation studies comparing both methods with whole‐body MRI in eating disorders should be conducted. Additional factors influencing body composition and biochemical measures, such as menstrual cycle, diurnal changes, fasting, and preanalytical procedures are summarized in Table 3 and should be carefully assessed in future studies (Hernandes et al., 2017).

**Table 3:** Minimum requirement of variables that should be assessed, reported, and included in statistical analyses of case–control studies examining anorexia nervosa or other eating disorders to facilitate reproducibility, meta‐analysis, and meta‐regression

**Sampling**	**Sample characteristics**
Cases and controls Underlying population: community, hospital Consecutive sample or selection If consecutive, attrition and reasons Diagnosis and ascertainment Diagnostic schema Independent validation Controls Repeated measurement at follow‐up Exclusion of current and history of diagnosis (i.e., screening) Matching (e.g., age, sex) Exclusion criteria	Cases and controls Age Biological sex and gender Height Weight Body mass index Ancestry Socioeconomic status & education Cases Age of onset Duration of illness
**Body composition**	**Menstrual status**
Fat mass Fat‐free mass Bone mineral content and density Ideally: Muscle mass Measurement method: e.g., MRI, DXA, or BIA Physical activity (ideally accelerometer data)	Cases Dysmenorrhea or amenorrhea Duration of amenorrhea Age of menarche If menstruating, stage or day of cycle Controls Stage or day of the menstrual cycle (e.g., follicular phase)
**Blood sampling**	**Substances**
Blood sample type whole blood, serum, plasma Fasting state Fasting period The time point of blood sampling Pre‐analytics Storage Storage duration	Dose and duration of Contraceptives Supplements & vitamins Medication Prescription Over the counter Laxatives Illicit drugs Alcohol consumption Smoking behavior

a Adapted from Hernandes, Barbas, & Dudzik, 2017.

Abbreviations: BIA, bioelectrical impedance analysis; DXA, dual‐energy X‐ray absorptiometry; MRI, magnetic resonance imaging.

Most importantly, blood comprises approximately 3,500 highly correlated and interacting proteins (http://hupo.org; Schwenk et al., 2017) and 4,600 metabolites (serummetabolome.ca; Psychogios et al., 2011); therefore, the measurement of single proteins, hormones, or metabolites is ill‐advised. Metabolomics, proteomics, and lipidomics can capture large amounts of information at adequate statistical power when used in large samples (Hernandes et al., 2017). Additionally, large epidemiological databases that have measured biomarkers in childhood, such as the Avon Longitudinal Study of Parents and Children (ALSPAC; Golding, Pembrey, Jones, & ALSPAC Study Team, 2001) and Generation R (Kooijman et al., 2016), should be harnessed to determine whether those who go on to develop AN show evidence for premorbid differences in body composition and biochemical parameters as has been observed for BMI by Yilmaz et al. (2019).

## 5 CONCLUSIONS

Detailed measurement of body composition with simple methods, such as BIA or DXA, which offers additional information on bone tissue, may help refine our understanding of the nature of AN and its diagnosis. Our meta‐analyses showed that all body compartments were markedly altered in AN. Individuals with AN presented with 50% lower fat mass and prolonged recovery periods for fat‐free mass and bone mineral content. The core implication of body composition differences are alterations in metabolism, growth, and development. Although results must be interpreted with caution given small samples, we found evidence indicating alterations in fasting insulin, thyroid, sex, and stress hormones in AN, which appeared to partially normalize with weight gain and recovery. Large birth cohorts that collected information on eating disorders along with metabolomic information offer a rich and exciting opportunity for prospective investigations that add to our understanding of body composition and metabolic mechanisms in risk and maintenance of eating disorders.

## CONFLICT OF INTEREST

C.M.B. reports Shire (Scientific Advisory Board member) and Pearson (author, royalty recipient) (unrelated to the content of this manuscript). G.B. has received grant funding from and served as a consultant to Eli Lilly and has received honoraria from Illumina and has served on advisory boards for Otsuka (all unrelated to the content of this manuscript). The remaining authors declare no potential conflict of interest.

## AUTHOR CONTRIBUTIONS

C.H., Z.Y., C.M.B., and G.B. designed research; C.H., Z.Y., K.S., L.B., A.H., J.G.G. conducted research; C.H., Z.Y., K.S., L.B., A.H., J.G.G., E.H. provided essential materials; C.H. analyzed data or performed statistical analysis; C.H., Z.Y., L.B., K.S., E.H., G.B., C.M.B. wrote paper; C.H. had primary responsibility for final content. All authors read and approved the final manuscript.

## DATA AVAILABILITY

All data and all scripts used for data analysis are available on http://github.com/topherhuebel/metabcan.

## Supporting information


**Appendix S1**. Supporting Information.

**Appendix S2**. Supporting Information.
