# Supplementary material for: Body composition in anorexia nervosa: Meta‐analysis and meta‐regression of cross‐sectional and longitudinal studies
Source: Int J Eat Disord. 2019 Sep 12;52(11):1205–23. doi: 10.1002/eat.23158 (PMC6899925; doi:10.1002/eat.23158)
Supplement: Supplementary file 1 — Appendix S1. Supporting Information. [file EAT-52-1205-s001.docx]

**Body composition in anorexia nervosa: meta-analysis and meta-regression of cross-sectional and longitudinal studies**

Christopher Hübel, Zeynep Yilmaz, Katherine Schaumberg, Lauren Breithaupt, Avina Hunjan, Eleanor Horne, Judit García-González, Paul F. O’Reilly, Cynthia M. Bulik, Gerome Breen

**Online Supporting Information**

**Secondary outcomes: bone mineral measures**

1) Results

**Secondary outcomes: metabolites and hormones**

2) Results

**Meta-analyses: forest plots**

3) Meta-analyses of study sample characteristics comparing anorexia nervosa patients with healthy controls

4) Q value plot of meta-analyses uncapped

5) Cross-sectional meta-analyses of studies comparing acutely-ill/pre-treatment anorexia nervosa patients with healthy controls

6) Cross-sectional meta-analyses of studies comparing post-treatment anorexia nervosa patients with healthy controls

7) Longitudinal meta-analyses of studies comparing anorexia nervosa patients pre- and post-treatment

8) Cross-sectional meta-analyses of studies comparing weight-recovered anorexia nervosa patients with healthy controls

**Adjustment for small study effects**

9) Copas selection models

**Secondary outcomes: detailed bone mineral measures**

**1) Results**

Before treatment, anorexia nervosa (AN) patients exhibited lower bone mineral density in every region compared to controls: lumbar spine (-0.14 g/cm^2^, CI 95%: -0.18, -0.10, *Q* = 2.32 x 10^-12^), femoral neck (-0.14 g/cm^2^, CI 95%: -0.18, -0.09, *Q* = 1.93 x 10^-9^), and hip (-0.13 g/cm^2^, CI 95%: -0.15, -0.11, *Q* = 6.20 x 10^-45^) that persisted at the lumbar spine (-0.08 g/cm^2^, CI 95%: -0.12, -0.04, *Q* = 1.63 x 10^-4^) and femoral neck (-0.10 g/cm^2^, CI 95%: -0.15, -0.04, *Q* = 0.001) even after weight recovery. Again, the post-treatment estimates have limited validity as they were based on only two studies with 31 AN cases and insufficient follow-up duration. The meta-regressions showed that pre-treatment bone mineral densities were associated with duration of illness (hip; *β_metareg_* = -0.001 months, *p* = 0.05; lumbar spine; *β_metareg_* = -0.001 months, *p* = 0.02), age of AN cases (hip; *β_metareg_* = -0.005 years, *p* = 0.03; lumbar spine; *β_metareg_* = -0.01 years, *p* = 0.003), and the differences in fat mass (femoral neck; *β_metareg_* = 0.01 kg, *p* = 0.02; lumbar spine; *β_metareg_* = 0.01 kg, *p* = 0.04) and body fat percentage (lumbar spine; *β_metareg_* = 0.01, *p* = 0.001) between cases and controls (Supporting Information Table S4). Cases and controls in our meta-analyses were age- and height-matched (Supporting Information Figure S2 & S6), therefore, these variables cannot be associated with the mean difference between cases and controls.

**Secondary outcomes: metabolites and hormones**

**2) Results**

**Glucose homeostasis, gastrointestinal hormone, and adipokines**

Pretreatment fasting glucose (-7.01 mg/dL, CI 95%: -9.61; -4.40, *p_Copas_* < 1.00 x 10^-4^) after adjustment for publication bias and insulin (-19.23 pmol/L, CI 95%: -31.68, -6.77, *Q* = 0.005) were lower in AN than controls, but reached concentrations of healthy controls after treatment increasing by 9.51 mg/dL (CI 95%: 2.68, 16.35, *Q* = 0.01) and 15.92 pmol/L (CI 95%: 1.89, 29.95, *Q* = 0.05) during treatment. Neither fasting glucose nor fasting insulin were associated with body composition in AN patients before treatment (Supporting Information Table S4). Ghrelin was 149.2 pmol/L (CI 95%: 54.59, 243.81, *Q* = 0.004) higher than controls before treatment, decreased by 107.76 pmol/L (CI 95%: -161.47, -54.05, *Q* = 2.06 x 10^-4^) during treatment, and reached comparable concentrations of healthy controls after treatment. No differences in adiponectin were observed between pre-treatment comparing AN cases and healthy controls. After adjustment for publication bias, pre-treatment leptin was 7.20 ng/mL (CI 95%: -8.44, -5.96, *p_Copas_* < 1.00 x 10^-4^) lower than control values and associated with the difference in fat mass between AN patients and healthy controls (*β* = 0.80, *p* = 0.003) as indicated by meta-regression (Supporting Information Table S4). Leptin increased by 2.83 ng/mL (CI 95%: 1.22, 4.44, *Q* = 0.001) across treatment and showed a mean difference of -3.91 ng/mL (CI 95%: -7.37, -0.45, *Q* = 0.05) after treatment compared to controls. In weight-recovered individuals with AN, leptin concentrations were within the range of healthy controls. Although our analyses were meta-analytic, sample sizes of most comparisons regarding secondary outcomes were still small (Table 1).

**Thyroid, adrenal, growth, and sex hormones**

On average and before treatment, AN patients presented with lower fT_3_ (-1.32 pmol/L, CI 95%: -1.64, -1.00, *Q* = 6.85 x 10^-15^) and fT_4_ (-2.60 pmol/L, CI 95%: -3.26, -1.93, *Q* = 1.23x 10^-13^), but their mean TSH did not differ significantly from healthy controls. Pre-treatment fT_3_ was associated with body fat percentage in AN patients (*β* = -0.14, *p* = 0.05) and with the difference in fat mass between AN patients and controls (*β* = 0.14, *p* = 0.001) as indicated by meta-regression (Supporting Information Table S4). During treatment, fT_3_ increased by 0.80 pmol/L (CI 95%: 0.39, 1.21, *Q* = 3.08 x 10^-4^). However, post-treatment concentrations still differed by 0.91 pmol/L (CI 95%: -1.36, -0.47, *Q* = 1.40 x 10^-4^) between AN cases and controls. This finding was limited by an extremely small sample size (*n_AN_* = 33).

In comparison with controls, the mean pre-treatment cortisol was 131.92 nmol/L (CI 95%: 86.26, 177.58, *Q* = 5.46 x 10^-8^) higher in AN patients and showed an association with fat mass (*β* = 87.13, *p* = 0.04) as indicated by meta-regression. Estradiol was significantly lower (-40.83 pg/mL, CI 95%: -55.43, -26.23, *Q* = 1.49 x 10^-7^) in AN patients and associated with fat-free mass (*β* = -9.71, *p* = 2.56 x 10^-11^) and duration of illness (*β* = 0.54, *p* = 0.009) as indicated by meta-regression. Estradiol was also associated with the difference in fat-free mass (*β* = 8.69, *p* = 0.02) between AN patients and healthy controls (Supporting Information Table S4). In contrast, testosterone concentrations showed no difference between cases and controls. IGF-1 in AN cases was significantly lower by 95.86 µg/L (CI 95%: -117.93, -73.8, *Q* = 1.22 x 10^-16^) than healthy controls before treatment. IGF-1 was positively associated with age (*β* = 14.95, *p* = 7.31 x 10^-7^) and the difference in body fat percentage between cases and controls (*β* = 4.57, *p* = 0.04) as indicated by meta-regression (Supporting Information Table S4).

The number of longitudinal studies reporting detailed body composition in combination with metabolites and hormones was too low to perform meta-regressions for our secondary outcome the biochemical measures post-treatment and after weight recovery, indicating that the published literature is insufficient to draw conclusions at the current stage.

**Methodological moderators**

Furthermore, femoral neck bone mineral density (*β_Outpatient_* = -0.12, *p* = 7.65 x 10^-4^), leptin (*β_Outpatient_* = 4.65, *p* = 0.04), fasting glucose (*β_Outpatient_* = -10.52, *p* = 0.01), estradiol (*β_Outpatient_* = -29.23, *p* = 1.01 x 10^-11^), and IGF-1 (*β_Outpatient_* = -40.22, *p* = 0.004) significantly differed between inpatients and outpatients. Ghrelin concentrations (*β_Serum_* = -173.11, *p* = 6.41 x 10^-7^) were the only blood parameter associated with blood sample type, indicating that concentrations of all other blood parameters were comparable between serum and plasma.

**3) Meta-analyses of study sample characteristics comparing anorexia nervosa patients with healthy controls**

**
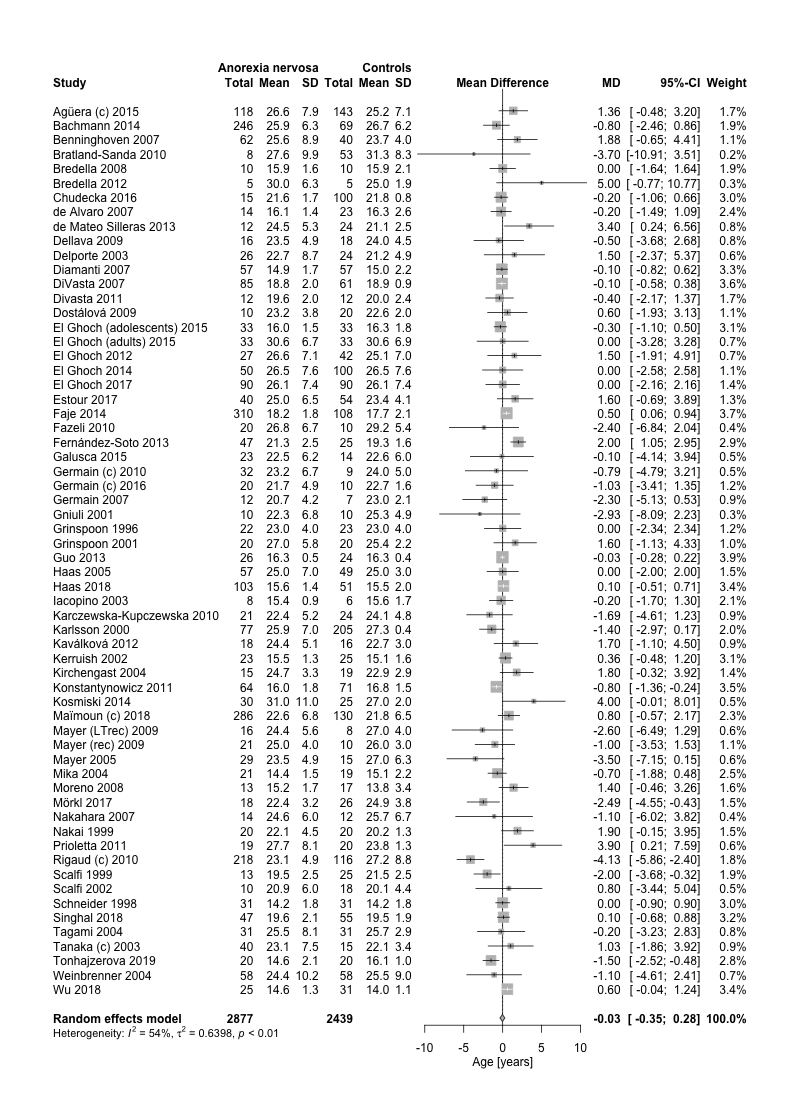
**

**Figure S1.** Cross-sectional meta-analysis of studies reporting mean age in anorexia nervosa patients compared with healthy controls. Sixty-two samples had the appropriate data for the meta-analysis with 2,877 AN cases and 2439 controls. A random-effects meta-analysis revealed a pooled estimate of the mean difference (MD: -0.03 years; 95% CI: -0.35, 0.28; *P* = 0.83). C, subtype-combined sample; LTrec, long term weight-recovered; rec, recovered.

**
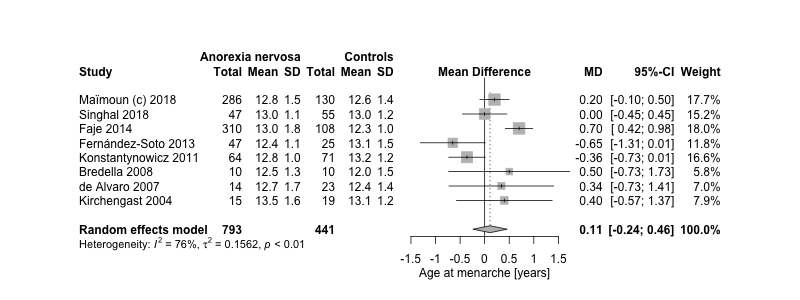
**

**Figure S2.** Cross-sectional meta-analysis of studies reporting age at menarche in female anorexia nervosa patients compared with healthy controls. Eight samples had the appropriate data for the meta-analysis with 793 AN cases and 441 controls. A random-effects meta-analysis revealed a pooled estimate of the mean difference (MD: 0.11 years; 95% CI: -0.24, -0.46; *P* = 0.54) with the mean differences ranging from -0.65 years to 0.70 years. Heterogeneity between studies was statistically significant (*τ^2^* = 0.16; *P* < 0.01; *I^2^* = 76%). C, subtype-combined sample.

**4) Q value plot of meta-analyses uncapped**


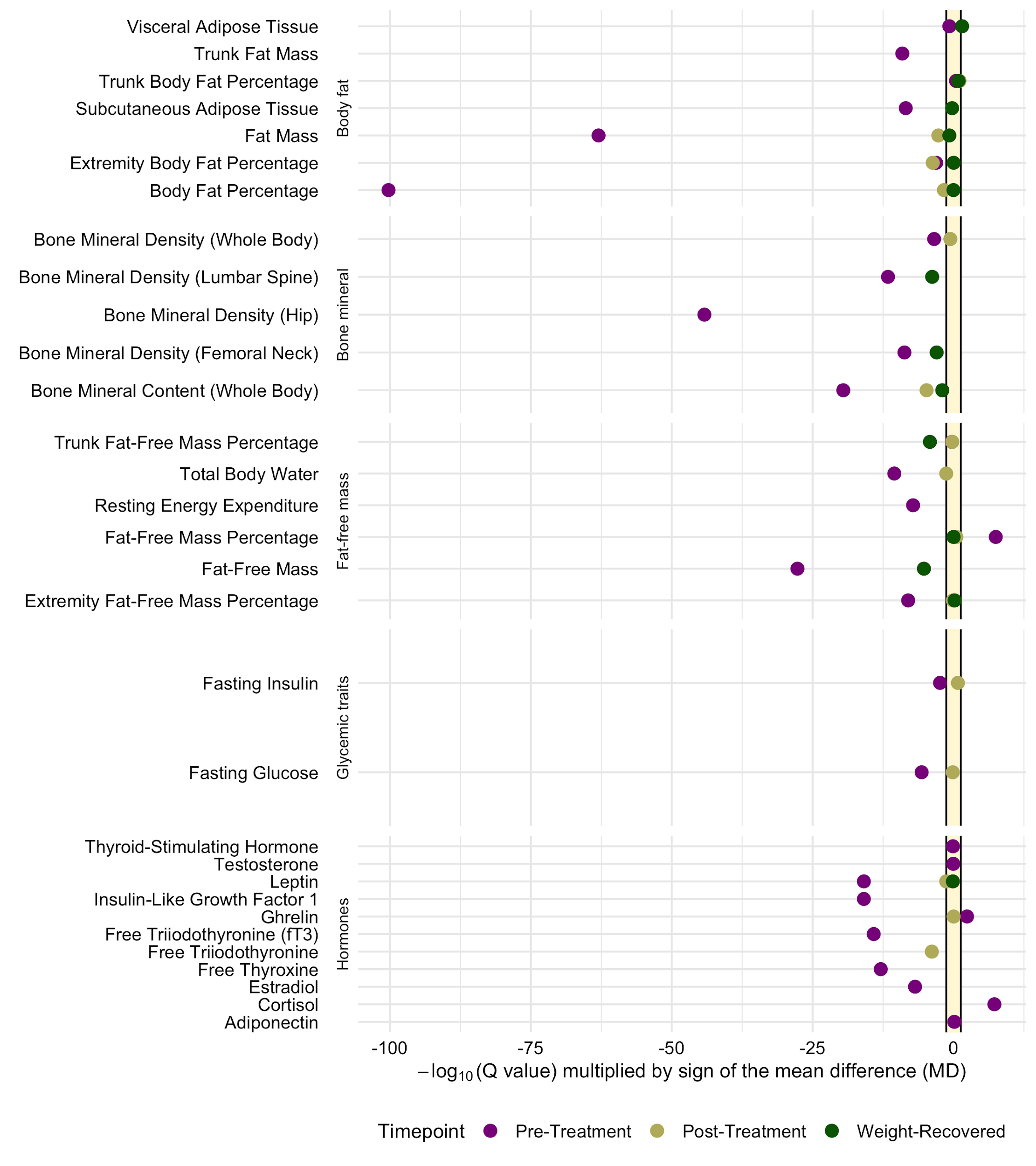


**Figure S3** Plot summarising the *Q values* of the meta-analyses comparing anorexia nervosa cases pre-treatment, post-treatment, and after weight recovery with healthy controls. The further the point on the right the larger the mean difference between cases and controls. *Q values* are transformed on the -log_10_ scale. The horizontal line represents the significance threshold of *Q* = 0.05.

**5) Cross-sectional meta-analyses of studies comparing acutely-ill/pre-treatment anorexia nervosa patients with healthy controls**


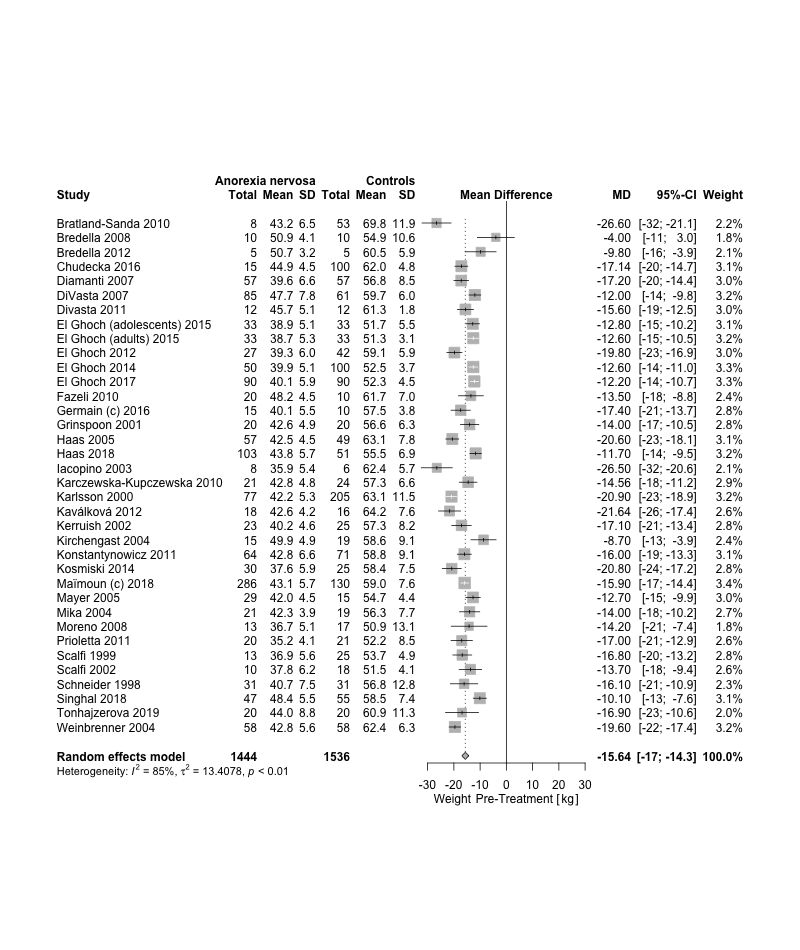


**Figure S4.** Cross-sectional meta-analysis of studies reporting weight in acutely-ill/pre-treatment female anorexia nervosa patients compared with healthy controls. Thirty-six samples had the appropriate data for the meta-analysis with 1444 AN cases and 1536 controls. A random-effects meta-analysis revealed a pooled estimate of the mean difference (MD: -15.64 kg; 95% CI: -16.98 , -14.30; *P* = 1.27 x 10^-115^) with the mean differences ranging from -26.60 kg to -4.00 kg. Heterogeneity between studies was statistically highly significant (*τ^2^* = 13.41; *P* = 4.12 x 10^-30^; *I^2^* = 84.6%). C, subtype-combined sample.


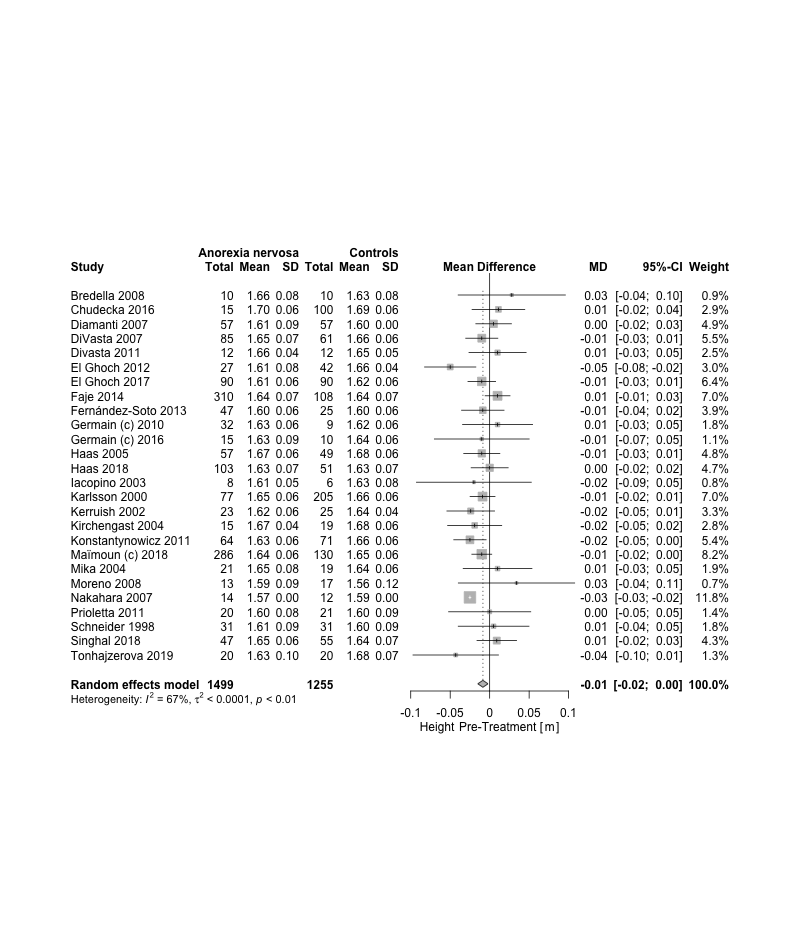


**Figure S5.** Cross-sectional meta-analysis of studies reporting height in acutely-ill/pre-treatment female anorexia nervosa patients compared with healthy controls. Twenty-six samples had the appropriate data for the meta-analysis with 1499 AN cases and 1255 controls. A random-effects meta-analysis revealed a pooled estimate of the mean difference (MD: -0.01 m; 95% CI: -0.02, 0.00; *P* = 1.01 x 10^-2^) with the mean differences ranging from -0.05 m to 0.03 m. Heterogeneity between studies was statistically highly significant (*τ^2^* = 0.0001; *P* = 6.86 x 10^-7^; *I^2^* = 66.7%). C, subtype-combined sample.


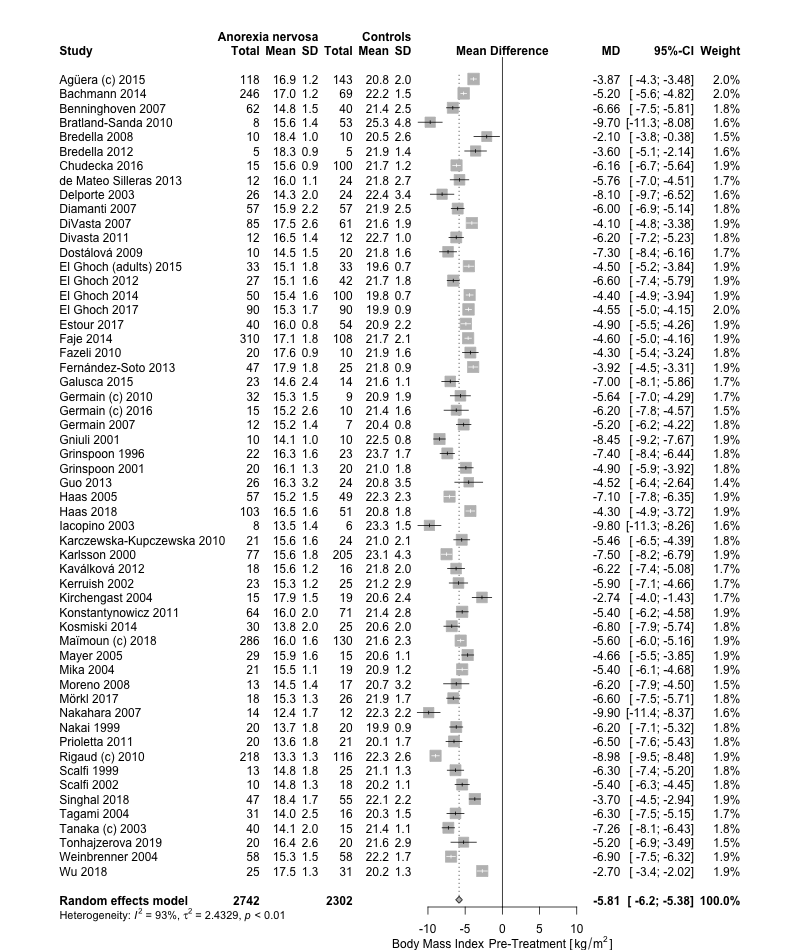


**Figure S6.** Cross-sectional meta-analysis of studies reporting body mass index in acutely-ill/pre-treatment female anorexia nervosa patients compared with healthy controls. Fifty-six samples had the appropriate data for the meta-analysis with 2,742 AN cases and 2,302 controls. A random-effects meta-analysis revealed a pooled estimate of the mean difference (MD: -5.81 kg/m^2^; 95% CI: -6.25, -5.38; *P* = 3.22 x 10^-154^) with the mean differences ranging from -9.90 kg/m^2^ to -2.10 kg/m^2^. Heterogeneity between studies was statistically significant (*τ^2^* = 2.43; *P* = 2.27 x 10^-140^; *I^2^* = 93.4%). C, subtype-combined sample.


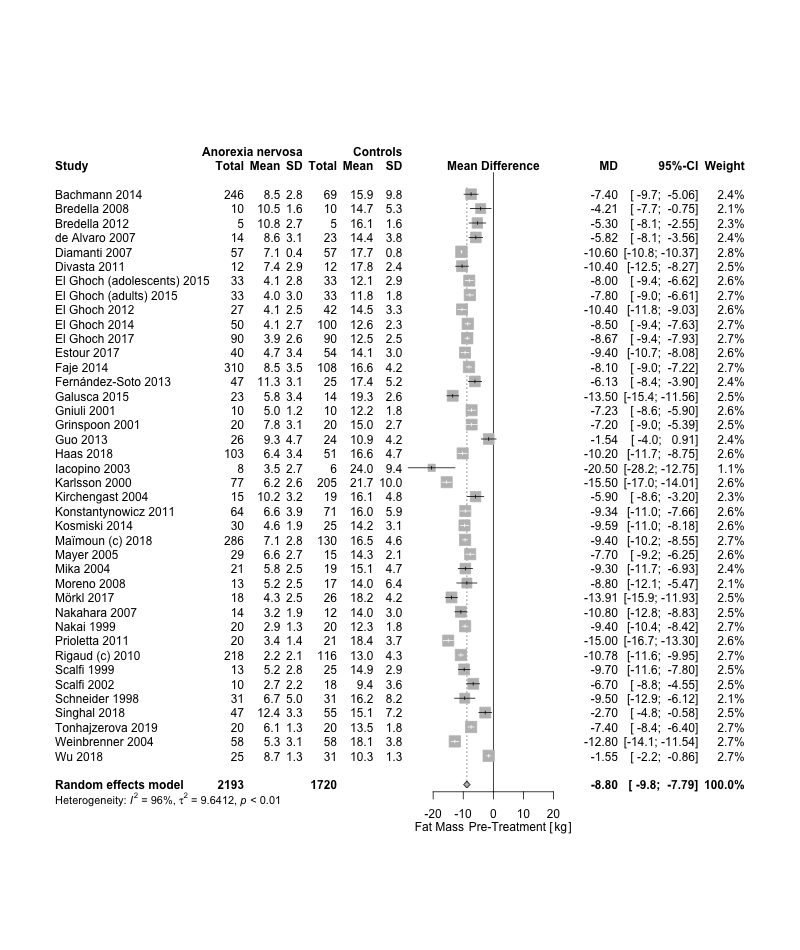


**Figure S7.** Cross-sectional meta-analysis of studies reporting fat mass in acutely-ill/pre-treatment female anorexia nervosa patients compared with healthy controls. Forty samples had the appropriate data for the meta-analysis with 2193 AN cases and 1720 controls. A random-effects meta-analysis revealed a pooled estimate of the mean difference (MD: -8.80 kg; 95% CI: -9.81, -7.79; *P* = 4.58 x 10^-65^) with the mean differences ranging from -20.50 kg to -1.54 kg. Heterogeneity between studies was statistically significant (*τ^2^* = 9.64; *P* = 2.97 x 10^-181^; *I^2^* = 96.0%). C, subtype-combined sample.


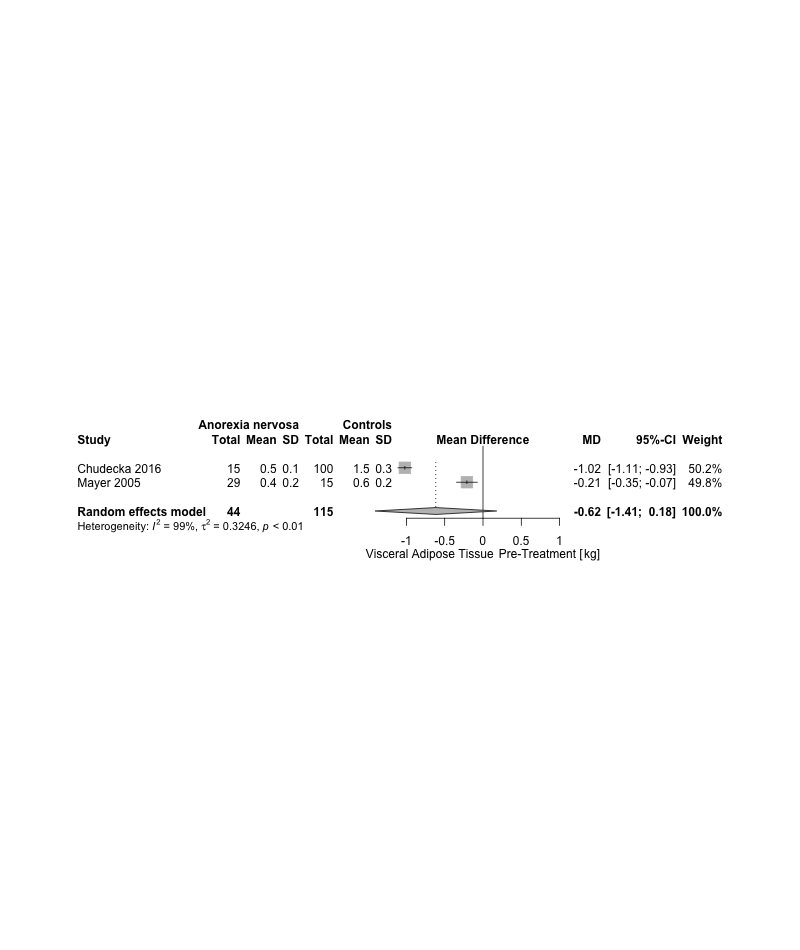


**Figure S8.** Cross-sectional meta-analysis of studies reporting visceral adipose tissue in acutely-ill/pre-treatment female anorexia nervosa patients compared with healthy controls. Two samples had the appropriate data for the meta-analysis with 44 AN cases and 115 controls. A random-effects meta-analysis revealed a pooled estimate of the mean difference (MD: -0.62 kg; 95% CI: -1.41, 0.18; *P* = 0.13) with the mean differences ranging from -1.02 kg to -0.21 kg. Heterogeneity between studies was statistically significant (*τ^2^* = 0.32; *P* = 1.67 x 10^-22^; *I^2^* = 99.0%).


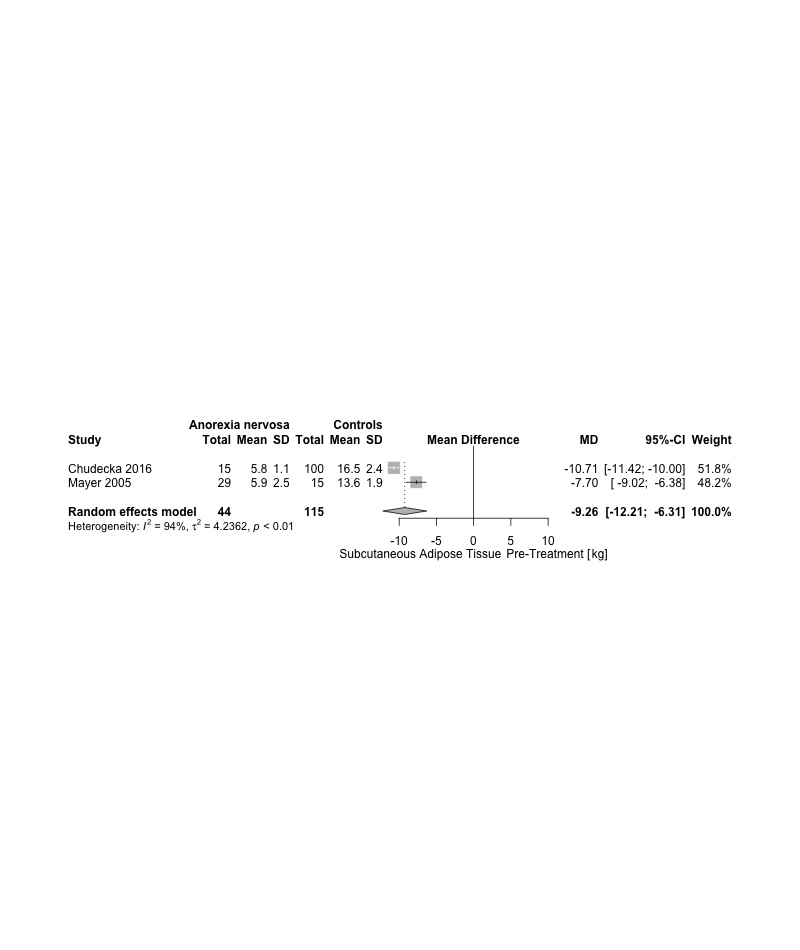


**Figure S9.** Cross-sectional meta-analysis of studies reporting subcutaneous adipose tissue in acutely-ill/pre-treatment female anorexia nervosa patients compared with healthy controls. Two samples had the appropriate data for the meta-analysis with 44 AN cases and 115 controls. A random-effects meta-analysis revealed a pooled estimate of the mean difference (MD: -9.26 kg; 95% CI: -12.21, -6.31; *P* = 7.46 x 10^-10^) with the mean differences ranging from -10.71 kg to -7.70 kg. Heterogeneity between studies was statistically significant (*τ^2^* = 4.24; *P* = 8.63 x 10^-5^; *I^2^* = 93.5%).


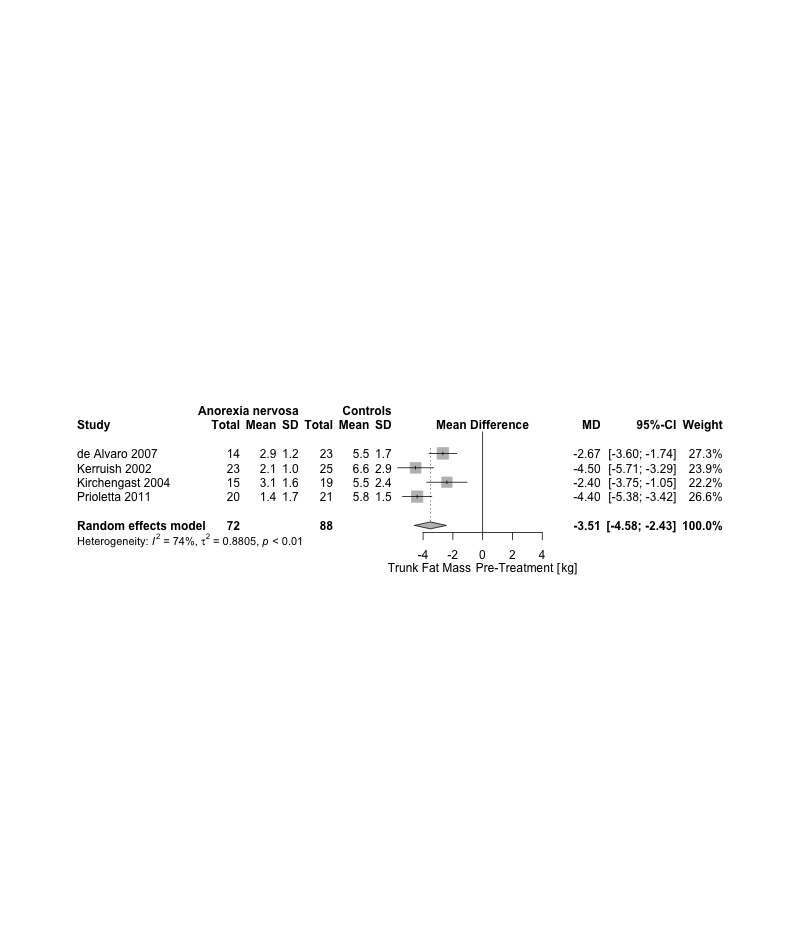


**Figure S10.** Cross-sectional meta-analysis of studies reporting trunk fat mass in acutely-ill/pre-treatment female anorexia nervosa patients compared with healthy controls. Four samples had the appropriate data for the meta-analysis with 72 AN cases and 88 controls. A random-effects meta-analysis revealed a pooled estimate of the mean difference (MD: -3.51 kg; 95% CI: -4.58, -2.43; *P* = 1.65 x 10^-10^) with the mean differences ranging from -4.50 kg to -2.40 kg. Heterogeneity between studies was statistically significant (*τ^2^* = 0.88; *P* = 9.46 x 10^-3^; *I^2^* = 73.8%).


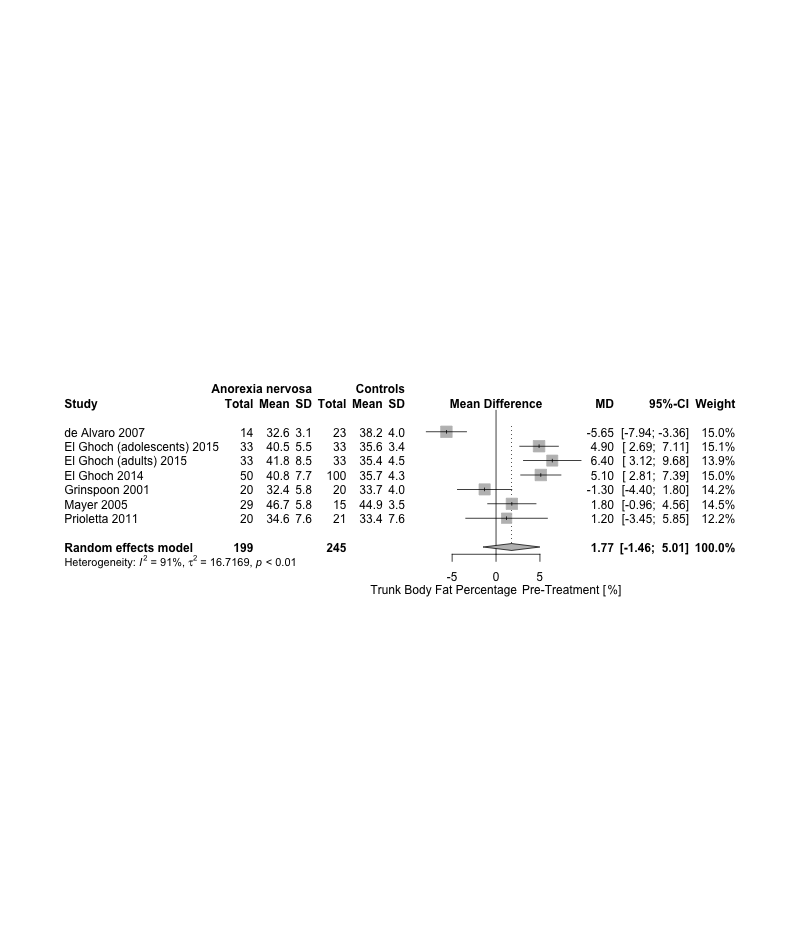


**Figure S11.** Cross-sectional meta-analysis of studies reporting trunk body fat percentage in acutely-ill/pre-treatment female anorexia nervosa patients compared with healthy controls. Seven samples had the appropriate data for the meta-analysis with 199 AN cases and 245 controls. A random-effects meta-analysis revealed a pooled estimate of the mean difference (MD: 1.7%; 95% CI: -1.4, 5.0; *P* = 0.28) with the mean differences ranging from -5.6% to 6.4%. Heterogeneity between studies was statistically significant (*τ^2^* = 16.72; *P* = 1.36 x 10^-12^; *I^2^* = 91.1%).


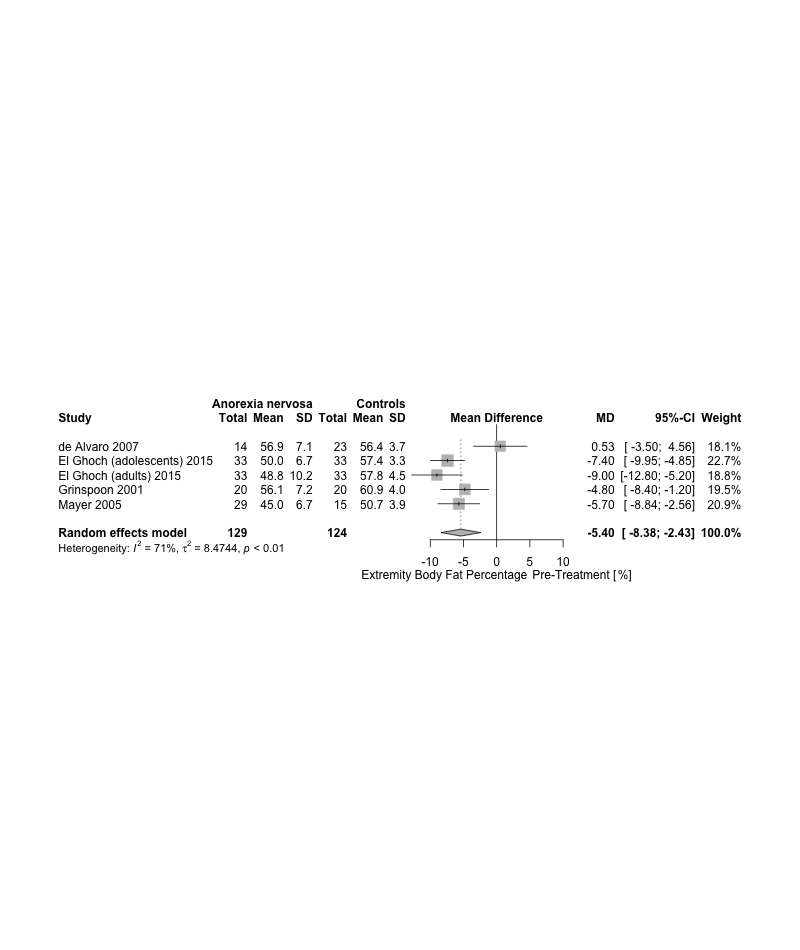


**Figure S12.** Cross-sectional meta-analysis of studies reporting extremity body fat percentage in acutely-ill/pre-treatment female anorexia nervosa patients compared with healthy controls. Five samples had the appropriate data for the meta-analysis with 129 AN cases and 124 controls. A random-effects meta-analysis revealed a pooled estimate of the mean difference (MD: -5.4%; 95% CI: -8.3, -2.4; *P* = 3.74 x 10^-4^) with the mean differences ranging from -9.0% to 0.5%. Heterogeneity between studies was statistically significant (*τ^2^* = 8.47; *P* = 0.01; *I^2^* = 71.5%).


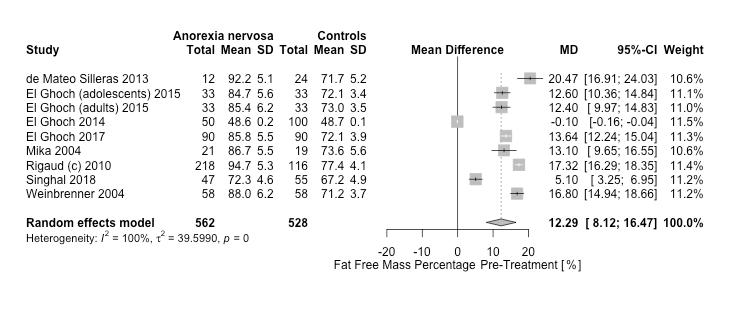


**Figure S13.** Cross-sectional meta-analysis of studies reporting fat free mass percentage in acutely-ill/pre-treatment female anorexia nervosa patients compared with healthy controls. Nine samples had the appropriate data for the meta-analysis with 562 AN cases and 528 controls. A random-effects meta-analysis revealed a pooled estimate of the mean difference (MD: 12.3%; 95% CI: 8.1, 16.5; *P* = 8.03 x 10^-9^) with the mean differences ranging from -0.1% to 20.5%. Heterogeneity between studies was statistically significant (*τ^2^* = 39.60; *P* = 0.00; *I^2^* = 99.6%). C, subtype-combined sample.


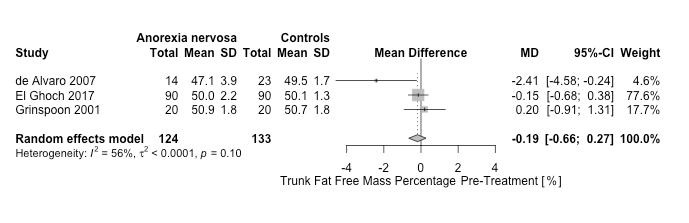


**Figure S14.** Cross-sectional meta-analysis of studies reporting trunk fat free mass percentage in acutely-ill/pre-treatment female anorexia nervosa patients compared with healthy controls. Three samples had the appropriate data for the meta-analysis with 124 AN cases and 133 controls. A random-effects meta-analysis revealed a pooled estimate of the mean difference (MD: -0.2%; 95% CI: -0.6, 0.3; *P* = 0.42) with the mean differences ranging from -2.4% to 0.2%. Heterogeneity between studies was not statistically significant (*τ^2^* =0.00; *P* = 0.10).


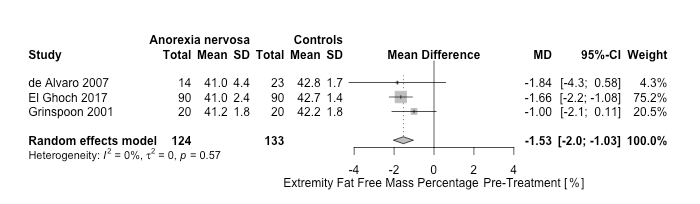
**Figure S15.** Cross-sectional meta-analysis of studies reporting extremity fat free mass percentage in acutely-ill/pre-treatment female anorexia nervosa patients compared with healthy controls. Three samples had the appropriate data for the meta-analysis with 124 AN cases and 133 controls. A random-effects meta-analysis revealed a pooled estimate of the mean difference (MD: -1.5%; 95% CI: -2.0, -1.0; *P* = 8.07 x 10^-9^) with the mean differences ranging from -1.8% to 1.0%. There was no Heterogeneity between studies (*τ^2^* =0.00; *P* = 0.50; *I^2^* = 0.0%).


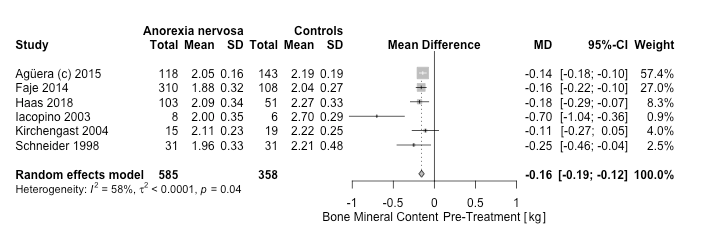


**Figure S16.** Cross-sectional meta-analysis of studies reporting bone mineral content in acutely-ill/pre-treatment female anorexia nervosa patients compared with healthy controls. Six samples had the appropriate data for the meta-analysis with 585 AN cases and 358 controls. A random-effects meta-analysis revealed a pooled estimate of the mean difference (MD: -0.16 kg; 95% CI: -0.19, -0.12; *P* = 3.10 x 10^-21^ ) with the mean differences ranging from -0.70 kg to -0.11 kg. Heterogeneity between studies was statistically significant (*τ^2^* = 0.00; *P* = 0.04; *I^2^* = 58.2%). C, subtype-combined sample.


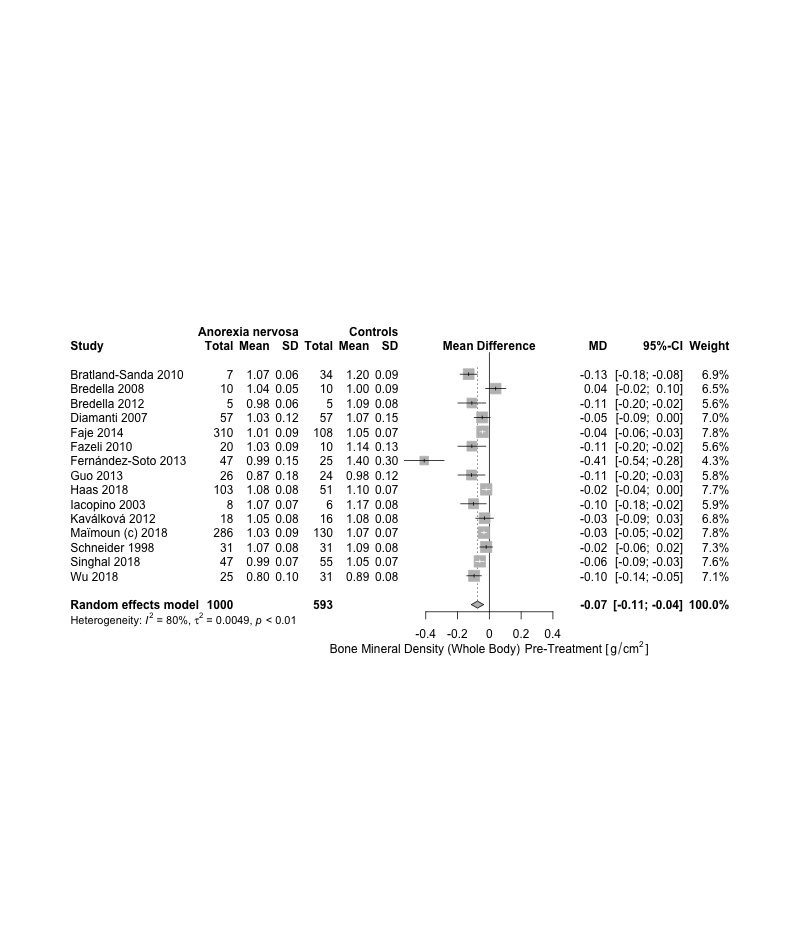


**Figure S17.** Cross-sectional meta-analysis of studies reporting bone mineral density (whole body) in acutely-ill/pre-treatment female anorexia nervosa patients compared with healthy controls. Fifteen samples had the appropriate data for the meta-analysis with 1000 AN cases and 593 controls. A random-effects meta-analysis revealed a pooled estimate of the mean difference (MD: -0.07 g/cm^2^; 95% CI: -0,11, -0.04; *P* = 1.64 x 10^-4^) with the mean differences ranging from -0.41 g/cm^2^ to 0.04 g/cm^2^. Heterogeneity between studies was statistically significant (*τ^2^* = 0.005; *P* = 1.30 x 10^-9^; *I^2^* = 80.3%). C, subtype-combined sample.


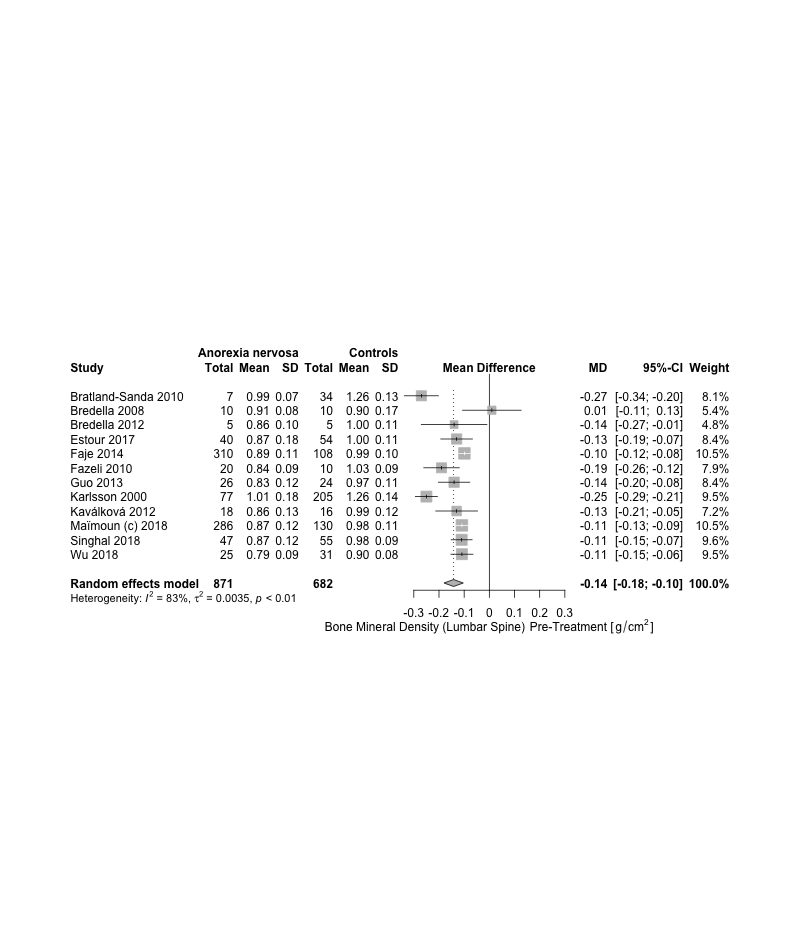


**Figure S18.** Cross-sectional meta-analysis of studies reporting bone mineral density (lumbar spine) in acutely-ill/pre-treatment female anorexia nervosa patients compared with healthy controls. Twelve samples had the appropriate data for the meta-analysis with 871 AN cases and 682 controls. A random-effects meta-analysis revealed a pooled estimate of the mean difference (MD: -0.14 g/cm^2^; 95% CI: -0.18, -0.10; *P* = 4.22 x 10^-13^) with the mean differences ranging from -0.27 g/cm^2^ to 0.01 g/cm^2^. Heterogeneity between studies was statistically significant (*τ^2^* = 0.004; *P* = 9.28 x 10^-10^; *I^2^* = 83.2%). C, subtype-combined sample.


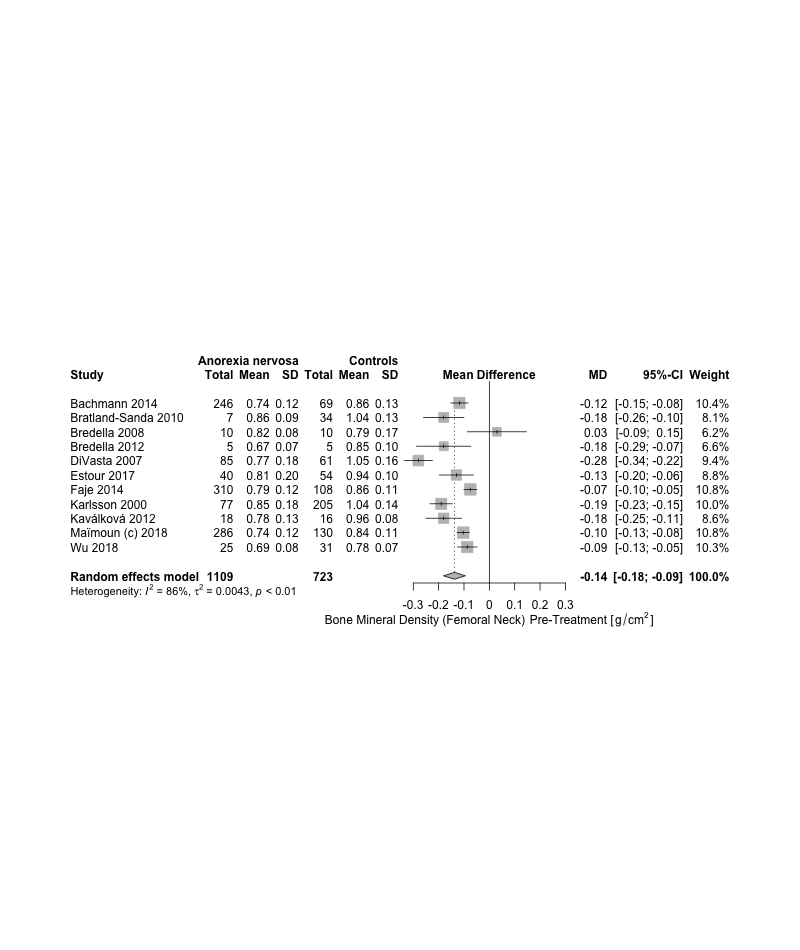


**Figure S19.** Cross-sectional meta-analysis of studies reporting bone mineral density (femoral neck) in acutely-ill/pre-treatment female anorexia nervosa patients compared with healthy controls. Eleven samples had the appropriate data for the meta-analysis with 1,109 AN cases and 723 controls. A random-effects meta-analysis revealed a pooled estimate of the mean difference (MD: -0.14 g/cm^2^; 95% CI: -0.18, -0.09; *P* = 4.16 x 10^-10^) with the mean differences ranging from -0.28 g/cm^2^ to 0.03 g/cm^2^. Heterogeneity between studies was statistically significant (*τ^2^* = 0.004; *P* = 2.98 x 10^-11^; *I^2^* = 85.9%). C, subtype-combined sample.


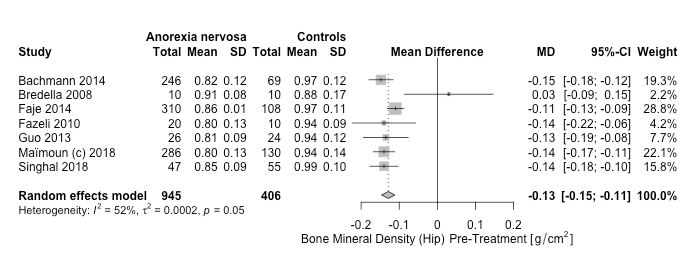


**Figure S20.** Cross-sectional meta-analysis of studies reporting bone mineral density (hip) in acutely-ill/pre-treatment female anorexia nervosa patients compared with healthy controls. Seven samples had the appropriate data for the meta-analysis with 945 AN cases and 406 controls. A random-effects meta-analysis revealed a pooled estimate of the mean difference (MD: -0.13 g/cm^2^; 95% CI: -0.15, -0.11; *P* = 3.52 x 10^-46^) with the mean differences ranging from -0.15 g/cm^2^ to 0.03 g/cm^2^. Heterogeneity between studies was statistically significant (*τ^2^* = 2.00 x 10^-4^; *P* = 0.05; *I^2^* = 52.0%). C, subtype-combined sample.


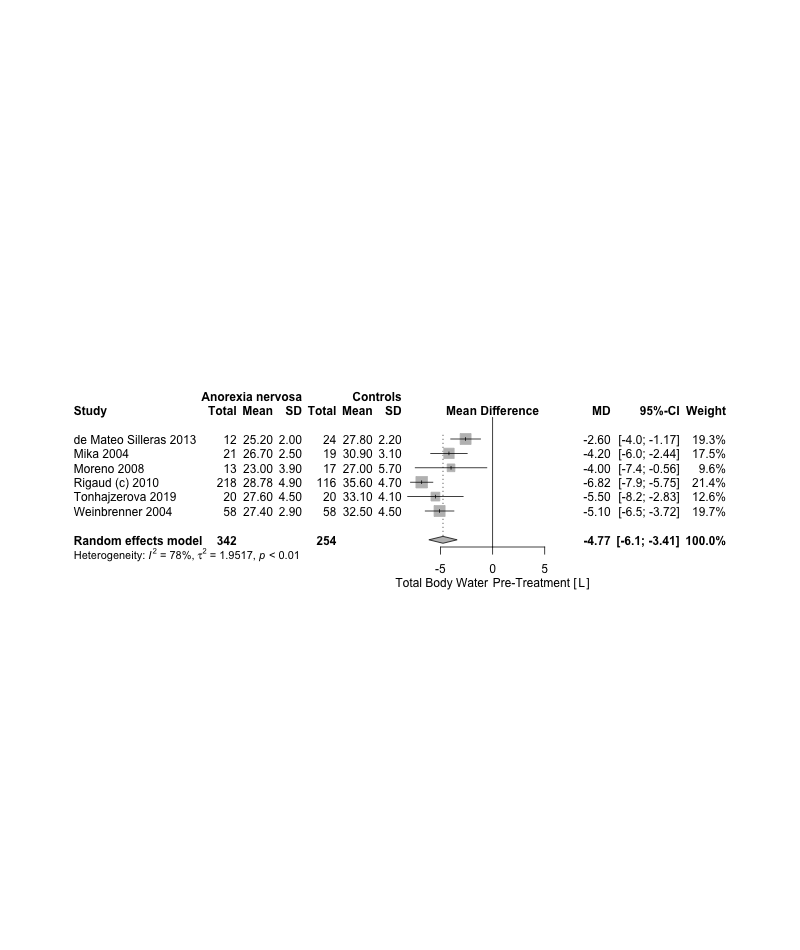


**Figure S21.** Cross-sectional meta-analysis of studies reporting total body water in acutely-ill/pre-treatment female anorexia nervosa patients compared with healthy controls. Six samples had the appropriate data for the meta-analysis with 342 AN cases and 254 controls. A random-effects meta-analysis revealed a pooled estimate of the mean difference (MD: -4.77 L; 95% CI: -6.13, -3.41; *P* = 5.92 x 10^-12^) with the mean differences ranging from -6.82 L to -2.60 L. Heterogeneity between studies was statistically significant (*τ^2^* = 1.95; *P* = 3.36 x 10^-4^; *I^2^* = 78.3%). C, subtype-combined sample.


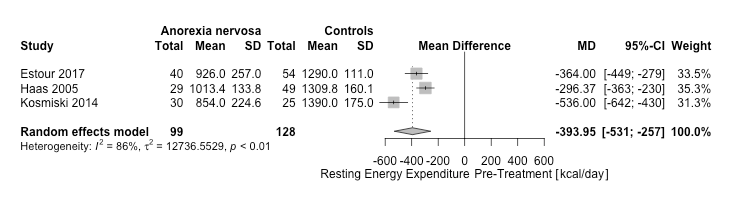


**Figure S22.** Cross-sectional meta-analysis of studies reporting resting energy expenditure in acutely-ill/pre-treatment female anorexia nervosa patients compared with healthy controls. Three samples had the appropriate data for the meta-analysis with 99 AN cases and 128 controls. A random-effects meta-analysis revealed a pooled estimate of the mean difference (MD: -393.95 kcal/day; 95% CI: -531.04, -256.86; *P* = 1.78 x 10^-8^) with the mean differences ranging from -536.00 kcal/day to -296.37 kcal/day. Heterogeneity between studies was statistically significant (*τ^2^* = 12736.55; *P* = 8.30 x 10^-4^; *I^2^* = 85.9%).


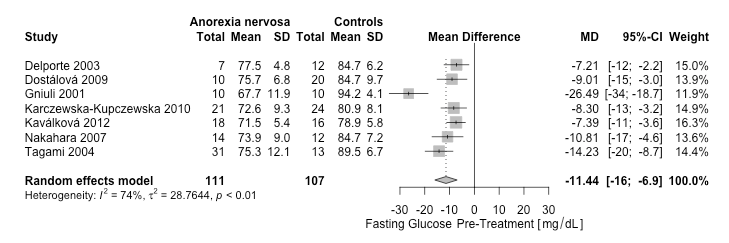


**Figure S23.** Cross-sectional meta-analysis of studies reporting fasting glucose in acutely-ill/pre-treatment female anorexia nervosa patients compared with healthy controls. Seven samples had the appropriate data for the meta-analysis with 111 AN cases and 107 controls. A random-effects meta-analysis revealed a pooled estimate of the mean difference (MD: -11.44 mg/dL; 95% CI: -15.95, -6.93; *P* = 6.71 x 10^-7^) with the mean differences ranging from -26.49 mg/dL to -7.21 mg/dL. Heterogeneity between studies was statistically highly significant (*τ^2^* = 28.76; *P* = 8.04 x 10^-4^; *I^2^* = 73.9%).


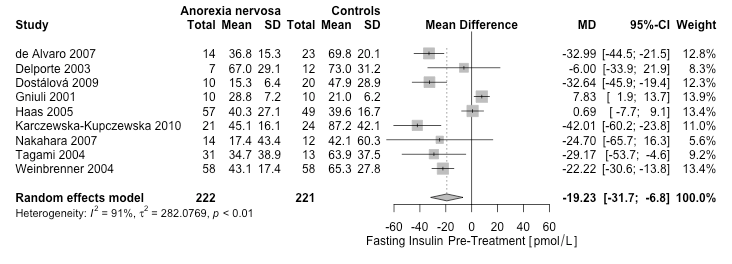


**Figure S24.** Cross-sectional meta-analysis of studies reporting fasting insulin in acutely-ill/pre-treatment female anorexia nervosa patients compared with healthy controls. Nine samples had the appropriate data for the meta-analysis with 222 AN cases and 221 controls. A random-effects meta-analysis revealed a pooled estimate of the mean difference (MD: -19.23 pmol/L; 95% CI: -31.68, -6.77; *P* = 2.49 x 10^-3^) with the mean differences ranging from -42.01 pmol/L to 7.83 pmol/L. Heterogeneity between studies was statistically highly significant (*τ^2^* = 282.08; *P* = 3.43 x 10^-16^; *I^2^* = 91.2%).


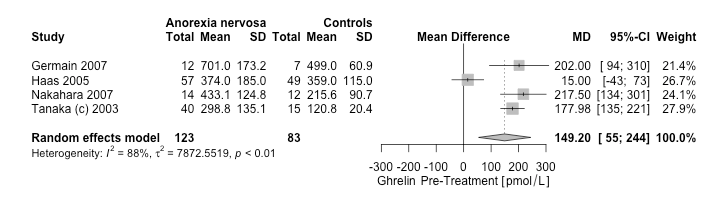


**Figure S25.** Cross-sectional meta-analysis of studies reporting ghrelin in acutely-ill/pre-treatment female anorexia nervosa patients compared with healthy controls. Four samples had the appropriate data for the meta-analysis with 123 AN cases and 83 controls. A random-effects meta-analysis revealed a pooled estimate of the mean difference (MD: 149.20 pmol/L; 95% CI: 54.59, 243.81; *P* = 2.00 x 10^-3^) with the mean differences ranging from 15.00 pmol/L to 217.50 pmol/L. Heterogeneity between studies was statistically highly significant (*τ^2^* = 7872.55; *P* = 1.19 x 10^-5^; *I^2^* = 88.3%). C, subtype-combined sample.


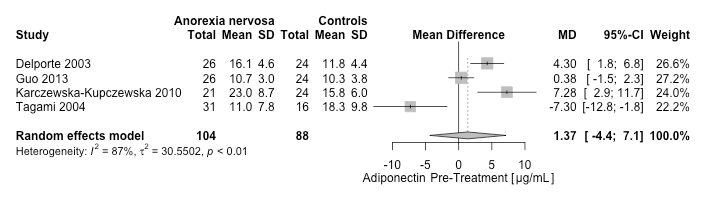


**Figure S26.** Cross-sectional meta-analysis of studies reporting adiponectin in acutely-ill/pre-treatment female anorexia nervosa patients compared with healthy controls. Four samples had the appropriate data for the meta-analysis with 104 AN cases and 88 controls. A random-effects meta-analysis revealed a pooled estimate of the mean difference (MD: 1.37 μg/mL; 95% CI: -4.36, 7.11; *P* = 0.64) with the mean differences ranging from -7.30 μg/mL to 7.28 μg/mL. Heterogeneity between studies was statistically significant (*τ^2^* = 30.55; *P* = 5.62 x 10^-5^; *I^2^* = 86.6%).


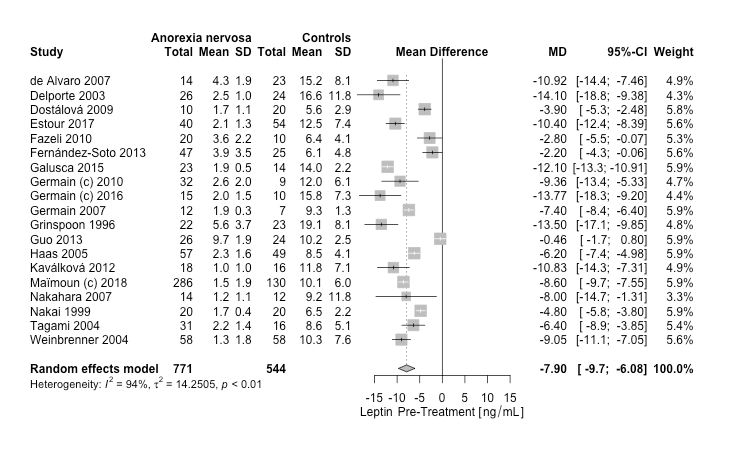


**Figure S27.** Cross-sectional meta-analysis of studies reporting leptin in acutely-ill/pre-treatment female anorexia nervosa patients compared with healthy controls. Nineteen samples had the appropriate data for the meta-analysis with 771 AN cases and 544 controls. A random-effects meta-analysis revealed a pooled estimate of the mean difference (MD: -7.90 ng/mL; 95% CI: -9.72, -6.08; *P* = 1.55 x 10^-17^) with the mean differences ranging from -14.10 ng/mL to -0.46 ng/mL. Heterogeneity between studies was statistically significant (*τ^2^* = 14.25; *P* = 6.47 x 10^-54^; *I^2^* = 94.1%). C, subtype-combined sample.


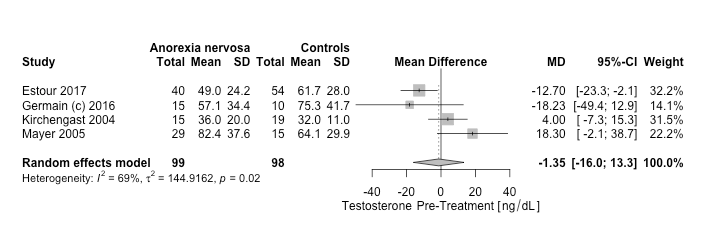


**Figure S28.** Cross-sectional meta-analysis of studies reporting testosterone in acutely-ill/pre-treatment female anorexia nervosa patients compared with healthy controls. Four samples had the appropriate data for the meta-analysis with 99 AN cases and 98 controls. A random-effects meta-analysis revealed a pooled estimate of the mean difference (MD: -1.35 ng/dL; 95% CI: -16.03, 13.33; *P* = 0.86) with the mean differences ranging from -18.23 ng/dL to 18.30 ng/dL. Heterogeneity between studies was statistically significant (*τ^2^* = 144.92; *P* = 0.02; *I^2^* = 69.4%). C, subtype-combined sample.


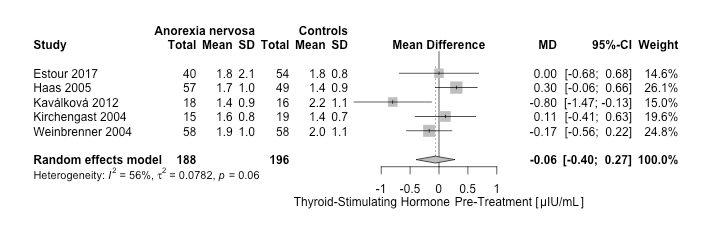


**Figure S29.** Cross-sectional meta-analysis of studies reporting thyroid-stimulating hormone in acutely-ill/pre-treatment female anorexia nervosa patients compared with healthy controls. Five samples had the appropriate data for the meta-analysis with 188 AN cases and 196 controls. A random-effects meta-analysis revealed a pooled estimate of the mean difference (MD: -0.06 μIU/mL; 95% CI: -0.40, 0.27; *P* = 0.72) with the mean differences ranging from -0.80 μIU/mL to 0.30 μIU/mL. Heterogeneity between studies was not statistically significant (*τ^2^* = 0.08; *P* = 0.06; *I^2^* = 55.5%).


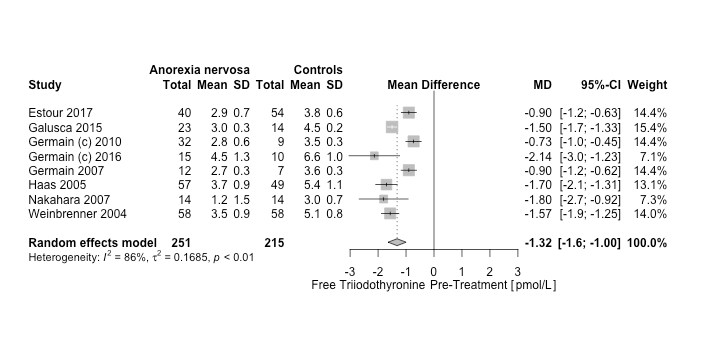


**Figure S30.** Cross-sectional meta-analysis of studies reporting free triiodothyronine in acutely-ill/pre-treatment female anorexia nervosa patients compared with healthy controls. Eight samples had the appropriate data for the meta-analysis with 251 AN cases and 215 controls. A random-effects meta-analysis revealed a pooled estimate of the mean difference (MD: -1.32 pmol/L; 95% CI: -1.64, -1.00; *P* = 1.09 x 10^-15^) with the mean differences ranging from -2.14 pmol/L to -0.73 pmol/L. Heterogeneity between studies was statistically significant (*τ^2^* = 0.17; *P* = 2.41 x 10^-8^; *I^2^* = 85.7%). C, subtype-combined sample.

**
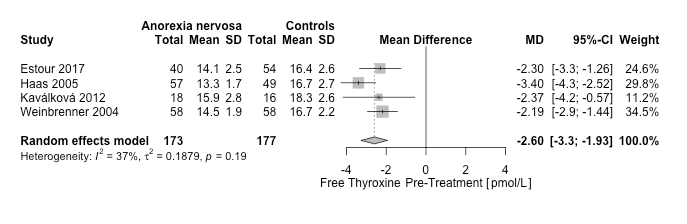
**

**Figure S31.** Cross-sectional meta-analysis of studies reporting free thyroxine in acutely-ill/pre-treatment female anorexia nervosa patients compared with healthy controls. Four samples had the appropriate data for the meta-analysis with 173 AN cases and 177 controls. A random-effects meta-analysis revealed a pooled estimate of the mean difference (MD: -2.60 pmol/L; 95% CI: -3.26, -1.93; *P* = 2.09 x 10^-14^) with the mean differences ranging from -3.40 pmol/L to -2.19 pmol/L. Heterogeneity between studies was not statistically significant (*τ^2^* = 0.19; *P* = 0.19; *I^2^* = 36.8%).


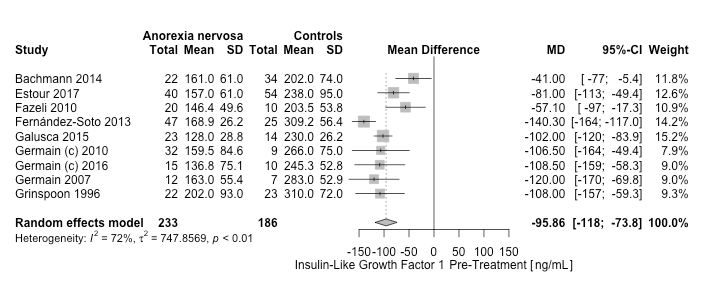


**Figure S32.** Cross-sectional meta-analysis of studies reporting insulin-like growth factor 1 in acutely-ill/pre-treatment female anorexia nervosa patients compared with healthy controls. Nine samples had the appropriate data for the meta-analysis with 233 AN cases and 186 controls. A random-effects meta-analysis revealed a pooled estimate of the mean difference (MD: -95.86 ng/mL; 95% CI: -117.93, -73.80; *P* = 1.67 x 10^-17^) with the mean differences ranging from -140.30 ng/mL to -41.00 ng/mL. Heterogeneity between studies was statistically significant (*τ^2^* = 747.86; *P* = 3.50 x 10^-4^; *I^2^* = 72.2%). C, subtype-combined sample.


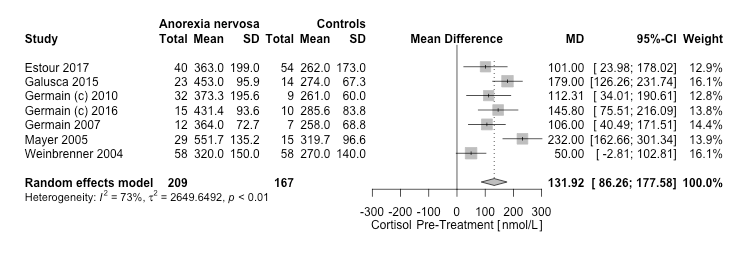


**Figure S33.** Cross-sectional meta-analysis of studies reporting cortisol in acutely-ill/pre-treatment female anorexia nervosa patients compared with healthy controls. Seven samples had the appropriate data for the meta-analysis with 209 AN cases and 167 controls. A random-effects meta-analysis revealed a pooled estimate of the mean difference (MD: 131.92 nmol/L; 95% CI: 86.26, 177.58; *P* = 1.49 x 10^-8^) with the mean differences ranging from 50.00 nmol/L to 232.00 nmol/L. Heterogeneity between studies was statistically significant (*τ^2^* = 2649.65; *P* = 1.17 x 10^-44^; *I^2^* = 72.6%). C, subtype-combined sample.


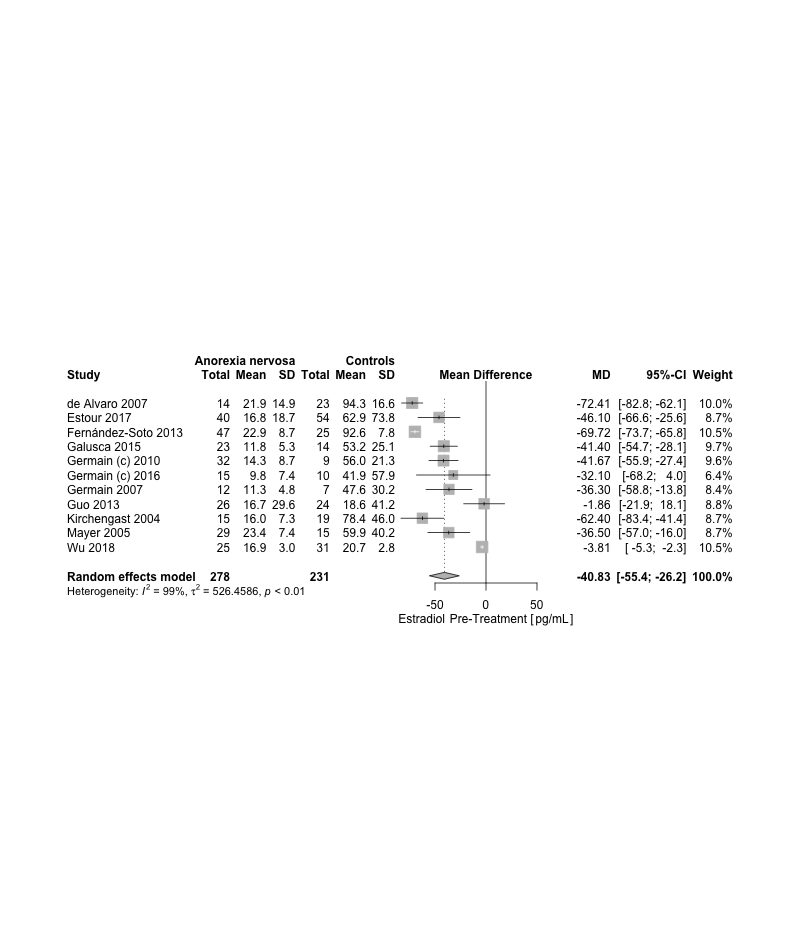


**Figure S34.** Cross-sectional meta-analysis of studies reporting estradiol in acutely-ill/pre-treatment female anorexia nervosa patients compared with healthy controls. Eleven samples had the appropriate data for the meta-analysis with 278 AN cases and 231 controls. A random-effects meta-analysis revealed a pooled estimate of the mean difference (MD: -40.83 pg/mL; 95% CI: -55.43, -26.23; *P* = 4.22 x 10^-8^) with the mean differences ranging from -72.41 pg/mL to -1.86 pg/mL. Heterogeneity between studies was statistically significant (*τ^2^* = 526.46; *P* = 9.60 x 10^-237^; *I^2^* = 99.1%). C, subtype-combined sample.

**6) Cross-sectional meta-analyses of studies comparing post-treatment anorexia nervosa patients with healthy controls**

**
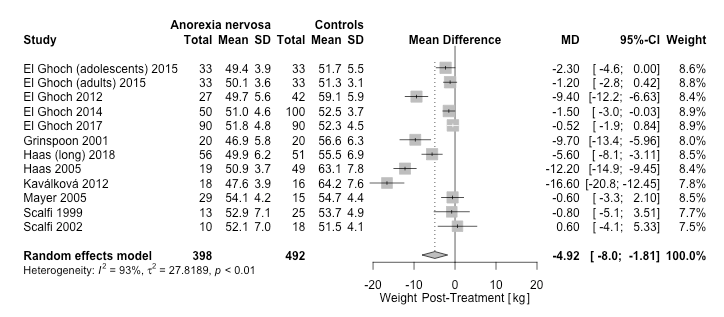
**

**Figure S35.** Cross-sectional meta-analysis of studies reporting weight in post-treatment female anorexia nervosa patients compared with healthy controls. Twelve samples had the appropriate data for the meta-analysis with 398 AN cases and 492 controls. A random-effects meta-analysis revealed a pooled estimate of the mean difference (MD: -4.92 kg; 95% CI: -8.03, -1.81; *P* = 1.92 x 10^-3^) with the mean differences ranging from -16.60 kg to 0.60 kg. Heterogeneity between studies was statistically highly significant (*τ^2^* = 27.82; *P* = 1.82 x 10^-26^; *I^2^* = 92.6%). C, subtype-combined sample; long, longitudinal.

**
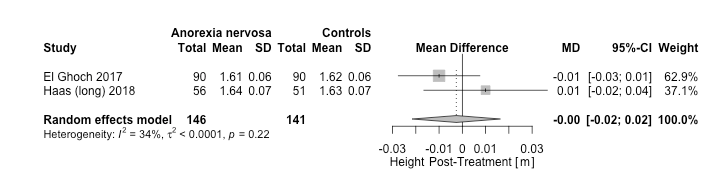
**

**Figure S36.** Cross-sectional meta-analysis of studies reporting height in post-treatment female anorexia nervosa patients compared with healthy controls. Two samples had the appropriate data for the meta-analysis with 146 AN cases and 141 controls. A random-effects meta-analysis revealed a pooled estimate of the mean difference (MD: -0.00 m; 95% CI: -0.02, 0.02; *P* = 0.79) with the mean differences ranging from -0.01 m to 0.01 m. Heterogeneity between studies was not statistically significant (*τ^2^* =0.00; *P* = 0.22; *I^2^* = 34.1%). Long, longitudinal.

**
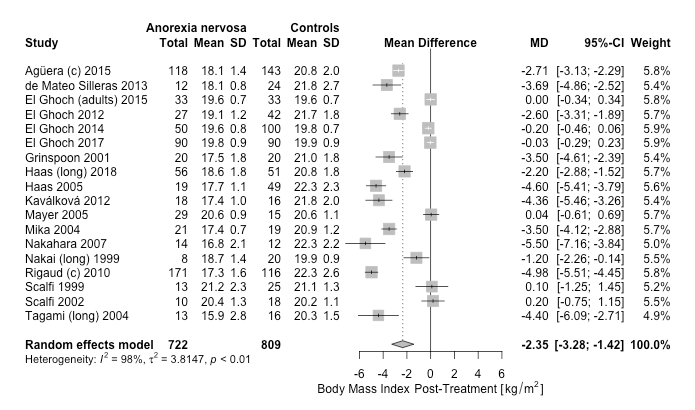
**

**Figure S37.** Cross-sectional meta-analysis of studies reporting body mass index in post-treatment female anorexia nervosa patients compared with healthy controls. Eighteen samples had the appropriate data for the meta-analysis with 722 AN cases and 809 controls. A random-effects meta-analysis revealed a pooled estimate of the mean difference (MD: -2.35 kg/m^2^; 95% CI: -3.28, -1.42; *P* = 6.79 x 10^-7^) with the mean differences ranging from -5.50 kg/m^2^ to 0.20 kg/m^2^. Heterogeneity between studies was statistically significant (*τ^2^* = 3.81; *P* = 3.96 x 10^-138^; *I^2^* = 97.6%). C, subtype-combined sample; long, longitudinal.


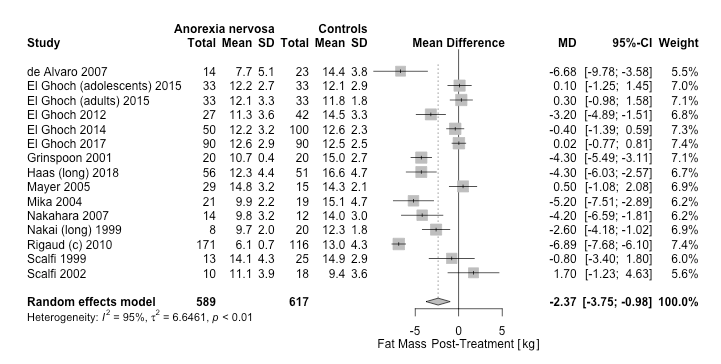


**Figure S38.** Cross-sectional meta-analysis of studies reporting fat mass in post-treatment female anorexia nervosa patients compared with healthy controls. Fifteen samples had the appropriate data for the meta-analysis with 589 AN cases and 617 controls. A random-effects meta-analysis revealed a pooled estimate of the mean difference (MD: -2.37 kg; 95% CI: -3.75, -0.98; *P* = 8.29 x 10^-4^) with the mean differences ranging from -6.89 kg to 1.70 kg. Heterogeneity between studies was statistically highly significant (*τ^2^* = 6.65; *P* = 5.45 x 10^-47^; *I^2^* = 94.6%). C, subtype-combined sample; long, longitudinal.


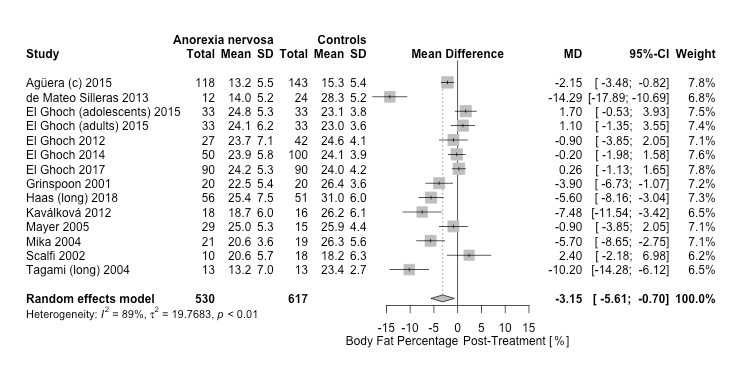


**Figure S39.** Cross-sectional meta-analysis of studies reporting body fat percentage in post-treatment female anorexia nervosa patients compared with healthy controls. Fourteen samples had the appropriate data for the meta-analysis with 530 AN cases and 617 controls. A random-effects meta-analysis revealed a pooled estimate of the mean difference (MD: -3.15 kg; 95% CI: -5.61, -0.70; *P* = 0.01) with the mean differences ranging from -14.3% to 2.4%. Heterogeneity between studies was statistically highly significant (*τ^2^* = 19.77; *P* = 5.37 x 10^-19^; *I^2^* = 89.0%). C, subtype-combined sample; long, longitudinal.


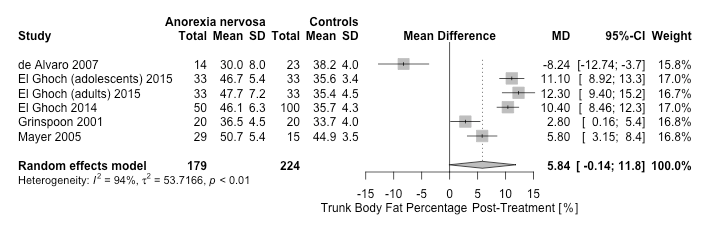


**Figure S40.** Cross-sectional meta-analysis of studies reporting trunk body fat percentage in post-treatment female anorexia nervosa patients compared with healthy controls. Six samples had the appropriate data for the meta-analysis with 179 AN cases and 224 controls. A random-effects meta-analysis revealed a pooled estimate of the mean difference (MD: 5.8%; 95% CI: -0.1, 11.8; *P* = 0.06) with the mean differences ranging from -8.2% to 12.3%. Heterogeneity between studies was statistically highly significant (*τ^2^* = 53.72; *P* = 7.59 x 10^-18^; *I^2^* = 94.4%).


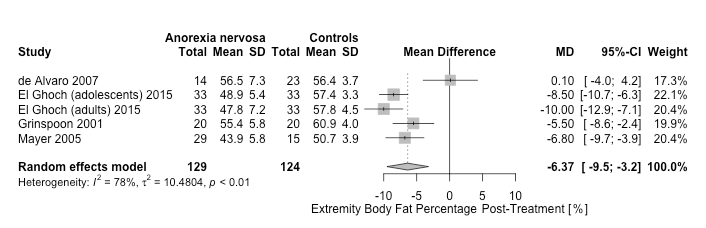


**Figure S41.** Cross-sectional meta-analysis of studies reporting extremity body fat percentage in post-treatment female anorexia nervosa patients compared with healthy controls. Five samples had the appropriate data for the meta-analysis with 129 AN cases and 124 controls. A random-effects meta-analysis revealed a pooled estimate of the mean difference (MD: -6.4%; 95% CI: -9.52, -3.23; *P* = 7.24 x 10^-5^) with the mean differences ranging from -10.0% to 0.0%. Heterogeneity between studies was statistically highly significant (*τ^2^* = 10.48; *P* = 1.09 x 10^-3^; *I^2^* = 78.1%).


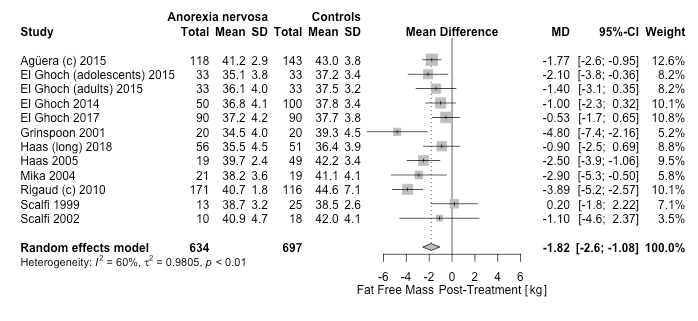


**Figure S42.** Cross-sectional meta-analysis of studies reporting fat free mass in post-treatment female anorexia nervosa patients compared with healthy controls. Twelve samples had the appropriate data for the meta-analysis with 634 AN cases and 697 controls. A random-effects meta-analysis revealed a pooled estimate of the mean difference (MD: -1.82 kg; 95% CI: -2.57, -1.08; *P* = 1.72 x 10^-6^) with the mean differences ranging from -4.80 kg to 0.20 kg. Heterogeneity between studies was statistically highly significant (*τ^2^* = 0.98; *P* = 3.70 x 10^-3^; *I^2^* = 60.2%). C, subtype-combined sample; long, longitudinal.

**
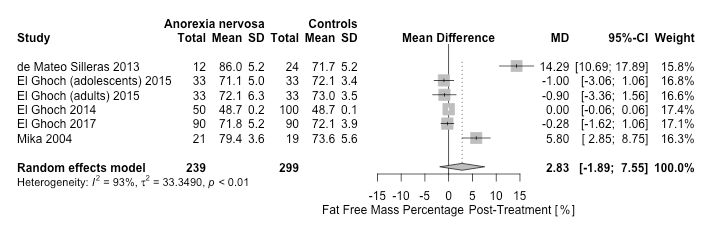
Figure S43.** Cross-sectional meta-analysis of studies reporting fat free mass percentage in post-treatment female anorexia nervosa patients compared with healthy controls. Six samples had the appropriate data for the meta-analysis with 239 AN cases and 299 controls. A random-effects meta-analysis revealed a pooled estimate of the mean difference (MD: 2.8%; 95% CI: -1.9, 7.6; *P* = 0.24) with the mean differences ranging from -1.0% to 14.3%. Heterogeneity between studies was statistically highly significant (*τ^2^* = 33.35; *P* = 3.89 x 10^-15^; *I^2^* = 93.5%).


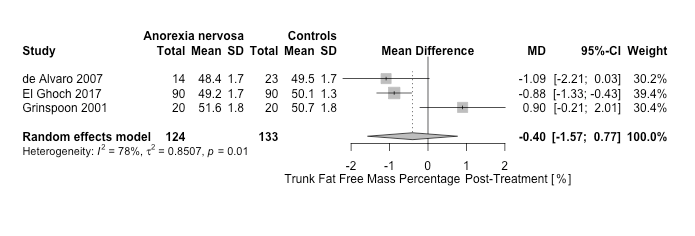
**Figure S44.** Cross-sectional meta-analysis of studies reporting trunk fat free mass percentage in post-treatment female anorexia nervosa patients compared with healthy controls. Three samples had the appropriate data for the meta-analysis with 124 AN cases and 133 controls. A random-effects meta-analysis revealed a pooled estimate of the mean difference (MD: -0.4%; 95% CI: -1.6, 0.8; *P* = 0.50) with the mean differences ranging from -1.1% to 0.9%. Heterogeneity between studies was statistically significant (*τ^2^* = 0.85; *P* = 0.01; *I^2^* = 77.9%).


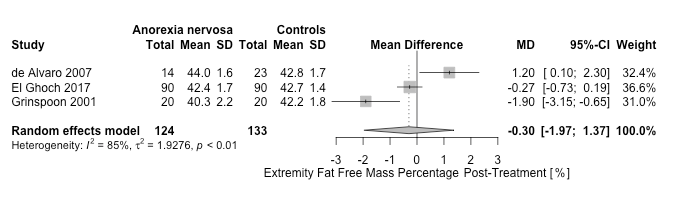


**Figure S45.** Cross-sectional meta-analysis of studies reporting extremity fat free mass percentage in post-treatment female anorexia nervosa patients compared with healthy controls. Three samples had the appropriate data for the meta-analysis with 124 AN cases and 133 controls. A random-effects meta-analysis revealed a pooled estimate of the mean difference (MD: -0.3%; 95% CI: -1.97, 1.37; *P* = 0.73) with the mean differences ranging from -1.9% to 1.2%. Heterogeneity between studies was statistically significant (*τ^2^* = 1.93; *P* = 1.26 x 10^-3^; *I^2^* = 85.0%).


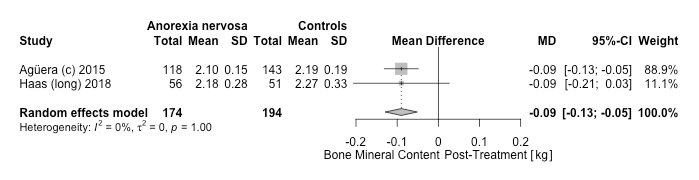


**Figure S46.** Cross-sectional meta-analysis of studies reporting bone mineral content in post-treatment female anorexia nervosa patients compared with healthy controls. Two samples had the appropriate data for the meta-analysis with 174 AN cases and 194 controls. A random-effects meta-analysis revealed a pooled estimate of the mean difference (MD: -0.09 kg; 95% CI: -0.13, -0.05; *P* = 5.75 x 10^-6^) with the mean differences ranging from -0.09 kg to -0.09 kg. There was no Heterogeneity between studies (*τ^2^* = 0.00; *P* = 1.00; *I^2^* = 0.0%). C, subtype-combined sample; long, longitudinal.


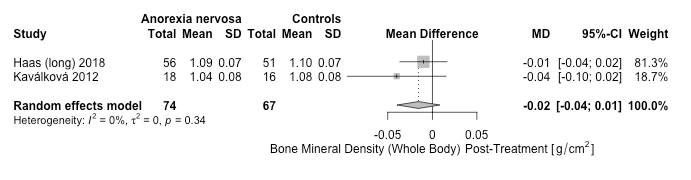


**Figure S47.** Cross-sectional meta-analysis of studies reporting bone mineral density (whole body) content in post-treatment female anorexia nervosa patients compared with healthy controls. Two samples had the appropriate data for the meta-analysis with 74 AN cases and 67 controls. A random-effects meta-analysis revealed a pooled estimate of the mean difference (MD: -0.02 g/cm^2^; 95% CI: -0.04, 0.01; *P* = 0.20) with the mean differences ranging from -0.04 g/cm^2^ to -0.01 g/cm^2^. There was no Heterogeneity between studies (*τ^2^* = 0.00; *P* = 0.34; *I^2^* = 0.0%). long, longitudinal.


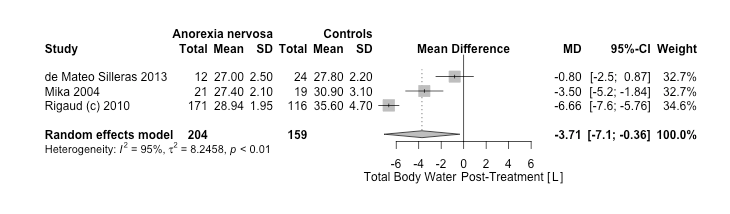


**Figure S48.** Cross-sectional meta-analysis of studies reporting total body water in post-treatment female anorexia nervosa patients compared with healthy controls. Three samples had the appropriate data for the meta-analysis with 204 AN cases and 159 controls. A random-effects meta-analysis revealed a pooled estimate of the mean difference (MD: -3.71 L; 95% CI: -7.07, -0.36; *P* = 0.03) with the mean differences ranging from -6.66 L to -0.80 L. Heterogeneity between studies was statistically highly significant (*τ^2^* = 8.25; *P* = 1.59 x 10^-9^; *I^2^* = 95.1%). C, subtype-combined sample.


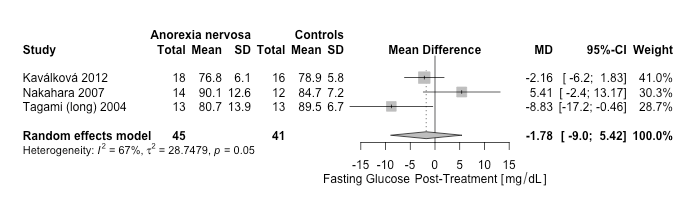


**Figure S49.** Cross-sectional meta-analysis of studies reporting fasting glucose in post-treatment female anorexia nervosa patients compared with healthy controls. Three samples had the appropriate data for the meta-analysis with 45 AN cases and 41 controls. A random-effects meta-analysis revealed a pooled estimate of the mean difference (MD: -1.78 mg/dL; 95% CI: -8.98, 5.42; *P* = 0.63) with the mean differences ranging from -8.83 mg/dL to 5.41 mg/dL. Heterogeneity between studies was statistically significant (*τ^2^* = 28.75; *P* = 0.05; *I^2^* = 66.9%).


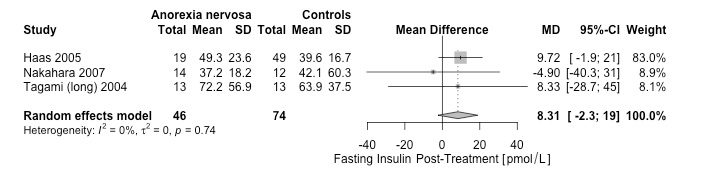


**Figure S50.** Cross-sectional meta-analysis of studies reporting fasting insulin in post-treatment female anorexia nervosa patients compared with healthy controls. Three samples had the appropriate data for the meta-analysis with 46 AN cases and 74 controls. A random-effects meta-analysis revealed a pooled estimate of the mean difference (MD: 8.31 pmol/L; 95% CI: -2.26, 18.87; *P* = 0.12) with the mean differences ranging from -4.90 pmol/L to 9.72 pmol/L. There was no Heterogeneity between studies (*τ^2^* = 0.00; *P* = 0.74; *I^2^* = 0.0%). Long, longitudinal.


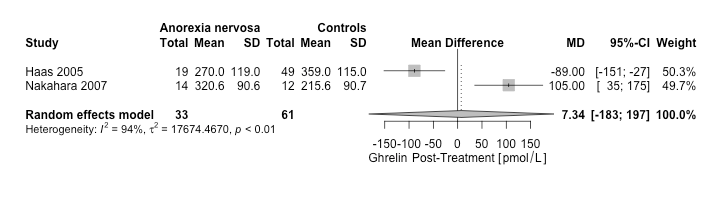


**Figure S51.** Cross-sectional meta-analysis of studies reporting ghrelin in post-treatment female anorexia nervosa patients compared with healthy controls. Two samples had the appropriate data for the meta-analysis with 33 AN cases and 61 controls. A random-effects meta-analysis revealed a pooled estimate of the mean difference (MD: 7.34 pmol/L; 95% CI: -182.77, 197.45; *P* = 0.94) with the mean differences ranging from -89.00 pmol/L to 105.00 pmol/L. Heterogeneity between studies was statistically significant (*τ^2^* = 17674.47; *P* = 4.98 x 10^-5^ ; *I^2^* = 93.9%).


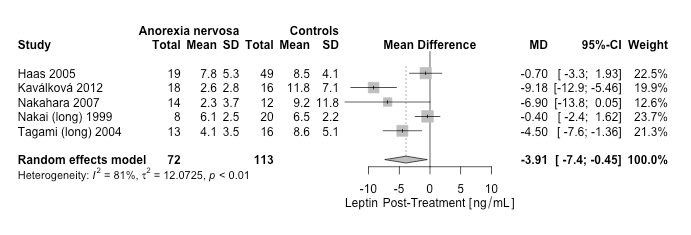


**Figure S52.** Cross-sectional meta-analysis of studies reporting leptin in post-treatment female anorexia nervosa patients compared with healthy controls. Five samples had the appropriate data for the meta-analysis with 72 AN cases and 113 controls. A random-effects meta-analysis revealed a pooled estimate of the mean difference (MD: -3.91 ng/mL; 95% CI: -7.37, -0.45; *P* = 0.03) with the mean differences ranging from -9.18 ng/mL to -0.40 ng/mL. Heterogeneity between studies was statistically significant (*τ^2^* =12.07; *P* = 2.59 x 10^-4^ ; *I^2^* = 81.3%). Long, longitudinal.


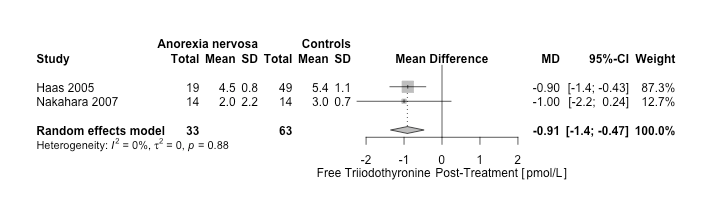


**Figure S53.** Cross-sectional meta-analysis of studies reporting free triiodothyronine in post-treatment female anorexia nervosa patients compared with healthy controls. Two samples had the appropriate data for the meta-analysis with 33 AN cases and 63 controls. A random-effects meta-analysis revealed a pooled estimate of the mean difference (MD: -0.91 pmol/L; 95% CI: -1.36, -0.47; *P* = 5.26 x 10^-5^) with the mean differences ranging from -1.00 pmol/L to -0.90 pmol/L. There was no Heterogeneity between studies (*τ^2^* = 0.00; *P* = 0.88; *I^2^* = 0.0%).

**7) Longitudinal meta-analyses of studies comparing anorexia nervosa patients pre- and post-treatment**


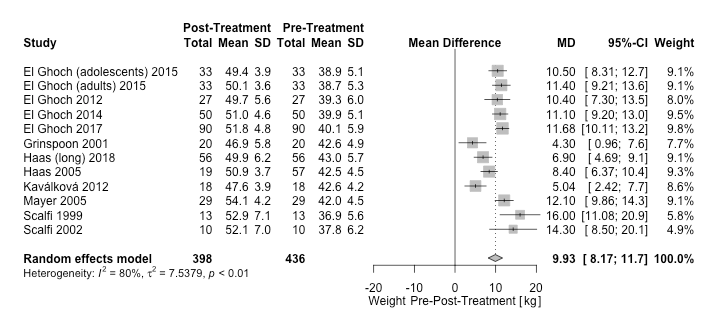


**Figure S54.** Longitudinal meta-analysis of studies reporting weight in female anorexia nervosa patients pre- and post-treatment. Twelve samples had the appropriate data for the meta-analysis with 398 AN cases and 436 controls. A random-effects meta-analysis revealed a pooled estimate of the mean difference (MD: 9.93 kg; 95% CI: 8.17, 11.68; *P* = 1.44 x 10^-28^) with the mean differences ranging from 4.30 kg to 16.00 kg. Heterogeneity between studies was statistically highly significant (*τ^2^* = 7.54; *P* = 1.35 x 10^-7^; *I^2^* = 80.0%). C, subtype-combined sample.


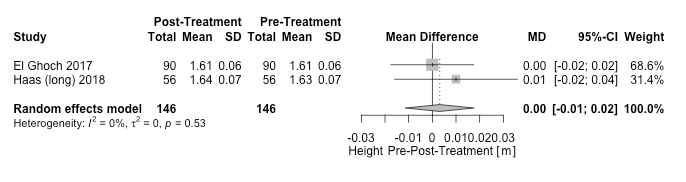


**Figure S55.** Longitudinal meta-analysis of studies reporting height in female anorexia nervosa patients pre- and post-treatment. Two samples had the appropriate data for the meta-analysis with 146 AN cases and 146 controls. A random-effects meta-analysis revealed a pooled estimate of the mean difference (MD: 0.00 m; 95% CI: -0.01, 0.02; *P* = 0.67) with the mean differences ranging from 0.00 m to 0.01 m. There was no Heterogeneity between studies (*τ^2^* = 0.00; *P* = 0.53; *I^2^* = 0.0%). C, subtype-combined sample.


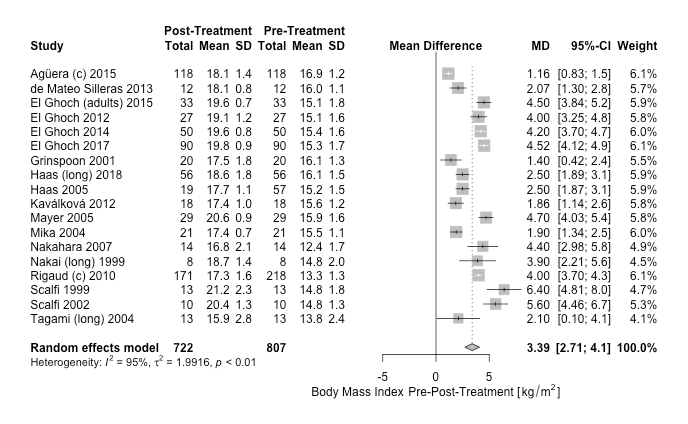


**Figure S56.** Longitudinal meta-analysis of studies reporting body mass index in female anorexia nervosa patients pre- and post-treatment. Eighteen samples had the appropriate data for the meta-analysis with 722 AN cases and 807 controls. A random-effects meta-analysis revealed a pooled estimate of the mean difference (MD: 3.39 kg/m^2^ kg; 95% CI: 2.71, 4.08; *P* = 4.19 x 10^-22^) with the mean differences ranging from 1.16 kg/m^2^ to 6.40 kg/m^2^. Heterogeneity between studies was statistically highly significant (*τ^2^* = 1.99; *P* = 8.54 x 10^-68^; *I^2^* = 95.0%). C, subtype-combined sample; long, longitudinal.


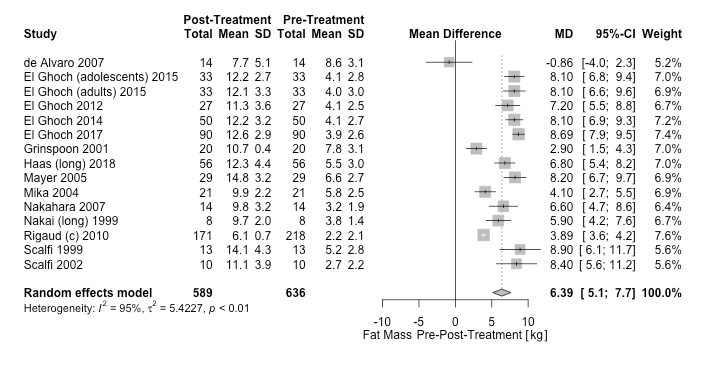


**Figure S57.** Longitudinal meta-analysis of studies reporting fat mass in female anorexia nervosa patients pre- and post-treatment. Fifteen samples had the appropriate data for the meta-analysis with 589 AN cases and 636 controls. A random-effects meta-analysis revealed a pooled estimate of the mean difference (MD: 6.39 kg; 95% CI: 5.13, 7.65; *P* = 2.79 x 10^-23^) with the mean differences ranging from -0.86 kg to 8.90 kg. Heterogeneity between studies was statistically highly significant (*τ^2^* = 5.42; *P* = 7.66 x 10^-49^; *I^2^* = 95.0%). C, subtype-combined sample.


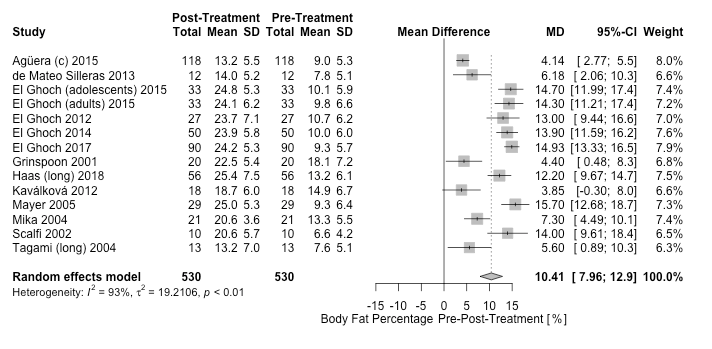


**Figure S58.** Longitudinal meta-analysis of studies reporting body fat percentage in female anorexia nervosa patients pre- and post-treatment. Fourteen samples had the appropriate data for the meta-analysis with 530 AN cases and 530 controls. A random-effects meta-analysis revealed a pooled estimate of the mean difference (MD: 10.4%; 95% CI: 7.96, 12.87; *P* = 9.23 x 10^-17^) with the mean differences ranging from 3.9% to 15.7%. Heterogeneity between studies was statistically highly significant (*τ^2^* = 19.21; *P* = 2.07 x 10^-32^; *I^2^* = 93.0%). C, subtype-combined sample; long, longitudinal.


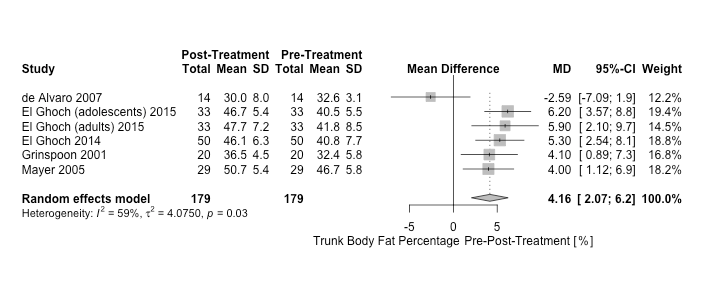


**Figure S59.** Longitudinal meta-analysis of studies reporting trunk body fat percentage in female anorexia nervosa patients pre- and post-treatment. Six samples had the appropriate data for the meta-analysis with 179 AN cases and 179 controls. A random-effects meta-analysis revealed a pooled estimate of the mean difference (MD: 4.2%; 95% CI: 2.07, 6.25; *P* = 9.67 x 10^-5^) with the mean differences ranging from -2.6% to 6.2%. Heterogeneity between studies was statistically significant (*τ^2^* = 4.08; *P* = 0.03; *I^2^* = 59.0%).


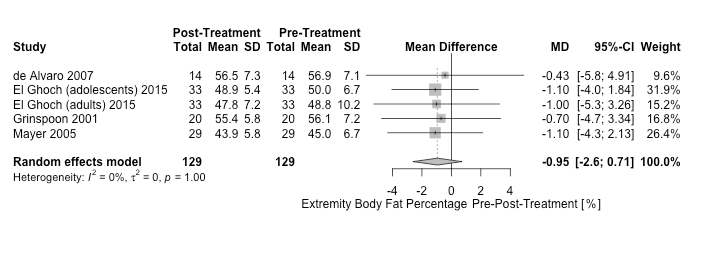


**Figure S60.** Longitudinal meta-analysis of studies reporting extremity body fat percentage in female anorexia nervosa patients pre- and post-treatment. Five samples had the appropriate data for the meta-analysis with 129 AN cases and 129 controls. A random-effects meta-analysis revealed a pooled estimate of the mean difference (MD: -1.0%; 95% CI: -2.61, 0.71; *P* = 0.26) with the mean differences ranging from -1.1% to -0.4%. There was no Heterogeneity between studies (*τ^2^* = 0.00; *P* = 1.00; *I^2^* = 0.0%).


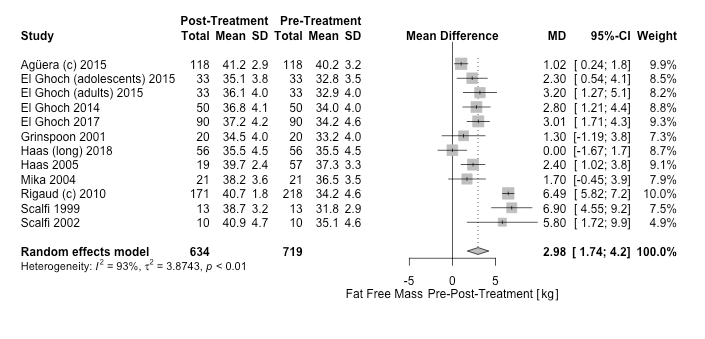


**Figure S61.** Longitudinal meta-analysis of studies reporting fat free mass in female anorexia nervosa patients pre- and post-treatment. Twelve samples had the appropriate data for the meta-analysis with 634 AN cases and 719 controls. A random-effects meta-analysis revealed a pooled estimate of the mean difference (MD: 2.98 kg; 95% CI: 1.74, 4.22; *P* = 2.35 x 10^-6^) with the mean differences ranging from 0.00 kg to 6.90 kg. Heterogeneity between studies was statistically highly significant (*τ^2^* = 3.87; *P* = 4.57x 10^-27^; *I^2^* = 93.0%). C, subtype-combined sample.


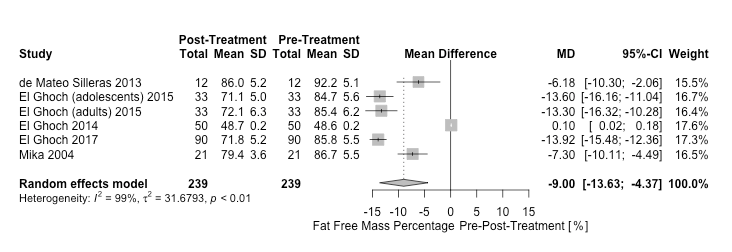


**Figure S62.** Longitudinal meta-analysis of studies reporting fat free mass percentage in female anorexia nervosa patients pre- and post-treatment. Six samples had the appropriate data for the meta-analysis with 239 AN cases and 239 controls. A random-effects meta-analysis revealed a pooled estimate of the mean difference (MD: -9.0%; 95% CI: -13.6, -4.4; *P* = 1.38 x 10^-4^) with the mean differences ranging from -13.9% to 0.1%. Heterogeneity between studies was statistically highly significant (*τ^2^* = 31.68; *P* = 9.94 x 10^-112^; *I^2^* = 99.0%).


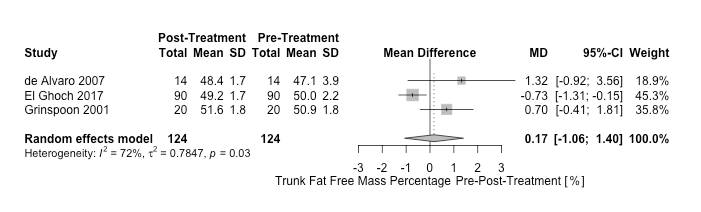


**Figure S63.** Longitudinal meta-analysis of studies reporting trunk fat free mass percentage in female anorexia nervosa patients pre- and post-treatment. Three samples had the appropriate data for the meta-analysis with 124 AN cases and 124 controls. A random-effects meta-analysis revealed a pooled estimate of the mean difference (MD: 0.2%; 95% CI: -1.1, 1.4; *P* = 0.79) with the mean differences ranging from -0.7% to 1.3%. Heterogeneity between studies was statistically significant (*τ^2^* = 0.78; *P* = 0.03; *I^2^* = 72.3%).


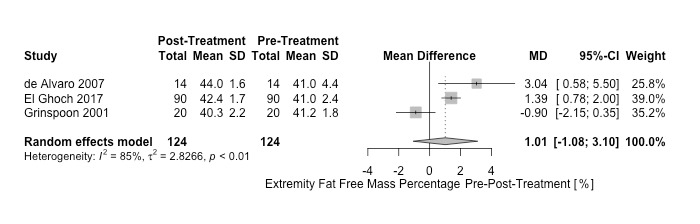


**Figure S64.** Longitudinal meta-analysis of studies reporting extremity fat free mass percentage in female anorexia nervosa patients pre- and post-treatment. Three samples had the appropriate data for the meta-analysis with 124 AN cases and 124 controls. A random-effects meta-analysis revealed a pooled estimate of the mean difference (MD: 1.0%; 95% CI: -1.08, 3.10; *P* = 0.34) with the mean differences ranging from -0.9% to 3.0%. Heterogeneity between studies was statistically significant (*τ^2^* = 2.83; *P* = 1.51 x 10^-3^; *I^2^* = 84.6%).


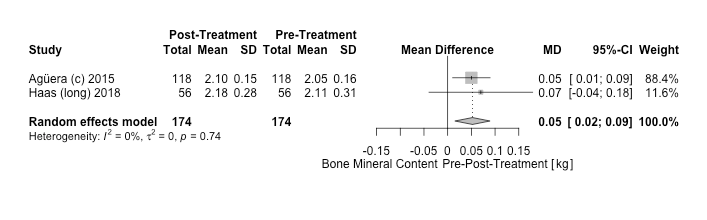


**Figure S65.** Longitudinal meta-analysis of studies reporting bone mineral content in female anorexia nervosa patients pre- and post-treatment. Two samples had the appropriate data for the meta-analysis with 174 AN cases and 174 controls. A random-effects meta-analysis revealed a pooled estimate of the mean difference (MD: 0.05 kg; 95% CI: 0.02, 0.09; *P* = 5.86 x 10^-3^) with the mean differences ranging from 0.05 kg to 0.07 kg. There was no Heterogeneity between studies (*τ^2^* = 0.00; *P* = 0.74; *I^2^* = 0.0%). C, subtype-combined sample; long, longitudinal.


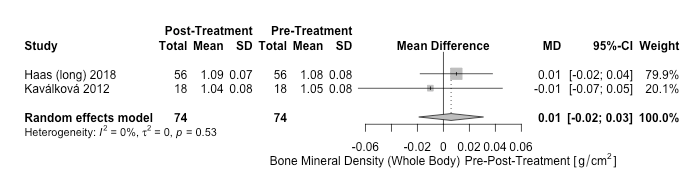


**Figure S66.** Longitudinal meta-analysis of studies reporting bone mineral density (whole body) in female anorexia nervosa patients pre- and post-treatment. Two samples had the appropriate data for the meta-analysis with 74 AN cases and 74 controls. A random-effects meta-analysis revealed a pooled estimate of the mean difference (MD: 0.01 g/cm^2^; 95% CI: -0.02, 0.03; *P* = 0.64) with the mean differences ranging from -0.01 g/cm^2^ to 0.01 g/cm^2^. There was no Heterogeneity between studies (*τ^2^* = 0.00; *P* = 0.53; *I^2^* = 0.0%). Long, longitudinal.


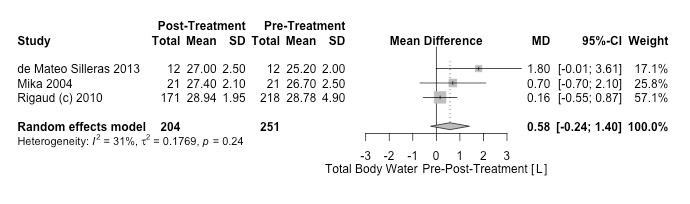


**Figure S67.** Longitudinal meta-analysis of studies reporting total body water in female anorexia nervosa patients pre- and post-treatment. Three samples had the appropriate data for the meta-analysis with 204 AN cases and 251 controls. A random-effects meta-analysis revealed a pooled estimate of the mean difference (MD: 0.58 L; 95% CI: -0.24, 1.40; *P* = 0.17) with the mean differences ranging from 0.16 L to 1.80 L. Heterogeneity between studies was not statistically significant (*τ^2^* = 0.18; *P* = 0.24; *I^2^* = 30.8%). C, subtype-combined sample.


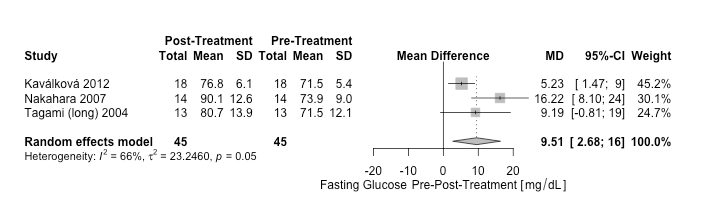


**Figure S68.** Longitudinal meta-analysis of studies reporting fasting glucose in female anorexia nervosa patients pre- and post-treatment. Three samples had the appropriate data for the meta-analysis with 45 AN cases and 45 controls. A random-effects meta-analysis revealed a pooled estimate of the mean difference (MD: 9.51 mg/dL; 95% CI: 2.68, 16.35; *P* = 6.38 x 10^-3^) with the mean differences ranging from 5.23 mg/dL to 16.22 mg/dL. Heterogeneity between studies was statistically significant (*τ^2^* = 23.25; *P* = 0.05; *I^2^* = 66.3%). Long, longitudinal.


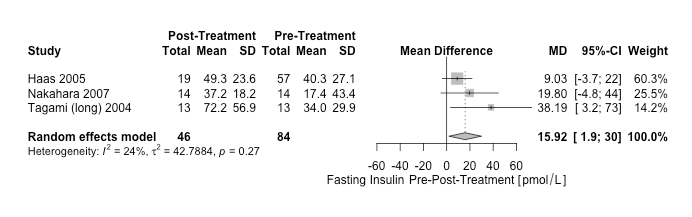


**Figure S69.** Longitudinal meta-analysis of studies reporting fasting insulin in female anorexia nervosa patients pre- and post-treatment. Three samples had the appropriate data for the meta-analysis with 45 AN cases and 45 controls. A random-effects meta-analysis revealed a pooled estimate of the mean difference (MD: 15.92 pmol/L; 95% CI: 1.89, 29.95; *P* = 0.03) with the mean differences ranging from 9.03 pmol/L to 38.19 pmol/L. Heterogeneity between studies was not statistically significant (*τ^2^* = 42.79; *P* = 0.27) or large in magnitude (*I^2^* = 24.2%). Long, longitudinal.


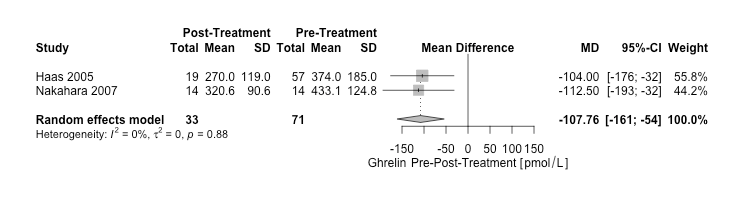


**Figure S70.** Longitudinal meta-analysis of studies reporting ghrelin in female anorexia nervosa patients pre- and post-treatment. Two samples had the appropriate data for the meta-analysis with 33 AN cases and 71 controls. A random-effects meta-analysis revealed a pooled estimate of the mean difference (MD: -107.76 pmol/L; 95% CI: -161.47, -54.05; *P* = 8.41 x 10^-5^) with the mean differences ranging from -112.50 pmol/L to -104.00 pmol/L. There was no Heterogeneity between studies (*τ^2^* = 0.00; *P* = 0.88; *I^2^* = 0.0%).


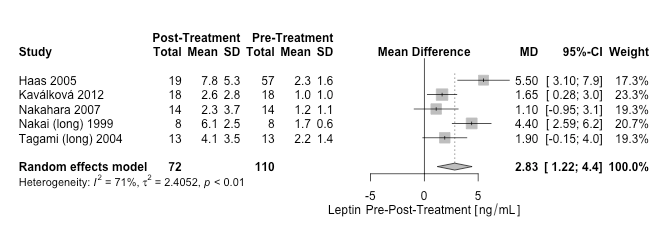


**Figure S71.** Longitudinal meta-analysis of studies reporting leptin in female anorexia nervosa patients pre- and post-treatment. Five samples had the appropriate data for the meta-analysis with 72 AN cases and 110 controls. A random-effects meta-analysis revealed a pooled estimate of the mean difference (MD: 2.83 ng/mL; 95% CI: 1.22, 4.44; *P* = 5.79 x 10^-4^) with the mean differences ranging from 1.10 ng/mL to 5.50 ng/mL. Heterogeneity between studies was not statistically significant (*τ^2^* = 2.41; *P* = 8.06 x 10^-3^; *I^2^* = 71.0%). Long, longitudinal.


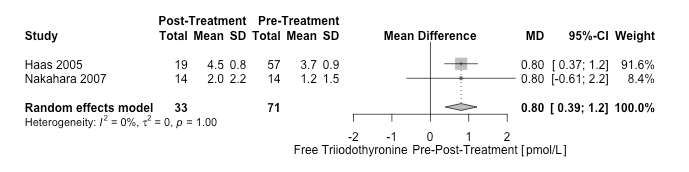


**Figure S72.** Longitudinal meta-analysis of studies reporting free triiodothyronine in female anorexia nervosa patients pre- and post-treatment. Two samples had the appropriate data for the meta-analysis with 33 AN cases and 71 controls. A random-effects meta-analysis revealed a pooled estimate of the mean difference (MD: 0.80 pmol/L; 95% CI: 0.39, 1.21; *P* = 1.33 x 10^-4^) with the mean differences ranging from 0.80 pmol/L to 0.80 pmol/L. There was no Heterogeneity between studies (*τ^2^* = 0.00; *P* = 1.00; *I^2^* = 0.0%).

**8) Cross-sectional meta-analyses of studies comparing weight-recovered anorexia nervosa patients with healthy controls**


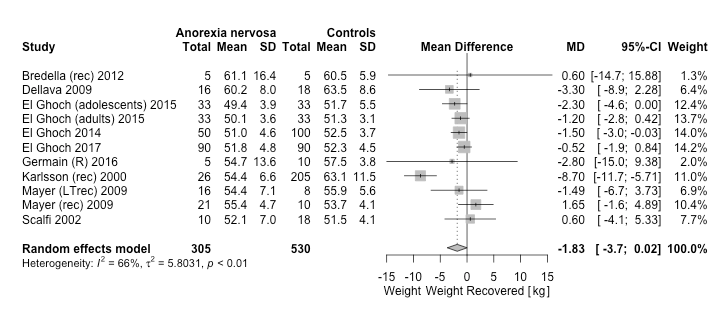


**Figure S73.** Cross-sectional meta-analysis of studies reporting weight in weight-recovered female anorexia nervosa patients compared with healthy controls. Eleven samples had the appropriate data for the meta-analysis with 305 AN cases and 530 controls. A random-effects meta-analysis revealed a pooled estimate of the mean difference (MD: -1.83 kg; 95% CI: -3.68, 0.02; *P* = 0.05) with the mean differences ranging from -8.70 kg to 1.65 kg. Heterogeneity between studies was statistically significant (*τ^2^* = 5.80; *P* = 9.43 x 10^-4^; *I^2^* = 66.4%). C, subtype-combined sample; rec, recovered; LTrec, long term weight-recovered.

**
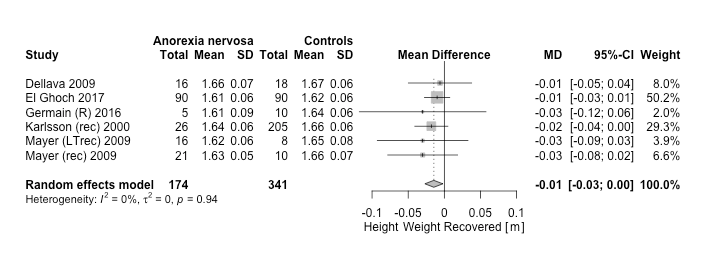
**

**Figure S74.** Cross-sectional meta-analysis of studies reporting height in weight-recovered female anorexia nervosa patients compared with healthy controls. Six samples had the appropriate data for the meta-analysis with 174 AN cases and 341 controls. A random-effects meta-analysis revealed a pooled estimate of the mean difference (MD: -0.01 m; 95% CI: -0.03, 0.00; *P* = 0.02) with the mean differences ranging from -0.03 m to -0.01 m. There was no Heterogeneity between studies (*τ^2^* = 0.00; *P* = 0.94; *I^2^* = 0.0%). R, restricting; Rec, weight-recovered; LTrec, long term weight-recovered.

**
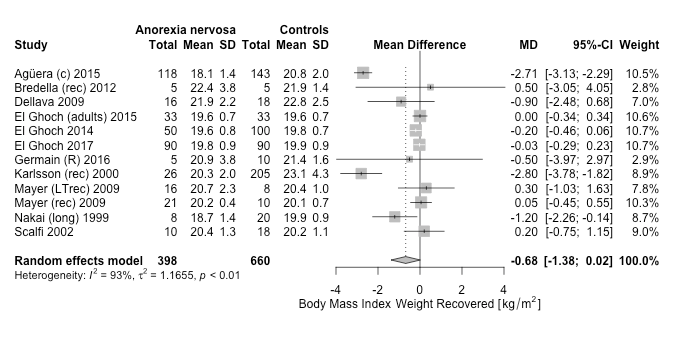
**

**Figure S75.** Cross-sectional meta-analysis of studies reporting body mass index in weight-recovered female anorexia nervosa patients compared with healthy controls. Twelve samples had the appropriate data for the meta-analysis with 398 AN cases and 660 controls. A random-effects meta-analysis revealed a pooled estimate of the mean difference (MD: -0.68 kg/m^2^; 95% CI: -1.38, 0.02; *P* = 6.76 x 10^-3^) with the mean differences ranging from -2.80 kg/m^2^ to 0.50 kg/m^2^. Heterogeneity between studies was statistically highly significant (*τ^2^* = 1.17; *P* = 1.16 x 10^-29^; *I^2^* = 93.3%). C, subtype-combined sample; long, longitudinal; rec, weight-recovered; LTrec, long term weight-recovered.

**
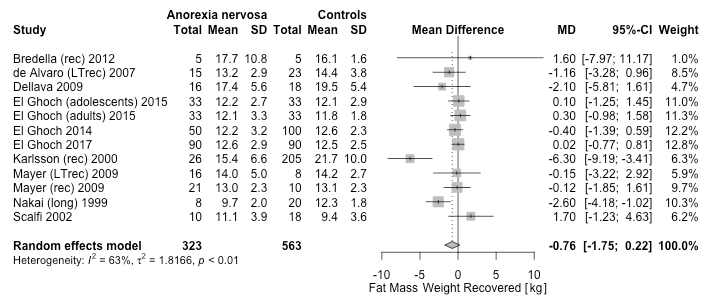
**

**Figure S76.** Cross-sectional meta-analysis of studies reporting fat mass in weight-recovered female anorexia nervosa patients compared with healthy controls. Twelve samples had the appropriate data for the meta-analysis with 323 AN cases and 563 controls. A random-effects meta-analysis revealed a pooled estimate of the mean difference (MD: -0.76 kg; 95% CI: -1.75, 0.22; *P* = 013) with the mean differences ranging from -6.30 kg to 1.70 kg. Heterogeneity between studies was statistically significant (*τ^2^* = 1.82; *P* = 0.00; *I^2^* = 63.0%). Long, longitudinal; rec, weight-recovered; LTrec, long term weight-recovered.


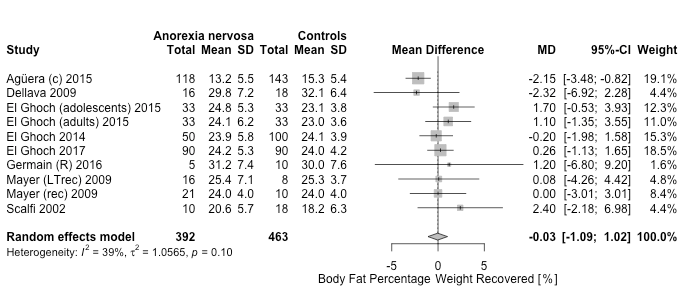


**Figure S77.** Cross-sectional meta-analysis of studies reporting body fat percentage in weight-recovered female anorexia nervosa patients compared with healthy controls. Ten samples had the appropriate data for the meta-analysis with 392 AN cases and 463 controls. A random-effects meta-analysis revealed a pooled estimate of the mean difference (MD: -0.03%; 95% CI: -1.09, 1.02; *P* = 0.95) with the mean differences ranging from -2.32% to 2.40%. Heterogeneity between studies was not statistically significant (*τ^2^* = 1.06; *P* = 0.10) or large in magnitude (*I^2^* = 39.0%). C, subtype-combined sample; rec, weight-recovered, LTrec, long term weight-recovered.


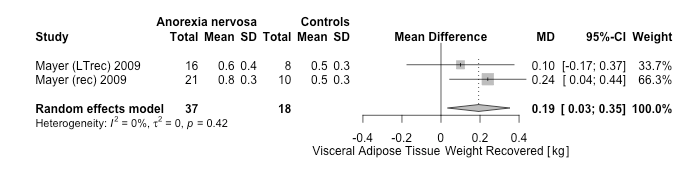


**Figure S78.** Cross-sectional meta-analysis of studies reporting visceral adipose tissue in weight-recovered female anorexia nervosa patients compared with healthy controls. Two samples had the appropriate data for the meta-analysis with 37 AN cases and 18 controls. A random-effects meta-analysis revealed a pooled estimate of the mean difference (MD: 0.19 kg; 95% CI: 0.03, 0.35; *P* = 0.02) with the mean differences ranging from 0.10 kg to 0.24 kg. There was no Heterogeneity between studies (*τ^2^* = 0.00; *P* = 0.42; *I^2^* = 0.0%). Rec, weight-recovered; LTrec, long term weight-recovered.


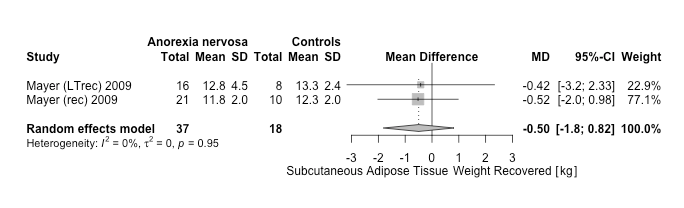


**Figure S79.** Cross-sectional meta-analysis of studies reporting subcutaneous adipose tissue in weight-recovered female anorexia nervosa patients compared with healthy controls. Two samples had the appropriate data for the meta-analysis with 37 AN cases and 18 controls. A random-effects meta-analysis revealed a pooled estimate of the mean difference (MD: -0.50 kg; 95% CI: -1.81, 0.82; *P* = 0.46) with the mean differences ranging from -0.52 kg to -0.42 kg. There was no Heterogeneity between studies (*τ^2^* = 0.00; *P* = 0.95; *I^2^* = 0.0%). Rec, weight-recovered; LTrec, long term weight-recovered.


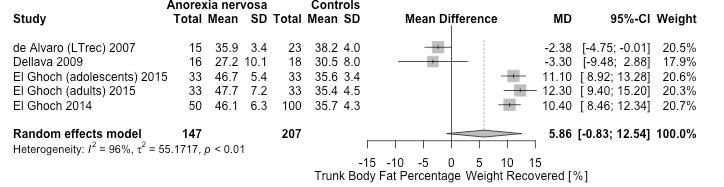


**Figure S80.** Cross-sectional meta-analysis of studies reporting trunk body fat percentage in weight-recovered female anorexia nervosa patients compared with healthy controls. Five samples had the appropriate data for the meta-analysis with 147 AN cases and 207 controls. A random-effects meta-analysis revealed a pooled estimate of the mean difference (MD: 5.9%; 95% CI: -0.83, 12.54; *P* = 0.09) with the mean differences ranging from -3.3% to 12.3%. Heterogeneity between studies was statistically highly significant (*τ^2^* = 55.17; *P* = 1.75 x 10^-22^; *I^2^* = 96.3%). LTrec, long term weight-recovered.


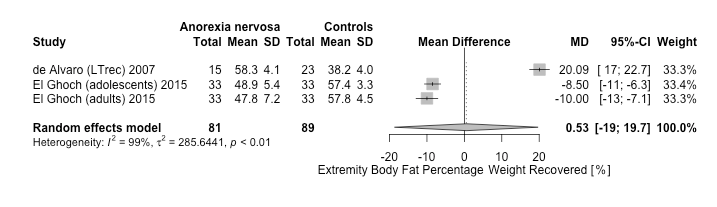


**Figure S81.** Cross-sectional meta-analysis of studies reporting extremity body fat percentage in weight-recovered female anorexia nervosa patients compared with healthy controls. Three samples had the appropriate data for the meta-analysis with 81 AN cases and 89 controls. A random-effects meta-analysis revealed a pooled estimate of the mean difference (MD: 0.5%; 95% CI: -18.65, 19.71; *P* = 0.96) with the mean differences ranging from -10.0% to 20.%. Heterogeneity between studies was statistically highly significant (*τ^2^* = 285.64; *P* = 7.45 x 10^-72^; *I^2^* = 99.4%). LTrec, long term weight-recovered.


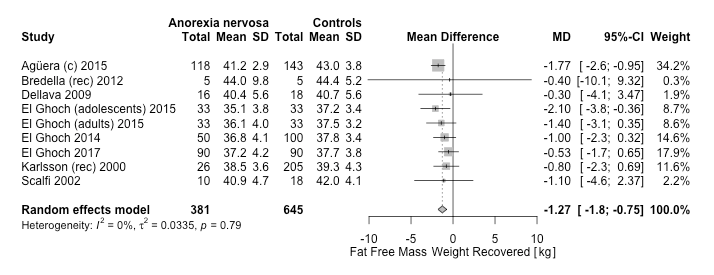


**Figure S82.** Cross-sectional meta-analysis of studies reporting fat free mass in weight-recovered female anorexia nervosa patients compared with healthy controls. Nine samples had the appropriate data for the meta-analysis with 381 AN cases and 645 controls. A random-effects meta-analysis revealed a pooled estimate of the mean difference (MD: -1.27 kg; 95% CI: -1.80, -0.75; *P* = 1.81 x 10^-6^) with the mean differences ranging from -2.10 kg to -0.30 kg. There was no Heterogeneity between studies (*τ^2^* = 0.03; *P* = 0.79; *I^2^* = 0.0%). C, subtype-combined sample; rec, weight-recovered.


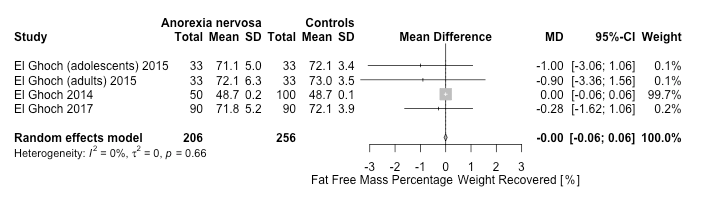


**Figure S83.** Cross-sectional meta-analysis of studies reporting fat free mass percentage in weight-recovered female anorexia nervosa patients compared with healthy controls. Four samples had the appropriate data for the meta-analysis with 206 AN cases and 256 controls. A random-effects meta-analysis revealed a pooled estimate of the mean difference (MD: -0.0%; 95% CI: -0.06, 0.06; *P* = 0.95) with the mean differences ranging from -1.0% to 0.0%. There was no Heterogeneity between studies (*τ^2^* = 0.00; *P* = 0.66; *I^2^* = 0.0%).


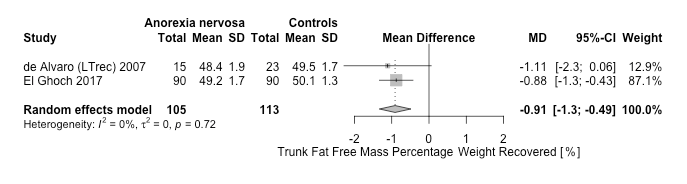


**Figure S84.** Cross-sectional meta-analysis of studies reporting trunk fat free mass percentage in weight-recovered female anorexia nervosa patients compared with healthy controls. Two samples had the appropriate data for the meta-analysis with 105 AN cases and 113 controls. A random-effects meta-analysis revealed a pooled estimate of the mean difference (MD: -0.9%; 95% CI: -1.33, -0.49; *P* = 2.29 x 10^-5^) with the mean differences ranging from -1.1% to -0.9%. There was no Heterogeneity between studies (*τ^2^* = 0.00; *P* = 0.72; *I^2^* = 0.0%). LTrec, long term weight-recovered.


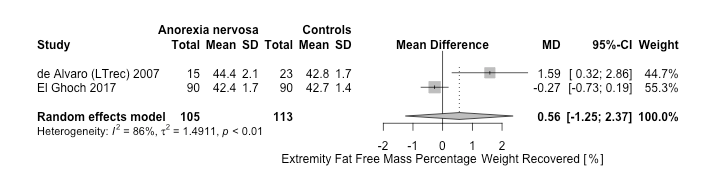


**Figure S85.** Cross-sectional meta-analysis of studies reporting extremity fat free mass percentage in weight-recovered female anorexia nervosa patients compared with healthy controls. Two samples had the appropriate data for the meta-analysis with 105 AN cases and 113 controls. A random-effects meta-analysis revealed a pooled estimate of the mean difference (MD: 0.6%; 95% CI: -1.25, 2.37; *P* = 0.54) with the mean differences ranging from -0.3% to 1.6%. Heterogeneity between studies was statistically significant (*τ^2^* = 1.49; *P* = 7.10 x 10^-3^; *I^2^* = 86.2%). LTrec, long term weight-recovered.


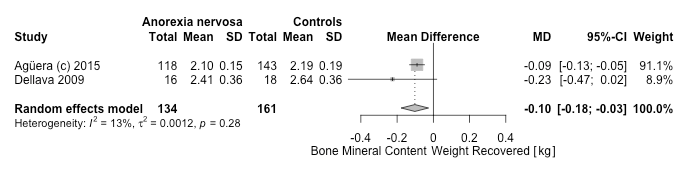


**Figure S86.** Cross-sectional meta-analysis of studies reporting bone mineral content in weight-recovered female anorexia nervosa patients compared with healthy controls. Two samples had the appropriate data for the meta-analysis with 134 AN cases and 161 controls. A random-effects meta-analysis revealed a pooled estimate of the mean difference (MD: -0.10 kg; 95% CI: -0.18, -0.03; *P* = 0.01) with the mean differences ranging from -0.23 kg to -0.09 kg. There was no Heterogeneity between studies (*τ^2^* = 0.00; *P* = 0.28; *I^2^* = 13.0%). C, subtype-combined sample.


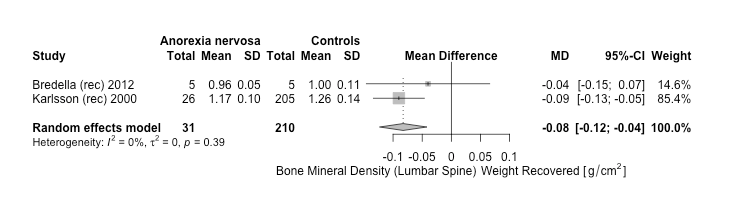


**Figure S87.** Cross-sectional meta-analysis of studies reporting bone mineral density (lumbar spine) in weight-recovered female anorexia nervosa patients compared with healthy controls. Two samples had the appropriate data for the meta-analysis with 31 AN cases and 210 controls. A random-effects meta-analysis revealed a pooled estimate of the mean difference (MD: -0.08 g/cm^2^; 95% CI: -0.12, -0.04; *P* = 6.28 x 10^-5^) with the mean differences ranging from -0.09 g/cm^2^ to -0.04 g/cm^2^. There was no Heterogeneity between studies (*τ^2^* = 0.00; *P* = 0.39; *I^2^* = 0.0%). Rec, weight-recovered.


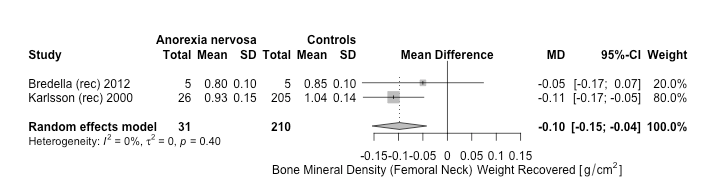


**Figure S88.** Cross-sectional meta-analysis of studies reporting bone mineral density (femoral neck) in weight-recovered female anorexia nervosa patients compared with healthy controls. Two samples had the appropriate data for the meta-analysis with 31 AN cases and 210 controls. A random-effects meta-analysis revealed a pooled estimate of the mean difference (MD: -0.10 g/cm^2^; 95% CI: -0.15, -0.04; *P* = 5.31 x 10^-4^) with the mean differences ranging from -0.11 g/cm^2^ to -0.05 g/cm^2^. There was no Heterogeneity between studies (*τ^2^* = 0.00; *P* = 0.40; *I^2^* = 0.0%). Rec, weight-recovered.


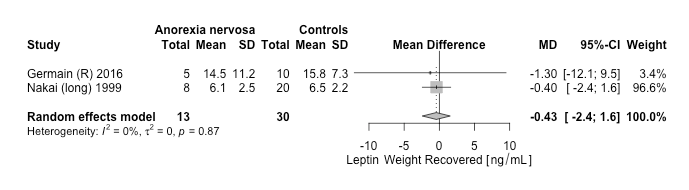


**Figure S89.** Cross-sectional meta-analysis of studies reporting leptin in weight-recovered female anorexia nervosa patients compared with healthy controls. Two samples had the appropriate data for the meta-analysis with 13 AN cases and 30 controls. A random-effects meta-analysis revealed a pooled estimate of the mean difference (MD: -0.43 ng/mL; 95% CI: -2.41, 1.55; *P* = 0.67) with the mean differences ranging from -1.30 ng/mL to -0.40 ng/mL. There was no Heterogeneity between studies (*τ^2^* = 0.00; *P* = 0.87; *I^2^* = 0.0%). Long, longitudinal R, restricting.

**
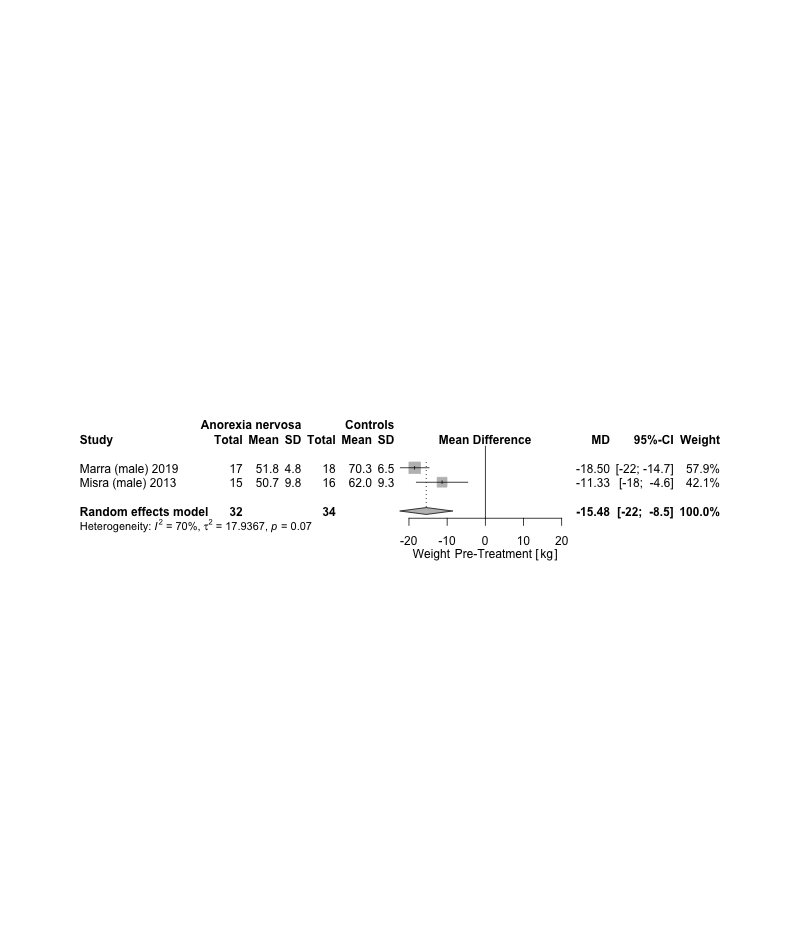
**

**Figure S90.** Cross-sectional meta-analysis of studies reporting weight in acutely-ill/pre-treatment male anorexia nervosa patients compared with healthy controls. Two samples had the appropriate data for the meta-analysis with 32 AN cases and 34 controls. A random-effects meta-analysis revealed a pooled estimate of the mean difference (MD: -15.48 kg; % CI: -22.42, -8.54; *P* = 1.22 x 10^-5^) with the mean differences ranging from -18.50 kg to -11.33 kg. Heterogeneity between studies was not statistically significant (*τ^2^* = 17.94; *P* = 0.07; *I^2^* = 69.80%).


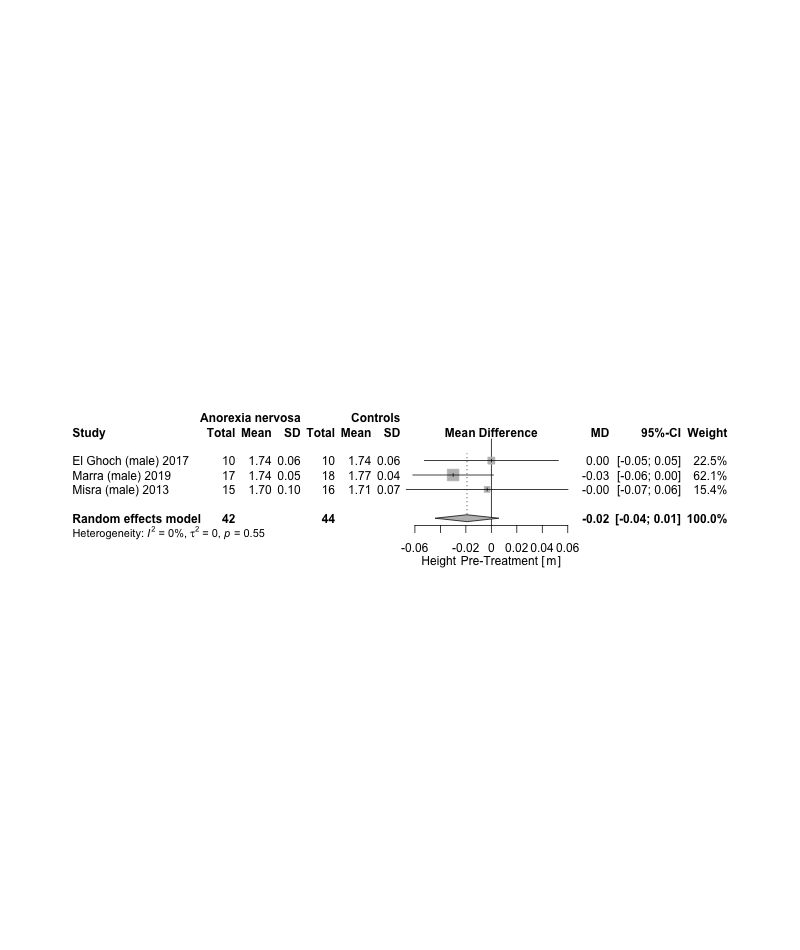


**Figure S91.** Cross-sectional meta-analysis of studies reporting height in acutely-ill/pre-treatment male anorexia nervosa patients compared with healthy controls. Three samples had the appropriate data for the meta-analysis with 42 AN cases and 44 controls. A random-effects meta-analysis revealed a pooled estimate of the mean difference (MD: -0.02 m; 95% CI: -0.04, 0.01; *P* = 0.13) with the mean differences ranging from -0.03 m to 0.00 m. Heterogeneity between studies was not statistically significant (*τ^2^* = 0; *P* = 0.55; *I^2^* = 0%). C, subtype-combined sample.

**
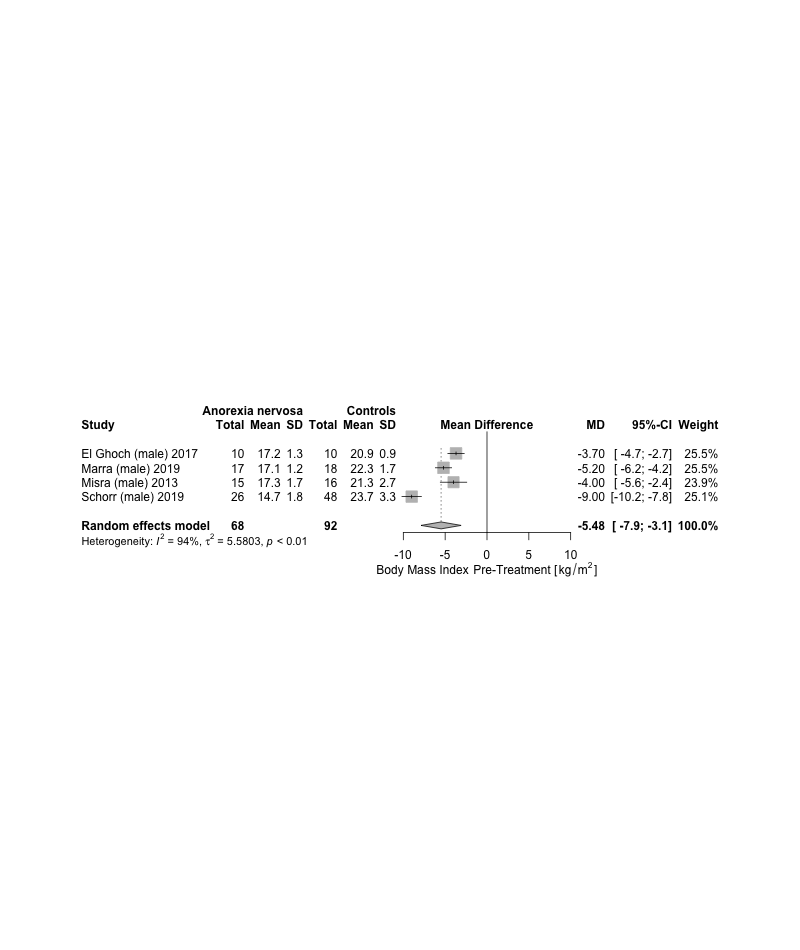
**

**Figure S92.** Cross-sectional meta-analysis of studies reporting body mass index in acutely-ill/pre-treatment male anorexia nervosa patients compared with healthy controls. Four samples had the appropriate data for the meta-analysis with 68 AN cases and 92 controls. A random-effects meta-analysis revealed a pooled estimate of the mean difference (MD: -5.48 kg/m^2^; 95% CI: -7.87, -3.09; *P* = 6.92 x 10^-6^) with the mean differences ranging from -9.00 kg/m^2^ to -3.70 kg/m^2^. Heterogeneity between studies was statistically highly significant (*τ^2^* = 5.58; *P* = 3.65 x 10^-11^; *I^2^* = 94.2%).

**
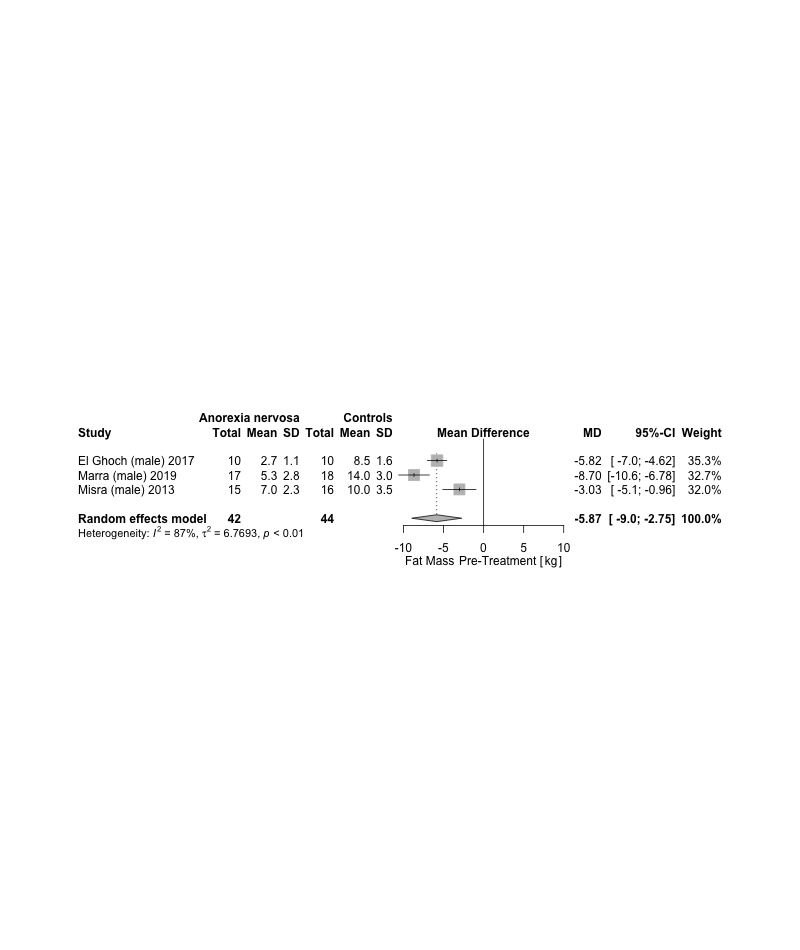
**

**Figure S93.** Cross-sectional meta-analysis of studies reporting fat mass in acutely-ill/pre-treatment male anorexia nervosa patients compared with healthy controls. Three samples had the appropriate data for the meta-analysis with 42 AN cases and 44 controls. A random-effects meta-analysis revealed a pooled estimate of the mean difference (MD: -5.87 kg; 95% CI: -8.98, -2.75; *P* = 2.22 x 10^-4^) with the mean differences ranging from -8.70 kg to -3.03 kg. Heterogeneity between studies was statistically highly significant (*τ^2^* =6.77; *P* = 4.17 x 10^-4^; *I^2^* = 87.20%). C, subtype-combined sample.


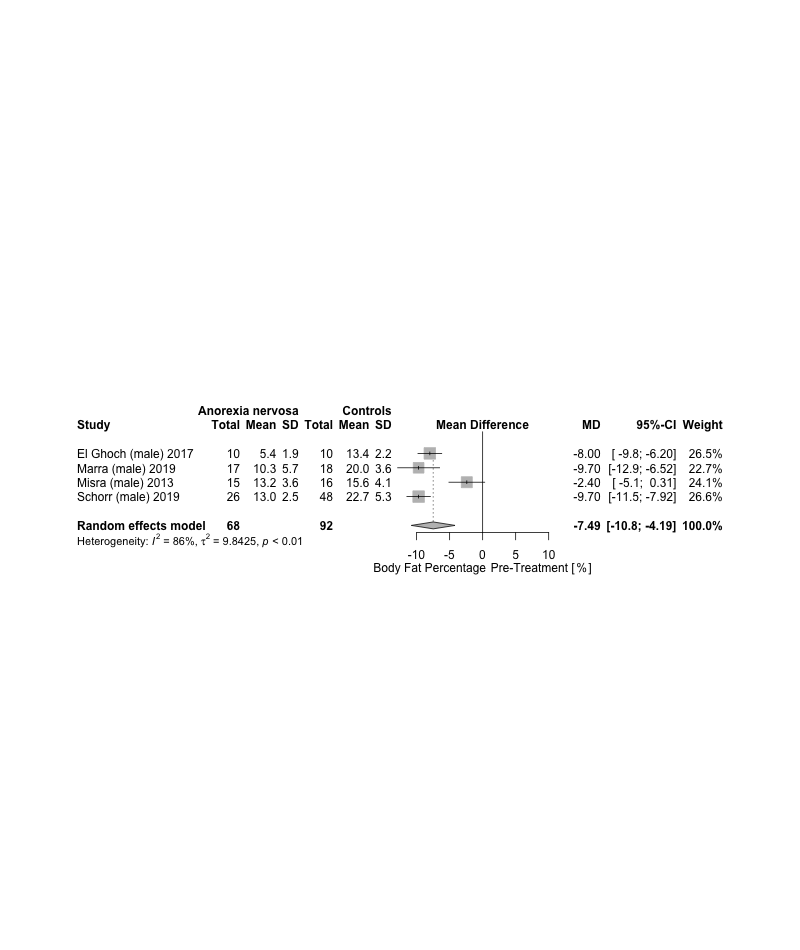


**Figure S94.** Cross-sectional meta-analysis of studies reporting body fat percentage in acutely-ill/pre-treatment male anorexia nervosa patients compared with healthy controls. Four samples had the appropriate data for the meta-analysis with 68 AN cases and 92 controls. A random-effects meta-analysis revealed a pooled estimate of the mean difference (MD: -7.49%; 95% CI: -10.79, -4.19; *P* = 8.76 x 10^-6^) with the mean differences ranging from -9.70% to -2.40%. Heterogeneity between studies was statistically highly significant (*τ^2^* = 9.84; *P* = 1.07 x 10^-4^; *I^2^* = 85.70%). C, subtype-combined sample.


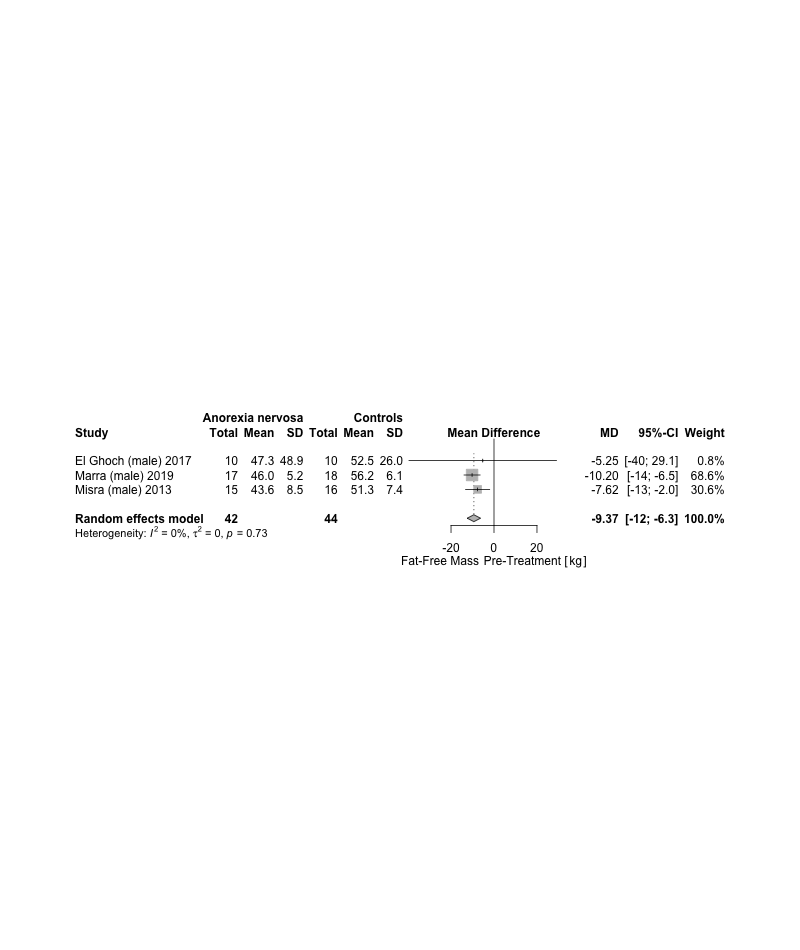


**Figure S95.** Cross-sectional meta-analysis of studies reporting fat-free mass in acutely-ill/pre-treatment male anorexia nervosa patients compared with healthy controls. Three samples had the appropriate data for the meta-analysis with 42 AN cases and 44 controls. A random-effects meta-analysis revealed a pooled estimate of the mean difference (MD: -9.37 kg; 95% CI: -12.47, -6.27; *P* = 3.30 x 10^-9^) with the mean differences ranging from -10.20 kg to -5.25 kg. Heterogeneity between studies was statistically highly significant (*τ^2^* = 0.00; *P* = 0.72; *I^2^* = 0%). C, subtype-combined sample.

**Adjustment for small study effects**

**9) Copas selection models**

**9a) Bone mineral density (whole body) pre-treatment**


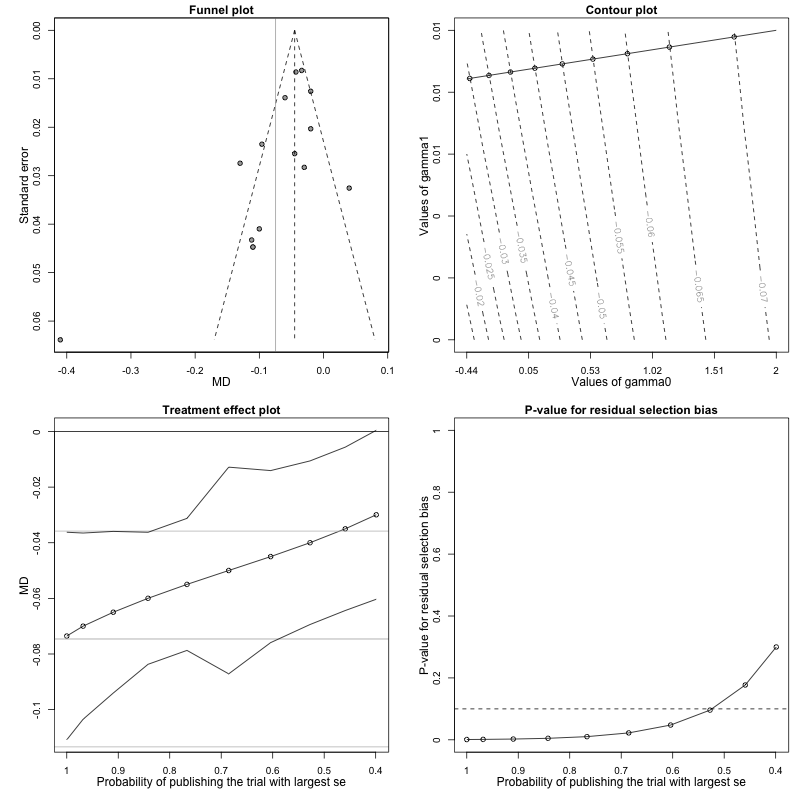


**Figure S96. Copas model bone mineral density (whole body) pre-treatment**

**Figure S96a.** Funnel plot for pre-treatment (acutely-ill AN) bone mineral density (whole body). The vertical gray line corresponds the random effects model estimate and the diagonal dashed lines correspond to ± 2 standard errors (SEs). If there is no heterogeneity and no publication bias, 95% of all studies should lie within the funnel.

**Figure S96b.** Contour plot. The plot shows the estimated treatment effect for a range of pairs (𝛾0 and 𝛾1). The Copas selection model has two sensitivity parameters: 𝛾0 on the x-axis is roughly the probit of the probability that a study with a small sample size (large standard error, SE) is published and 𝛾1 on the y-axis is roughly the regression coefficient of the change in the probit of the probability that a study with observed precision 1/SE is published. The line of steepest descent from the top right corner descending to the left indicates selection. Contours in this plot should be smooth.

**Figure S96c.** Effect plot. The effect estimates highlighted in the contour plot (b) are also shown in this plot including their 95% confidence intervals. Values on the x-axis are calculated using the largest standard error (SE) of all studies in the given meta-analysis (the “smallest study”) and values for 𝛾0 and 𝛾1 from the contour plot. Selection increases on the x-axis from the left to the right (because the probability of publishing the study with the largest SE decreases).

**Figure S96d.** P-value plot. The plot shows the p-value for residual selection bias. A p-value of p = 0.1 is regarded as significant indicated by the horizontal dashed line. If the contour of estimated effects crosses the dashed line, the adjusted effect can be read off at this point of selection.

The contour plot showed smooth contours with ranges for 𝛾_0_ and 𝛾_1_ being appropriate and the line orthogonal to the contours was well chosen (Figure 96b). The Copas model (i.e., maximum likelihood) estimate and its confidence interval with no publication bias (left hand end, solid line) agreed with the random effects estimate shown by the gray horizontal line. As the probability of publishing the smallest studies decreases the effect confidence intervals generally were getting narrower. The mean effect and its confidence intervals vary smoothly to the right of the area and the confidence intervals do not cut the null value (Figure 96c). The Copas model p-value for residual selection bias was significant with p = 0.001 and the relationship between the p-value for residual selection bias and the probability of publishing the smallest study (largest SE) was completely smooth indicating no local problems with the estimation. The Copas selection model analysis does not overturn the conclusion of the original meta-analysis comparing acutely-ill anorexia nervosa cases with healthy controls as the adjusted estimate of -0.03 g/cm^3^ (95% CI, -0.06, -0.01; p = 0.02) with an estimated selection probability of 46% and 11 potentially unpublished studies is halved compared with the random effects model estimate with -0.07 g/cm^3^ (95% CI, -0.11, -0.04) and has narrower confidence intervals. The point estimate was not contained in the original confidence interval.

**9c) Fasting glucose pre-treatment**


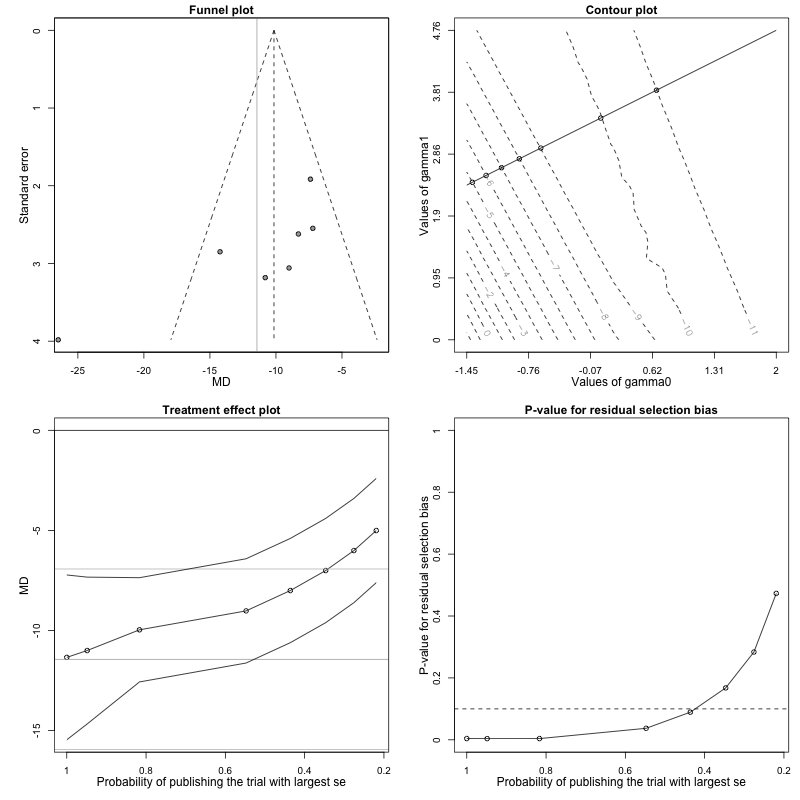


**Figure S97. Copas model fasting glucose pre-treatment**

**Figure S97a.** Funnel plot for pre-treatment (acutely-ill AN) fasting glucose. The vertical gray line corresponds the random effects model estimate and the diagonal dashed lines correspond to ± 2 standard errors (SEs). If there is no heterogeneity and no publication bias, 95% of all studies should lie within the funnel.

**Figure S97b.** Contour plot. The plot shows the estimated treatment effect for a range of pairs (𝛾0 and 𝛾1). The Copas selection model has two sensitivity parameters: 𝛾0 on the x-axis is roughly the probit of the probability that a study with a small sample size (large standard error, SE) is published and 𝛾1 on the y-axis is roughly the regression coefficient of the change in the probit of the probability that a study with observed precision 1/SE is published. The line of steepest descent from the top right corner descending to the left indicates selection. Contours in this plot should be smooth.

**Figure S97c.** Effect plot. The effect estimates highlighted in the contour plot (b) are also shown in this plot including their 95% confidence intervals. Values on the x-axis are calculated using the largest standard error (SE) of all studies in the given meta-analysis (the “smallest study”) and values for 𝛾0 and 𝛾1 from the contour plot. Selection increases on the x-axis from the left to the right (because the probability of publishing the study with the largest SE decreases).

**Figure S97d.** P-value plot. The plot shows the p-value for residual selection bias. A p-value of p = 0.1 is regarded as significant indicated by the horizontal dashed line. If the contour of estimated effects crosses the dashed line, the adjusted effect can be read off at this point of selection.

The contour plot showed smooth contours with ranges for 𝛾_0_ and 𝛾_1_ being appropriate and the line orthogonal to the contours was well chosen (Figure 97b). The Copas model (i.e., maximum likelihood) estimate and its confidence interval with no publication bias (left hand end, solid line) agreed with the random effects estimate shown by the gray horizontal line. As the probability of publishing the smallest studies decreases the effect confidence intervals generally were getting narrower. The mean effect and its confidence intervals vary smoothly to the right of the area and the confidence intervals do not cut the null value (Figure 97c). The Copas model p-value for residual selection bias was significant with p = 0.009 and the relationship between the p-value for residual selection bias and the probability of publishing the smallest study (largest SE) was completely smooth indicating no local problems with the estimation. The Copas selection model analysis does not overturn the conclusion of the original meta-analysis comparing acutely-ill anorexia nervosa cases with healthy controls as the adjusted estimate of -7.01 mg/dL (95% CI, -9.61, -4.40; p < 0.0001) with an estimated selection probability of 35% and 9 potentially unpublished studies is similar to the random effects model estimate with -11.44 mg/dL (95% CI, -15.95, -6.93) and has a comparable confidence interval. The adjusted point estimate has shrunken by a third and was contained in the original confidence interval.

**9d) Leptin pre-treatment**


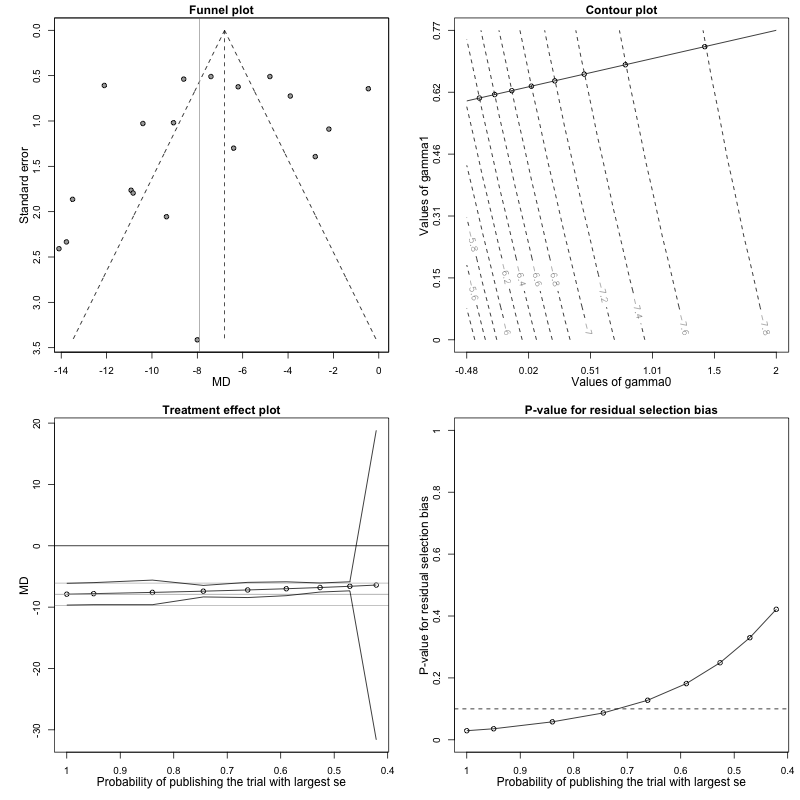


**Figure S98. Copas model leptin pre-treatment**

**Figure S98a.** Funnel plot for pre-treatment (acutely-ill AN) leptin. The vertical gray line corresponds the random effects model estimate and the diagonal dashed lines correspond to ± 2 standard errors (SEs). If there is no heterogeneity and no publication bias, 95% of all studies should lie within the funnel.

**Figure S98b.** Contour plot. The plot shows the estimated treatment effect for a range of pairs (𝛾0 and 𝛾1). The Copas selection model has two sensitivity parameters: 𝛾0 on the x-axis is roughly the probit of the probability that a study with a small sample size (large standard error, SE) is published and 𝛾1 on the y-axis is roughly the regression coefficient of the change in the probit of the probability that a study with observed precision 1/SE is published. The line of steepest descent from the top right corner descending to the left indicates selection. Contours in this plot should be smooth.

**Figure S98c.** Effect plot. The effect estimates highlighted in the contour plot (b) are also shown in this plot including their 95% confidence intervals. Values on the x-axis are calculated using the largest standard error (SE) of all studies in the given meta-analysis (the “smallest study”) and values for 𝛾0 and 𝛾1 from the contour plot. Selection increases on the x-axis from the left to the right (because the probability of publishing the study with the largest SE decreases).

**Figure S98d.** P-value plot. The plot shows the p-value for residual selection bias. A p-value of p = 0.1 is regarded as significant indicated by the horizontal dashed line. If the contour of estimated effects crosses the dashed line, the adjusted effect can be read off at this point of selection.

The contour plot showed smooth contours with ranges for 𝛾_0_ and 𝛾_1_ being appropriate and the line orthogonal to the contours was well chosen (Figure 98b). The Copas model (i.e., maximum likelihood) estimate and its confidence interval with no publication bias (left hand end, solid line) agreed with the random effects estimate shown by the gray horizontal line. As the probability of publishing the smallest studies decreases the effect confidence intervals generally were getting narrower. The mean effect and its confidence intervals vary smoothly to the right of the area and the confidence intervals do not cut the null value (Figure 98c). The Copas model p-value for residual selection bias was significant with p = 0.03 and the relationship between the p-value for residual selection bias and the probability of publishing the smallest study (largest SE) was completely smooth indicating no local problems with the estimation. The Copas selection model analysis does not overturn the conclusion of the original meta-analysis comparing acutely-ill anorexia nervosa cases with healthy controls as the adjusted estimate of -7.20 ng/mL (95% CI, -8.44, -5.96; p < 0.0001) with an estimated selection probability of 66% and 5 potentially unpublished studies is similar to the random effects model estimate with -7.90 ng/mL (95% CI, -9.72, -6.08) and has a comparable confidence interval.

**8f) Body mass index post-treatment**


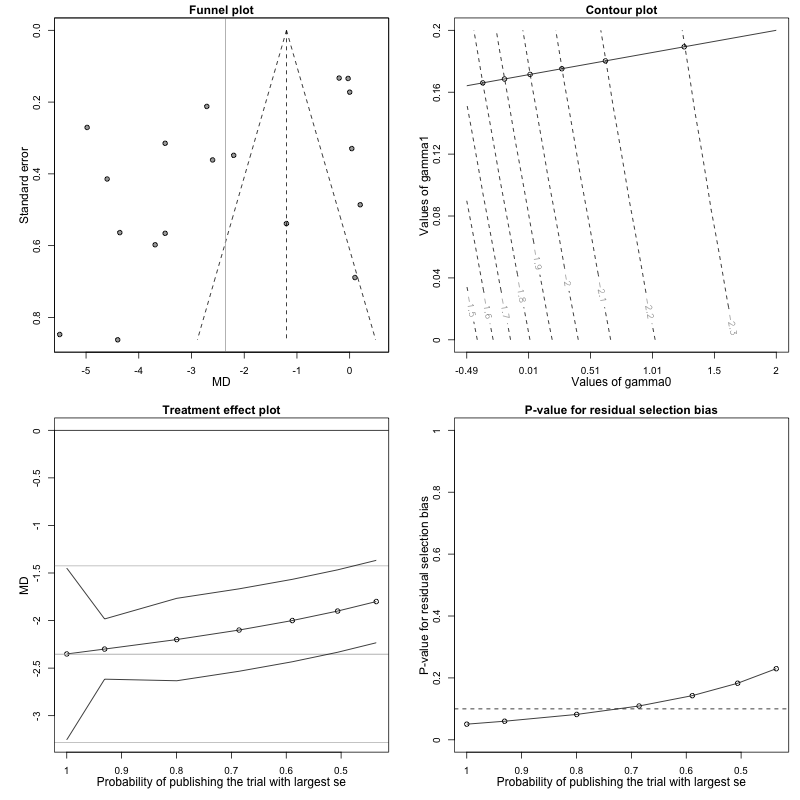


**Figure S99. Copas model body mass index post-treatment**

**Figure S99a.** Funnel plot for post-treatment body mass index. The vertical gray line corresponds the random effects model estimate and the diagonal dashed lines correspond to ± 2 standard errors (SEs). If there is no heterogeneity and no publication bias, 95% of all studies should lie within the funnel.

**Figure S99b.** Contour plot. The plot shows the estimated treatment effect for a range of pairs (𝛾0 and 𝛾1). The Copas selection model has two sensitivity parameters: 𝛾0 on the x-axis is roughly the probit of the probability that a study with a small sample size (large standard error, SE) is published and 𝛾1 on the y-axis is roughly the regression coefficient of the change in the probit of the probability that a study with observed precision 1/SE is published. The line of steepest descent from the top right corner descending to the left indicates selection. Contours in this plot should be smooth.

**Figure S99c.** Effect plot. The effect estimates highlighted in the contour plot (b) are also shown in this plot including their 95% confidence intervals. Values on the x-axis are calculated using the largest standard error (SE) of all studies in the given meta-analysis (the “smallest study”) and values for 𝛾0 and 𝛾1 from the contour plot. Selection increases on the x-axis from the left to the right (because the probability of publishing the study with the largest SE decreases).

**Figure S99d.** P-value plot. The plot shows the p-value for residual selection bias. A p-value of p = 0.1 is regarded as significant indicated by the horizontal dashed line. If the contour of estimated effects crosses the dashed line, the adjusted effect can be read off at this point of selection.

The contour plot showed smooth contours with ranges for 𝛾_0_ and 𝛾_1_ being appropriate and the line orthogonal to the contours was well chosen (Figure 99b). The Copas model (i.e., maximum likelihood) estimate and its confidence interval with no publication bias (left hand end, solid line) agreed with the random effects estimate shown by the gray horizontal line. As the probability of publishing the smallest studies decreases the effect confidence intervals generally were getting narrower. The mean effect and its confidence intervals vary smoothly to the right of the area and the confidence intervals do not cut the null value (Figure 99c). The Copas model p-value for residual selection bias was significant with p = 0.05 and the relationship between the p-value for residual selection bias and the probability of publishing the smallest study (largest SE) was completely smooth indicating no local problems with the estimation. The Copas selection model analysis does not overturn the conclusion of the original meta-analysis comparing post-treatment anorexia nervosa cases with healthy controls as the adjusted estimate of -2.10 kg/m^2^ (95% CI, -2.53, -1.67; p < 0.0001) with an estimated selection probability of 64% and 11 potentially unpublished studies is similar to the random effects model estimate with -2.35 kg/m^2^ (95% CI, -3.28, -1.42) and has a comparable confidence interval.

**8g) Body fat percentage post-treatment**


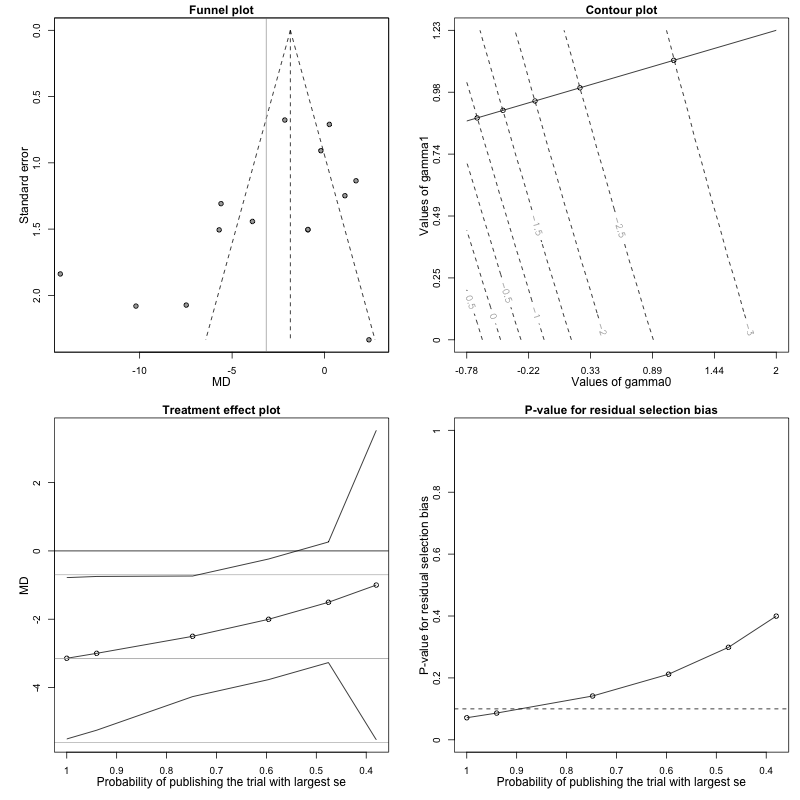


**Figure S100. Copas model body fat percentage post-treatment**

**Figure S100.** Funnel plot for post-treatment body fat percentage. The vertical gray line corresponds the random effects model estimate and the diagonal dashed lines correspond to ± 2 standard errors (SEs). If there is no heterogeneity and no publication bias, 95% of all studies should lie within the funnel.

**Figure S100b.** Contour plot. The plot shows the estimated treatment effect for a range of pairs (𝛾0 and 𝛾1). The Copas selection model has two sensitivity parameters: 𝛾0 on the x-axis is roughly the probit of the probability that a study with a small sample size (large standard error, SE) is published and 𝛾1 on the y-axis is roughly the regression coefficient of the change in the probit of the probability that a study with observed precision 1/SE is published. The line of steepest descent from the top right corner descending to the left indicates selection. Contours in this plot should be smooth.

**Figure S100c.** Effect plot. The effect estimates highlighted in the contour plot (b) are also shown in this plot including their 95% confidence intervals. Values on the x-axis are calculated using the largest standard error (SE) of all studies in the given meta-analysis (the “smallest study”) and values for 𝛾0 and 𝛾1 from the contour plot. Selection increases on the x-axis from the left to the right (because the probability of publishing the study with the largest SE decreases).

**Figure S100d.** P-value plot. The plot shows the p-value for residual selection bias. A p-value of p = 0.1 is regarded as significant indicated by the horizontal dashed line. If the contour of estimated effects crosses the dashed line, the adjusted effect can be read off at this point of selection.

The contour plot showed smooth contours with ranges for 𝛾_0_ and 𝛾_1_ being appropriate and the line orthogonal to the contours was well chosen (Figure 100b). The Copas model (i.e., maximum likelihood) estimate and its confidence interval with no publication bias (left hand end, solid line) agreed with the random effects estimate shown by the gray horizontal line. As the probability of publishing the smallest studies decreases the effect confidence intervals generally were getting narrower. The mean effect and its confidence intervals vary smoothly to the right of the area and the confidence intervals do not cut the null value (Figure 100c). The Copas model p-value for residual selection bias was significant with p = 0.07 and the relationship between the p-value for residual selection bias and the probability of publishing the smallest study (largest SE) was completely smooth indicating no local problems with the estimation. The Copas selection model analysis does not overturn the conclusion of the original meta-analysis comparing post-treatment anorexia nervosa cases with healthy controls as the adjusted estimate of -2.5% (95% CI, -4.3, -0.7; p = 0.005) with an estimated selection probability of 74% and 3 potentially unpublished studies is similar to the random effects model estimate with -3.2% (95% CI, -5.6, -0.7) and has a comparable confidence interval.

**8h) Trunk body fat percentage post-treatment**

**Figure S101. Copas model trunk body fat percentage post-treatment**

**Figure S101a.** Funnel plot for post-treatment body fat percentage. The vertical gray line corresponds the random effects model estimate and the diagonal dashed lines correspond to ± 2 standard errors (SEs). If there is no heterogeneity and no publication bias, 95% of all studies should lie within the funnel.

**Figure S101b.** Contour plot. The plot shows the estimated treatment effect for a range of pairs (𝛾0 and 𝛾1). The Copas selection model has two sensitivity parameters: 𝛾0 on the x-axis is roughly the probit of the probability that a study with a small sample size (large standard error, SE) is published and 𝛾1 on the y-axis is roughly the regression coefficient of the change in the probit of the probability that a study with observed precision 1/SE is published. The line of steepest descent from the top right corner descending to the left indicates selection. Contours in this plot should be smooth.

**Figure S101c.** Effect plot. The effect estimates highlighted in the contour plot (b) are also shown in this plot including their 95% confidence intervals. Values on the x-axis are calculated using the largest standard error (SE) of all studies in the given meta-analysis (the “smallest study”) and values for 𝛾0 and 𝛾1 from the contour plot. Selection increases on the x-axis from the left to the right (because the probability of publishing the study with the largest SE decreases).

**Figure S101d.** P-value plot. The plot shows the p-value for residual selection bias. A p-value of p = 0.1 is regarded as significant indicated by the horizontal dashed line. If the contour of estimated effects crosses the dashed line, the adjusted effect can be read off at this point of selection.

The contour plot showed smooth contours with ranges for 𝛾_0_ and 𝛾_1_ being appropriate and the line orthogonal to the contours was well chosen (Figure 101b). The Copas model (i.e., maximum likelihood) estimate and its confidence interval with no publication bias (left hand end, solid line) agreed with the random effects estimate shown by the gray horizontal line. As the probability of publishing the smallest studies decreases the effect confidence intervals generally were getting narrower. The mean effect and its confidence intervals vary smoothly to the right of the area and the confidence intervals do not cut the null value (Figure 101c). The Copas model p-value for residual selection bias was significant with p < 0.001 and the relationship between the p-value for residual selection bias and the probability of publishing the smallest study (largest SE) was completely smooth indicating no local problems with the estimation. The Copas selection model analysis overturns the conclusion of the original meta-analysis comparing post-treatment anorexia nervosa cases with healthy controls as the adjusted estimate of 12.0% (95% CI, 9.54; 14.44; p < 0.0001) with an estimated selection probability of 5% and 52 potentially unpublished studies is different to the random effects model estimate with 5.8% (95% CI, -0.14, 11.83) and has a much narrower confidence interval. The adjusted point estimated was not included in the original confidence interval.
